# TRIM23 prevents adenovirus replication by p62-mediated selective autophagic degradation of viral E1A protein

**DOI:** 10.1371/journal.ppat.1014578

**Published:** 2026-09-08

**Authors:** Nan Sun, Jikai Zhang, Banruo Xia, Hang Yin, Jiaqian Xie, Xiaoxiao Liu, Man Li, Lin Fang, Guijuan Ji, Gaolei Ma, Haihan Zhang, Zhenzhen Wang, Dafei Chai, Gang Wang

**Affiliations:** 1 Cancer Institute, Cellular Therapeutics School of Medicine, Xuzhou Medical University, Xuzhou, Jiangsu, China; 2 Center of Clinical Oncology, The Affiliated Hospital of Xuzhou Medical University, Xuzhou, Jiangsu, China; 3 Jiangsu Center for the Collaboration and Innovation of Cancer Biotherapy, Xuzhou Medical University, Xuzhou, Jiangsu, China; 4 Department of Pathogen Biology and Immunology, Jiangsu Key Laboratory of Immunity and Metabolism, School of Basic Medical Sciences, Xuzhou Medical University, Xuzhou, China; State University of New York Upstate Medical University, UNITED STATES OF AMERICA

## Abstract

The E3 ubiquitin ligase TRIM23 is involved in diverse cellular processes, however, its function in antiviral defense against adenovirus remains unclear. Here, we identify a novel mechanism by which TRIM23 restricts human adenovirus type 5 (HAdV-C5) replication. TRIM23 expression was upregulated upon HAdV-C5 infection, and functional studies showed that its overexpression inhibited viral replication, while knockdown enhanced it. Mechanistically, TRIM23 interacts with the viral E1A protein and promotes its degradation through a mechanism dependent of its canonical E3 ligase activity. Moreover, TRIM23 recruits the selective autophagy receptor p62 promotes E1A degradation in a E1A ubiquitination-independent manner. Our results unveil a novel host defense pathway-the TRIM23-E1A-p62 axis-that highlights the role of selective autophagy in antiviral immunity.

## Author summary

Elucidating virus-host interactions is critical for developing therapies against HAdV-C5. This study reveals that TRIM23 plays an antiviral role independent of its ubiquitination function. Depending on its E3 ligase activity, TRIM23 counteracts HAdV-C5 by directing viral E1A to autophagic degradation via p62, thereby circumventing typical ubiquitin-proteasome mediated signaling. These findings expand our understanding of how selective autophagy restricts viral infection and uncover a previously unrecognized layer of host defense centered on the TRIM23-E1A-p62 axis.

## Introduction

Human adenovirus (HAdV) is a small, non-enveloped DNA virus with a linear double-stranded genome that primarily infects terminally differentiated epithelial cells [1]. As the earliest expressed viral protein during infection, the early gene product early protein 1A (E1A) plays a central role in activating the viral transcription program and reprogramming the host cell environment to promote viral replication [2]. E1A interacts with a broad range of cellular regulators-including cell cycle controllers, transcription factors, and chromatin remodelers-to disrupt host homeostasis and drive viral gene expression [3,4]. Beyond its role in infection, E1A has also served as a valuable molecular probe for identifying key cellular regulators implicated in processes such as cancer biology, exemplified by proteins like FUBP1, Nek9, and RuvBL1 [5–7]. HAdV early proteins include those involved in viral genome replication, as well as those that promote viral replication by reorganizing cellular structures or block the host’s antiviral response, playing crucial roles throughout the viral life cycle.

Autophagy is an evolutionarily conserved lysosome-dependent degradation pathway that maintains cellular homeostasis by degrading intracellular materials such as proteins, organelles, and lipids [8,9]. Based on the mechanism of transporting cargo to lysosomes, autophagy can be classified into three types: microautophagy, chaperone-mediated autophagy, and macroautophagy. Macroautophagy is currently the most extensively studied form of autophagy (hereafter referred to collectively as autophagy) [10]. Its process encompasses autophagy initiation, extension and sealing of autophagosomes, maturation of autophagosomes and fusion with lysosomes, as well as degradation and recycling of autophagosomes. Selective autophagy, a specialized branch of this pathway, mediates the targeted removal of specific substrates such as damaged organelles or intracellular pathogens [11]. Furthermore, autophagy is gaining increasing recognition as a crucial mechanism in antimicrobial defense. In the context of viral infection, autophagy plays a dual role, either suppressing or promoting viral replication depending on the viral pathogen, host species, and cell type [8,12]. For instance, Porcine epidemic diarrhea virus (PEDV) induces autophagy via its N protein, degrading the host antiviral protein HNRNPA1 [13]. African swine fever virus (ASFV) activates mitochondrial autophagy through SQSTM1-associated autophagosomes, degrading mitochondrial membrane protein 70 (TOMM70) to suppress TOMM70-mediated innate immune responses [14]. Recent studies indicate that autophagy and the innate antiviral immune response (particularly the type I interferon [IFN] response) exhibit complex interrelationships, and several key molecules involved in innate immunity also serve as crucial regulators of autophagy [11,15,16].

TRIM proteins constitute a large family of RING-type E3 ubiquitin ligases that function as important regulators of innate immunity and cellular homeostasis [17,18]. Many TRIM proteins act as antiviral restriction factors, often through their E3 ligase activity, which mediates degradative or non-degradative ubiquitination of viral or host targets [19,20]. Notably, several TRIM family members, such as TRIM13 and TRIM25, have been shown to intersect with autophagy pathways to modulate viral infection and immune responses. Avian TRIM13 weakens the antiviral innate immune response by targeting MAVS for autophagic degradation [21]. TRIM25 impedes the degradation of protein aggregates during porcine reproductive and respiratory syndrome virus infection by inhibiting p62-mediated autophagy [22]. Additionally, TRIM5α acts as a limiting factor to mediate the antiviral effect of HIV-1 through autophagy and proteasome-mediated mechanisms [23,24]. TRIM23 has ubiquitin E3 ligase and GTPase activity, which can mediate cGAS-STING-dependent antiviral autophagy [25,26].

In this study, we identify TRIM23 as a novel host restriction factor against human adenovirus type 5 (HAdV-C5). We demonstrate that TRIM23 is upregulated during HAdV-C5 infection and facilitates the targeting of the viral E1A protein for degradation via p62-mediated selective autophagy. These findings reveal a previously undescribed antiviral mechanism in which TRIM23 bridges viral cargo to the autophagic machinery, expanding our understanding of TRIM protein functions in host defense.

## Materials and methods

### Ethics statements

This study was carried out in strict accordance with the recommendations of the’ Laboratory Animal Care and Use Guidelines’ of the Ministry of Science and Technology of the People’s Republic of China. The mouse experiment was approved by the Animal Experimental Ethics Committee of Xuzhou Medical University (Approval No. 202505T034).

### Mice

Male BALB/c nude mice (6 weeks old) were maintained under specific-pathogen-free (SPF) conditions. All animal experiments were approved by the Animal Experimental Ethics Committee of Xuzhou Medical University and performed in accordance with institutional guidelines.

### Cell lines and adenoviruses

Human embryonic kidney cells (HEK293 and HEK293T), lung adenocarcinoma cells (A549), hepatocellular carcinoma cells (HepG2), renal carcinoma cells (786-O), and prostate cancer cells (PC-3) were cultured in Dulbecco’s modified Eagle’s medium (DMEM) supplemented with 10% fetal bovine serum (FBS) at 37°C under 5% CO_2_. All cell lines were regularly tested for mycoplasma contamination.

Wild-type human adenovirus type 5 (HAdV-C5) and its GFP-expressing variant (HAdV-C5-GFP) were propagated in A549 or HEK293 cells. The oncolytic adenovirus (OAV) was derived from HAdV-C5 with a deletion in the E1B55K gene [27,28]. Viral titers were determined by tissue culture infectious dose 50 (TCID₅₀) assay and expressed as plaque-forming units per milliliter (PFU/mL).

### Plasmids and antibodies

The coding sequences of full-length TRIM23 were cloned into the pcDNA3.1 vector with a C-terminal V5 tag. TRIM23 truncation mutants were generated by PCR-based mutagenesis and verified by DNA sequencing.

Primary antibodies used were: anti-E1A (Millipore; 05-599), anti-TRIM23 (Proteintech; 12607-1-AP), anti-V5 (Sigma-Aldrich; V8012, V8137), anti-Flag (Sigma-Aldrich; F1804, F7425), anti-Myc (Proteintech; 60003-2-Ig, 16286-1-AP), anti-GAPDH (Proteintech; 10494-1-AP, 60004-1-Ig), and mouse IgG (Beyotime; A7028). Alexa Fluor 633-conjugated goat anti-rabbit IgG (H + L) and Alexa Fluor 488-conjugated donkey anti-mouse IgG (H + L) were procured from Life Technologies. DyLight 800 goat anti-mouse IgG (H + L) and DyLight 680 goat anti-rabbit IgG (H + L) were obtained from Immunoway.

### Recombinant oncolytic adenovirus construction

The OAV used is a recombinant adenovirus in which the E1B55K gene has been deleted while the E1B19K gene has been retained, using human adenovirus subtype C serotype 5 as the vector backbone. Additionally, the tumor-specific promoter Ki67 is used to drive the adenoviral E1A gene. OAV-shTRIM23 was constructed by inserting a shTRIM23 expression cassette into the adenovirus E1 region. In brief, the TRIM23 shRNA, synthesized by Azenta Life Sciences and driven by a U6 promoter, was cloned into the adenovirus shuttle plasmid pZD55, from which the E1B55K gene had been deleted. HEK293 cells were co-transfected with pZD55-shTRIM23 (2 μg) and the adenoviral backbone plasmid pBHGE3 (2 μg) (Microbix Biosystems Inc.) using Lipofectamine 2000 (Invitrogen) according to the manufacturer’s instructions. The engineered adenovirus AdV5-ZD55-shTRIM23, designated OAV-shTRIM23, was amplified using HEK293 cells. The viral supernatant was harvested upon the appearance of cytopathic effects, and the viral titer was determined using the TCID50 assay.

### Construction of A549 cells stably overexpressing TRIM23

Lipofectamine 2000 was used to transfect the retroviral construct pQCXIN-TRIM23 and the empty vector pQCXIN into the AmphoPack-293 packaging cell line (631505, Clontech). Forty-eight hours after transfection, collect the viral supernatant from the transfected cells and use it to transduce A549 cells cultured in a six-well plate. Repeat this process 48 hours after the initial transduction. Pass the transduced cells and screen them in medium supplemented with 1,000 μg/mL G418. Clone the surviving cells into a 96-well plate for expansion and detect TRIM23 overexpression via quantitative reverse transcription PCR (RT-qPCR).

### Cell treatment

To inhibit proteasomal degradation, cells were treated with 10 µM MG132. Autophagy was inhibited using 10 mM 3-methyladenine (3-MA), 10 µM chloroquine (CQ), or 20 mM bafilomycin A1 (BafA1). Autophagy was induced by incubating cells in Earle’s Balanced Salt Solution (EBSS).

### Western blotting

The whole cell extract was prepared and reduced SDS-PAGE was performed on 10% polyacrylamide gel. Electrophoresis gel and transfered the protein to polyvinylidene fluoride (PVDF) membrane. The membrane was blocked at room temperature for 2 hours with 5% skim milk in TBS-T (Tris-buffered saline with Tween 20, 0.1%). After washing with PBS, the membrane was incubated with the corresponding primary antibody at room temperature for 2 hours, followed by incubation with immunofluorescent labeled secondary antibody at room temperature for 1 hour. Results were visualized using the Odyssey system.

### *In vivo* study

PC-3 cells (2 × 10⁶) mixed with Matrigel were subcutaneously injected into the flanks of nude mice. When tumor volume reached approximately 50–100 mm^3^, mice were randomly divided into three groups (n = 10 per group) and intratumorally injected with PBS, OAV, or OAV-shTRIM23 (1 × 10⁹ PFU per mouse) on days 1, 3, and 5. Tumor dimensions were measured every other day, and volume was calculated as (length × width^2^)/2. Mice were euthanized when tumor volume exceeded 2500 mm^3^ or if ulceration occurred. All experiments with animals were conducted in strict compliance with the principles of humanity in accordance with the Animal Experimental Ethics Committee of Xuzhou Medical University.

### RT-qPCR

Total RNA was extracted from cells using TRIzol reagent following the manufacturer’s protocol. For reverse transcription-qPCR experiments, cDNA synthesis was performed using the HiScript II 1st Strand cDNA Synthesis Kit (Vazyme, R232-01), followed by qPCR detection with the ChamQ Universal SYBR qPCR Master Mix (Vazyme, Q711). Target gene expression levels were normalized against the housekeeping gene GAPDH. All experiments were replicated at least three times. Relative mRNA expression levels were quantified using the 2^-ΔΔCt^ method. The primers are as follows: *E1A-forward: TTGTCATTATCACCGGAGGAA*, *E1A-reverse*: *TCACCCACTGCCCATAATTT*; *Hexon-forward*: *GGACGCCTCGGAGTACCTGAG*, *Hexon-reverse*: *ACAGTGGGGTTTCTGAACTTGTT*; *DBP-forward*: *TAATCAAGCATGGCAAAGGAG*, *DBP-reverse*: *AATTTCACTTTCCGCTTCG; GAPDH-forward*: *AGATCCCTCCAAAATCAAGTGG*, *GAPDH-reverse*: *GGCAGAGATGATGACCCTTTT*.

### TCID50 assay

Serially diluted virus samples were inoculated onto HEK293 cells in 96-well plates. After 7–10 days, cytopathic effect was assessed microscopically. Viral titer (TCID₅₀/mL) was calculated using the Reed-Muench method [29].

### Mass spectrometry

Digest proteins (250 µg per sample) in whole-cell lysates according to the FASP protocol. In brief, remove detergents, DTT, and other low-molecular-weight components using 100 µL of UA buffer via centrifugation-assisted repeated ultrafiltration. The protein suspension was added to 100 µL of 25 mM NH_4_HCO_3_, 5 µg of trypsin was added, and digestion was performed overnight at 37°C. After digestion, peptides from each sample were desalted on a C18 column, concentrated by vacuum centrifugation, and resuspended in 40 µL of 0.1% (vol/vol) formic acid. Liquid chromatography-tandem mass spectrometry (LC-MS/MS) analysis was performed using a Q-Exactive mass spectrometer (Thermo Fisher Scientific) coupled with an Easy nLC system (Proxeon Biosystems, now part of Thermo Fisher Scientific). The analysis duration was 120 minutes, with technical support provided by Shanghai Applied Protein Technology (Shanghai, China).

Mass spectrometry data were analyzed using MaxQuant software (version 1.6.14) and aligned against the UniProt proteome database. Quantification was performed using a label-free quantification algorithm. Quantifiable proteins were defined as those identified at least twice across three biological replicates. Proteins with an adjusted p-value < 0.05 and a fold change in abundance > 2 were identified as differentially expressed proteins between the empty vector and the adenovirus E1A overexpression (**S1 Table**).

### Statistical analysis

Data are presented as mean ± SEM from at least three independent experiments. Two-tailed Student’s *t*-test or analysis of variance was used for statistical analysis, *P < 0.05, **P < 0.01, ***P < 0.005, ****P < 0.0001, N.S., not statistically significant.

## Results

### TRIM23 inhibits HAdV-C5 replication

**Fig 1:**
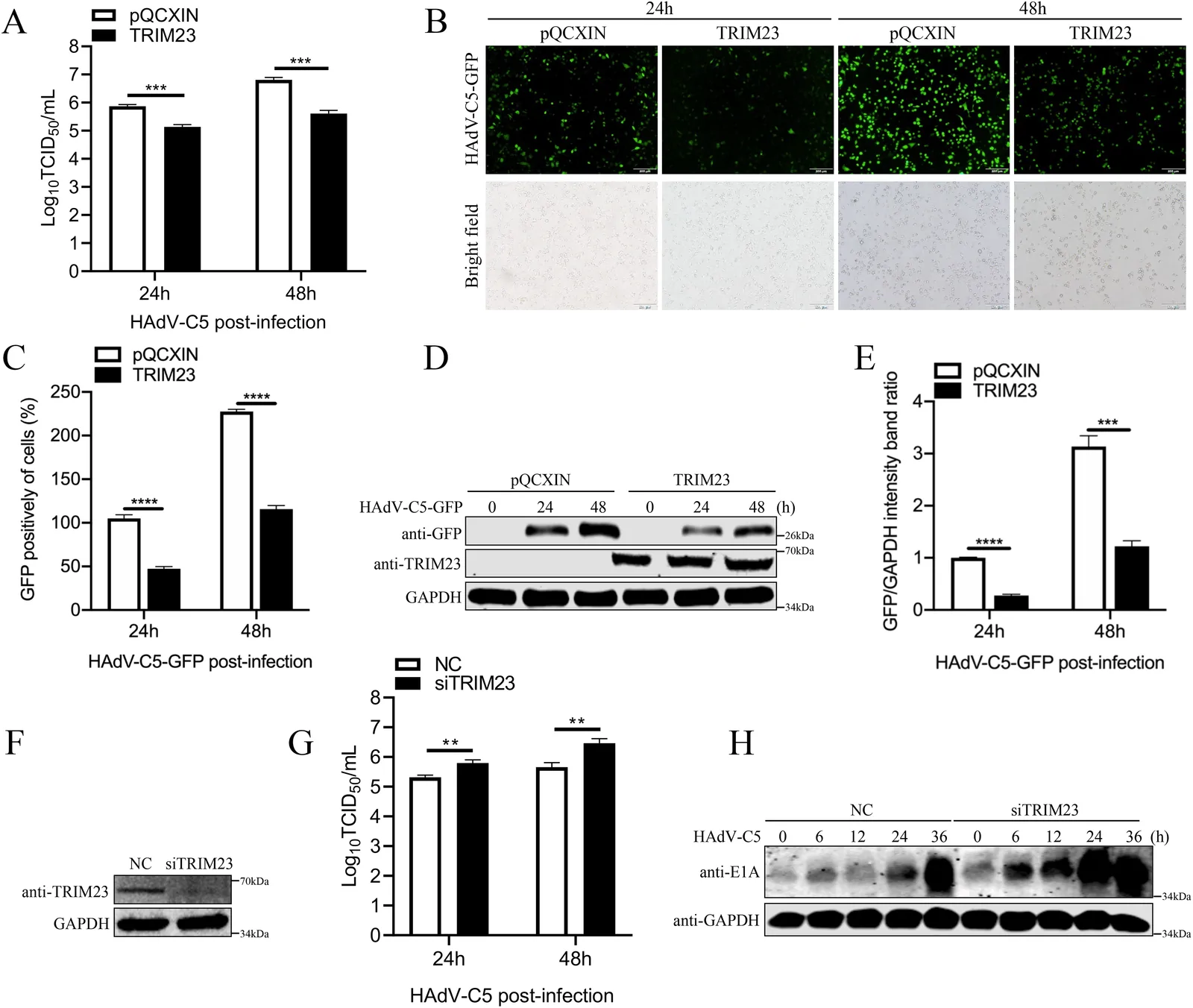
TRIM23 inhibits HAdV-C5 replication. **(A)** A549 cells stably overexpressing TRIM23 and control cells were infected with HAdV-C5 at an MOI of 5. Cell supernatants were collected 24- or 48-hours post-infection. Viral titers in infected cell supernatants were determined by the TCID50 assay. ***, *P* < 0.001. **(B and C)** Fluorescent intensity and cell number were observed under fluorescence microscopy in TRIM23 stably overexpressed A549 cells and control cells for 24 or 48 hours after infection with HAdV-C5-GFP. The GFP-positive rate was subsequently calculated using ImageJ software. Scale bar, 200 µm. ****, *P* < 0.0001. **(D and E)** GFP expression was detected by western blotting in TRIM23 stably overexpressed A549 cells and control cells infected with HAdV-C5-GFP at an MOI of 5 for 24 or 48 hours. Quantification of relative GFP levels is shown in **(E)**. ***, *P* < 0.001; ****, *P* < 0.0001. **(F)** Western blot analysis using anti-TRIM23 antibody was performed in A549 cells 36 hours after transfection with siRNA targeting TRIM23. **(G)** A549 cells transfected with siRNA targeting TRIM23 for 36 hours were infected with HAdV-C5 at an MOI of 5 for 24 or 48 hours. Viral titers in infected cell supernatants were determined by the TCID50 assay. **, *P* < 0.01. **(H)** TRIM23-knockdown or control A549 cells were infected with HAdV-C5 at an MOI of 10. Cells were harvested at the indicated time points and analyzed by western blot using an anti-E1A antibody. At least three independent experiments were conducted.

Our previous reports revealed significant variation in differentially expressed proteins (DEPs) observed in E1A-overexpressing cells [27,28]. Through proteomic analysis, several E1A-associated regulated proteins (including TRIM23) were selected for in-depth investigation (**S1 Table**). To investigate the role of TRIM23 in adenovirus infection, we first established TRIM23-stably overexpressing A549 cells, then infected with HAdV-C5 at a multiplicity of infection (MOI) of 5. Viral replication was assessed 24- or 48-hours post-infection using TCID50 assays, western blotting, and immunofluorescence techniques. Progeny virus yield was markedly decreased (Fig 1A). Cell viability assays confirmed that TRIM23 overexpression did not affect cellular viability (S1A Fig). The fluorescence intensity, number of infected cells, and GFP levels of HAdV-C5-GFP were significantly reduced in TRIM23-overexpressing cells (Fig 1B–1E).

Conversely, western blot analysis revealed significantly reduced TRIM23 protein levels in A549 cells transfected with TRIM23-targeting siRNA for 48 hours compared to controls (Fig 1F), with no significant difference in cell viability between TRIM23 siRNA-interfered cells and controls (S1B Fig). Transient interference of TRIM23 significantly promoted HAdV-C5 replication, resulting in more severe cytopathic effects and higher viral titers (Figs 1G and S1C). Further, we examined changes in E1A protein levels in TRIM23-interfered A549 cells at 0, 6-, 12-, 24-, and 36-hours post-infection. Results revealed that TRIM23 interference significantly elevated levels of the viral E1A protein over time (Fig 1H). These results together indicate that TRIM23 acts as a negative regulator of HAdV-C5 replication.

Next, we examined whether TRIM23 affects viral gene expression. Quantitative RT-qPCR revealed a progressive, time-dependent decrease in viral E1A, Hexon, and DBP mRNA levels in cells overexpressing TRIM23 during HAdV-C5 infection (S2A–S2C Fig). RT-qPCR validation confirmed the effect of TRIM23 overexpression (S2D Fig). Conversely, in TRIM23-knockdown cells, transcript levels of viral E1A, Hexon, and DBP were significantly elevated (S2E–S2H Fig). These findings suggest that TRIM23 attenuates viral gene expression at the transcriptional level.

### TRIM23 interacts with E1A

**Fig 2:**
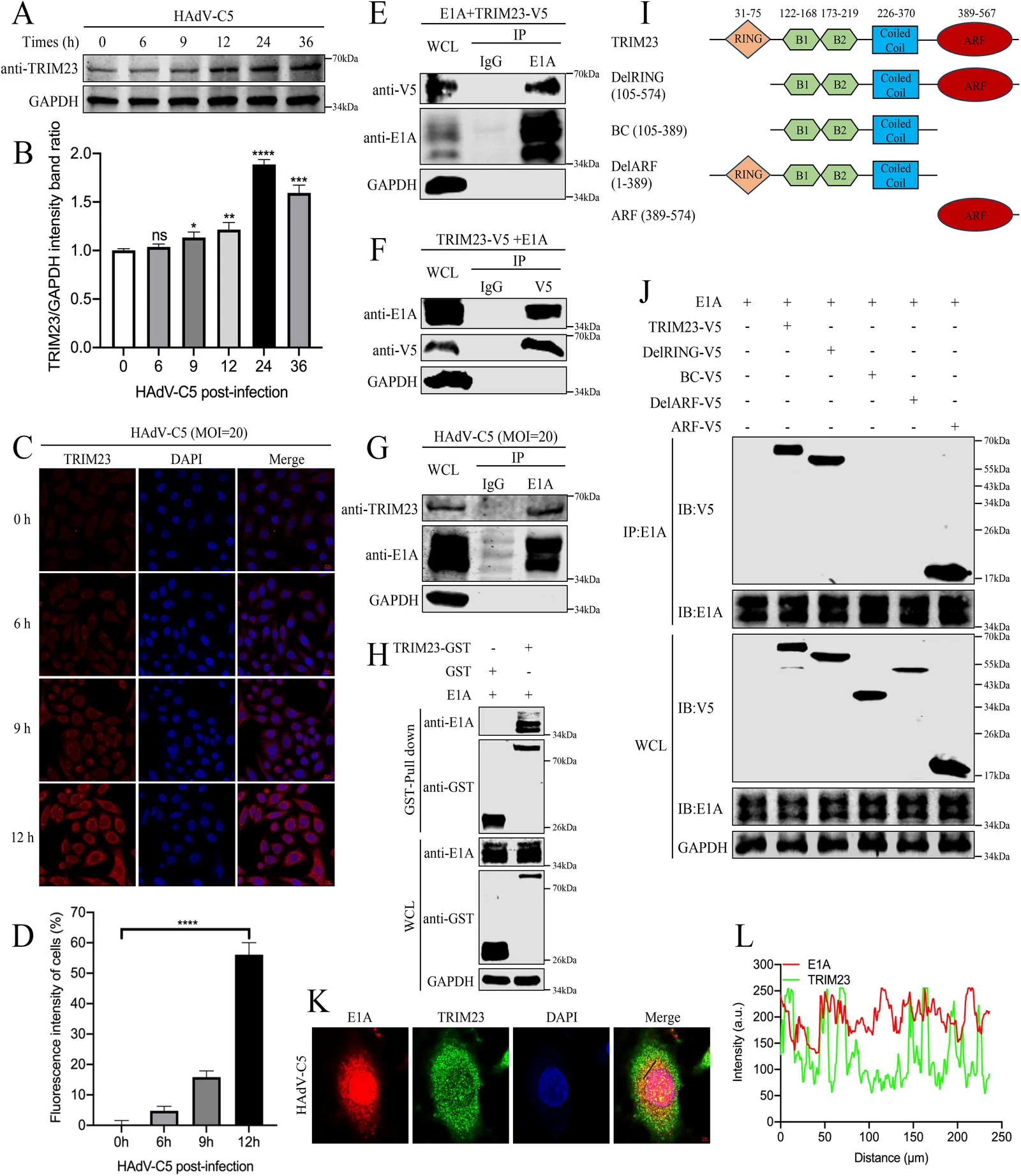
TRIM23 interacts with E1A. **(A and B)** Endogenous TRIM23 was detected by western blot analysis in A549 cells at 0, 6-, 9-, 12-, 24-, and 36-hours post-infection with HAdV-C5 at a multiplicity of infection (MOI) of 20. Quantification of relative TRIM23 levels is shown in **(B)**. *, *P* < 0.05; **, *P* < 0.01; ***, *P* < 0.001; ****, *P* < 0.0001. **(C and D)** Endogenous TRIM23 localization observed by Zeiss LSM880 microscopy in A549 cells infected with HAdV-C5 (MOI = 20) at 0-, 6-, 9-, and 12-hours post-infection. Relative fluorescence intensity of cells shown in panel **(D)**. Scale bar, 20 μm. ****, *P* < 0.0001. **(E and F)** HEK293T cells co-transfected with E1A and V5-tagged TRIM23 plasmids for 48 hours were subjected to co-immunoprecipitation using an anti-E1A antibody (E) and an anti-V5 antibody **(F)**, followed by western blot analysis. **(G)** Western blot analysis of A549 cell lysates co-immunoprecipitated with an anti-E1A antibody after 30 hours of infection with HAdV-C5 (MOI = 20). **(H)** GST or GST-TRIM23 was transfected into HEK293T cells. Thirty-six hours after transfection, cell lysates were collected and incubated with Glutathione Sepharose 4 Fast Flow for 6 hours. Cell lysates from cells expressing E1A were then added, and western blot analysis was performed. **(I and J)** Schematic diagram of TRIM23 domains in **(I)**. Co-immunoprecipitation and western blot analysis using an anti-E1A antibody after transfecting HEK293T cells with V5-tagged TRIM23 and its truncated mutants along with E1A. **(K and L)** A549 cells infected with HAdV-C5 at an MOI of 20 for 14 hours were fixed and analyzed by immunofluorescence using anti-E1A and anti-TRIM23 antibodies, imaged using a Zeiss LSM880 microscope. Both proteins significantly colocalized in the cytoplasm around the nucleus, as shown in **(K)**. Scale bar, 5 μm. The correlation coefficient of E1A (red) and TRIM23 (green) was shown in **(L)**. Three times independent experiments were conducted.

To elucidate the unresolved role of TRIM23 in antiviral responses, we sought to investigate its functional mechanisms during viral infection. First, we examined whether HAdV-C5 infection modulates TRIM23 expression levels. To this end, we infected A549 cells with HAdV-C5 and quantified TRIM23 protein levels and its intracellular localization via western blotting and immunofluorescence techniques. Immunoblotting analysis revealed that TRIM23 protein levels exhibited a progressive increase over time during HAdV-C5 infection (Fig 2A and 2B). In line with this, confocal microscopy observed an increase in TRIM23 in the cytoplasm during HAdV-C5 infection (Fig 2C and 2D).

In addition, we detected the changes of TRIM23 and viral E1A mRNA levels during HAdV-C5 infection. The results showed that the transcription level of TRIM23 increased first and then decreased during HAdV-C5 infection, which may be associated with elevated expression of the viral E1A protein (S3A and S3B Fig). Furthermore, contrary to many TRIM proteins that are lowly expressed under normal conditions and are induced by type I IFN [26,30], the mRNA and protein levels of TRIM23 are not up-regulated after IFN-α stimulation (S3C and S3D Fig). Interestingly, we observed that TRIM23 expression levels decreased progressively as the amount of E1A transfected increased, which is consistent with previous mass spectrometry results (S3E Fig and S1 Table). This may be due to the virus entering the cell and engaging in a struggle with the host to evade the cellular defense.

Given the effect of TRIM23 on E1A protein levels, we investigated whether TRIM23 interacts directly with E1A. Co-transfection of E1A and V5-TRIM23 plasmids into HEK293T cells followed by immunoprecipitation using anti-E1A or anti-V5 antibodies demonstrated a specific interaction between E1A and TRIM23 (Fig 2E and 2F). Endogenous co-IP in HAdV-C5-infected A549 cells further demonstrated that native TRIM23 binds to E1A during viral infection (Fig 2G). GST pull-down assay also established the interaction of TRIM23-E1A in vitro (Fig 2H). To map the interaction domain, we generated a series of TRIM23 truncation mutants spanning amino acids delRING (105–574 aa), BC (105–389 aa), delARF (1–389 aa), and ARF (389–574 aa) for co-IP assays (Fig 2I). As shown in Fig 2J, E1A was found to specifically interact with a TRIM23 fragment containing the ARF domain (389–574 aa). Immunofluorescence analysis confirmed cytoplasmic co-localization of TRIM23 and E1A (Fig 2K and 2L). Together, these results demonstrate that the ARF domain of TRIM23 mediates a physical interaction with HAdV-C5 E1A.

### TRIM23 activates autophagy to degrade HAdV-C5 E1A

**Fig 3:**
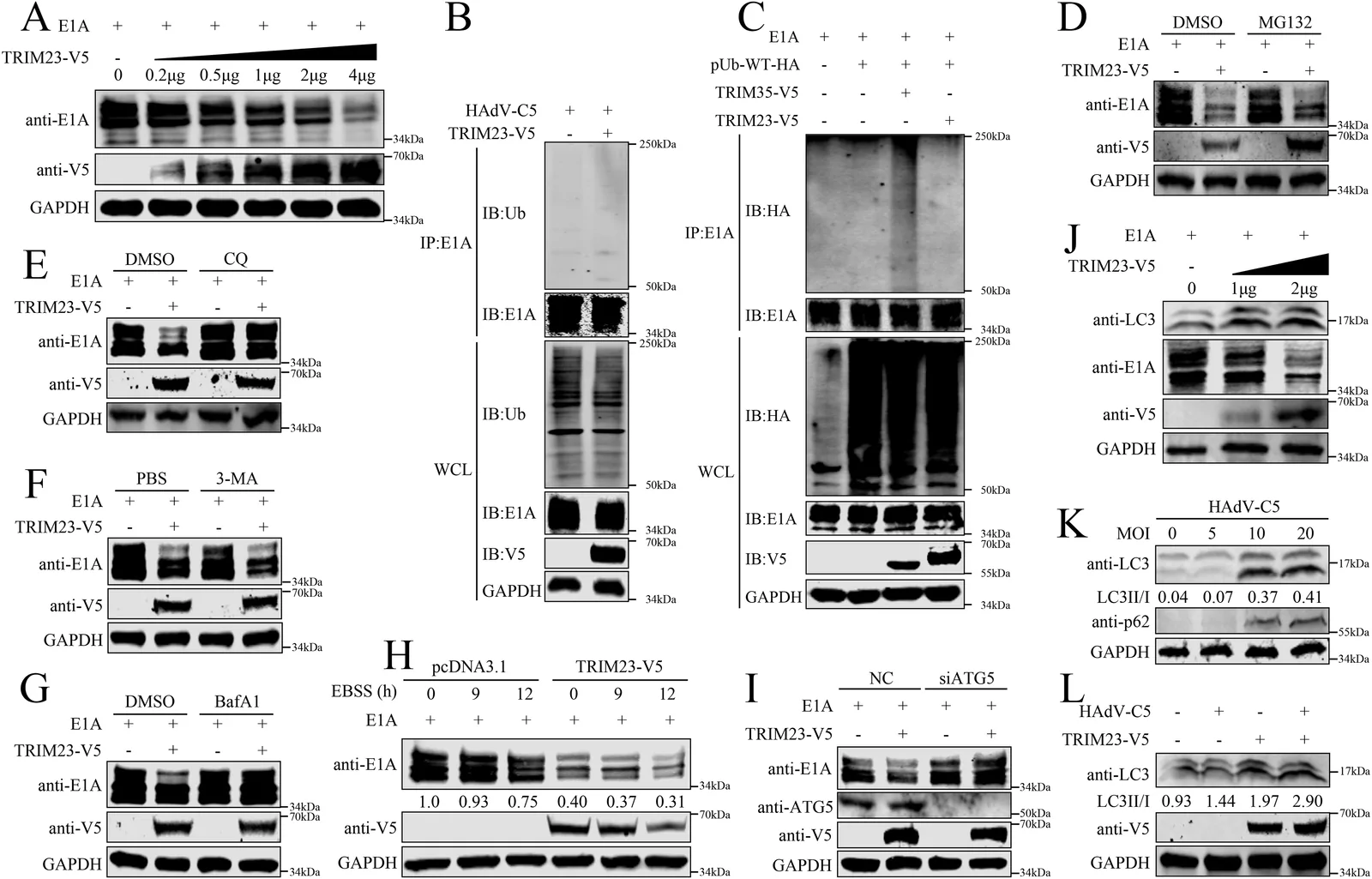
TRIM23 activates autophagy to degrade HAdV-C5 E1A. **(A)** Immunoblotting of protein extracts from HEK293T cells co-transfected with E1A-expressing plasmid and varying concentrations of V5-tagged TRIM23. **(B)** A549 cells transfected with V5-tagged TRIM23 or pcDNA3.1 were infected with HAdV-C5 at MOI = 10 for 30 hours. Co-immunoprecipitation with anti-E1A antibody followed by western blot analysis with the indicated antibodies. **(C)** HEK293T cells were cotransfected with E1A, TRIM35-V5, TRIM23-V5, and pHA-Ub WT. TRIM35-V5 serves as a positive control. Then, ubiquitination assays were performed and blotted with indicated antibodies. **(D, E, F, and G)** HEK293T cells co-transfected with V5-tagged TRIM23 and E1A for 24 hours were treated with MG132 (10 μM) **(D)**, CQ (10 μM) **(E)**, 3-MA (10 mM) **(F)**, or BafA1 (20 mM) (G) for 12 hours, followed by western blot analysis with the indicated antibodies. **(H)** HEK293T cells were co-transfected with E1A and V5-tagged TRIM23 or empty vector for 24 hours, followed by treatment with EBSS for specified durations, then analyzed by western blot using the indicated antibodies. **(I)** HEK293T cells co-transfected with E1A and V5-tagged TRIM23, with or without siATG5, followed by western blot analysis at 36 hours using the indicated antibodies. **(J)** A549 cells were transfected with E1A and either empty vector or plasmids expressing V5-tagged TRIM23 at different concentrations, followed by western blot analysis using the indicated antibodies. **(K)** A549 cells were infected with HAdV-C5 at MOIs of 0, 5, 10, and 20 for 24 hours, followed by western blot analysis using anti-LC3 and anti-p62 antibodies. **(L)** A549 cells overexpressing TRIM23, or control cells were infected with HAdV-C5 at MOI = 10 for 24 hours, followed by western blot analysis using anti-LC3 antibody. At least three independent experiments were conducted.

To validate these findings, E1A expression plasmids were cotransfected with V5-tagged TRIM23 expression plasmids at varying concentrations into HEK293T cells. As shown in Fig 3A, E1A expression exhibited a dose-dependent decrease with increasing TRIM23 concentration. To further investigate whether TRIM23 mediates E1A ubiquitination as an E3 ligase, we examined the ubiquitination status of E1A under TRIM23 regulation. Co-immunoprecipitation experiments revealed no detectable change in the polyubiquitination levels of E1A following TRIM23 overexpression (Fig 3B and 3C), suggesting that it was not dependent on the ubiquitin pathway.

The degradation of eukaryotic proteins primarily occurs through three pathways: apoptosis, ubiquitin-proteasome system, and autophagy-lysosomal system [12,31]. To identify the TRIM23-mediated E1A degradation pathway, we treated HEK293T cells co-expressing E1A and V5-TRIM23 with pathway-specific inhibitors, including the proteasome inhibitor MG132 and autophagy inhibitors 3-methyladenine (3-MA), chloroquine (CQ), and Bafilomycin A1 (BafA1). Notably, two distinct autophagy inhibitors (CQ and BafA1) both reversed E1A degradation, whereas the proteasome inhibitor (MG132) and autophagy inhibitor (3-MA) had no effect (Fig 3D–3G).

Next, we induced autophagy through starvation in Earle’s balanced salt solution (EBSS) culture conditions and found that this treatment synergistically enhanced TRIM23 degradation (Fig 3H). Furthermore, knocking down the autophagy regulator ATG5 reversed the E1A reduction mediated by TRIM23 (Fig 3I). Additionally, we examined the effect of TRIM23 on autophagy levels in E1A-expressing cells. Co-expression of E1A and V5-TRIM23 in A549 cells resulted in a dose-dependent increase in LC3-I to LC3-II conversion, suggesting TRIM23 may elevate autophagy levels in E1A-expressing cells (Fig 3J). Immunoblotting analysis of LC3II/I and p62 levels in A549 cells infected with different MOIs demonstrated that HAdV-C5 activates autophagy (Fig 3K). Further, TRIM23 promotes virus-induced autophagy (Fig 3L). Collectively, these results indicate that TRIM23 functions by promoting the autophagic degradation of E1A.

### TRIM23 restricts HAdV-C5 replication through an E3 ligase-dependent mechanism

**Fig 4:**
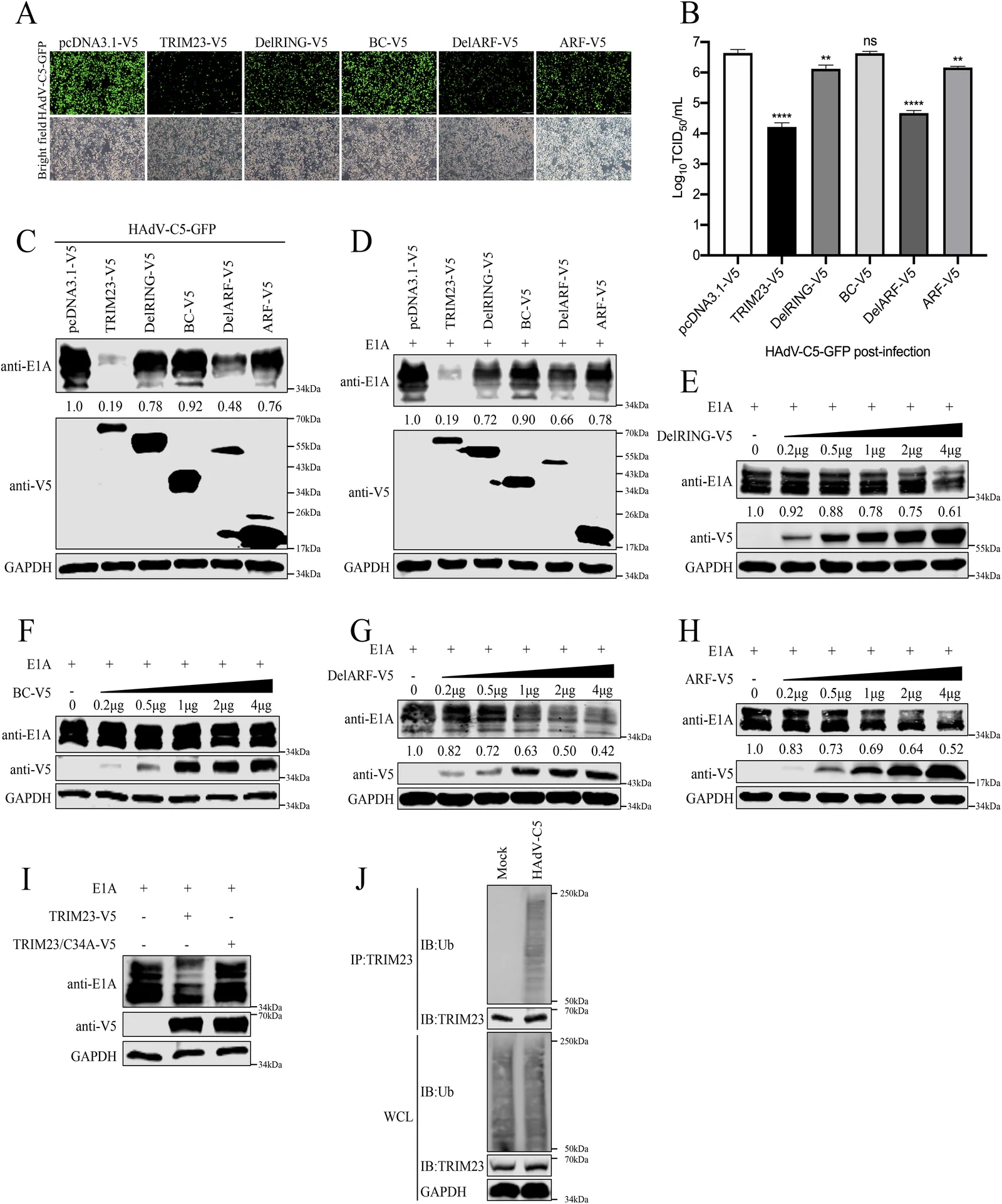
TRIM23 restricts HAdV-C5 replication through an E3 ligase-dependent mechanism. **(A, B and C)** Wild-type TRIM23 and its various truncation mutants were separately transfected into A549 cells, followed by infection with HAdV-C5-GFP for 36 hours. Fluorescence was observed under a fluorescence microscope and TCID50 assays were performed. In addition, western blot was used to detect the E1A viral protein in cells. Scale bar, 200 μm. **, *P* < 0.01; ****, *P* < 0.0001. **(D)** V5-tagged TRIM23 and its mutants (DelRING, BC, DelARF, and ARF) were co-transfected with E1A into HEK293T cells for 48 hours, followed by western blot analysis. **(E, F, G, and H)** Immunoblotting of protein extracts from HEK293T cells co-transfected with E1A-expressing plasmid and varying concentrations of V5-tagged TRIM23 truncated mutants (DelRING, BC, DelARF, or ARF), separately. **(I)** V5-tagged wild-type TRIM23 and C34A mutant were co-transfected with E1A into HEK293T cells for 48 hours, followed by western blot analysis. **(J)** Ubiquitination of endogenous TRIM23 in A549 cells that were either mock or infected with HAdV-C5 for 18 h, determined by IP with anti-TRIM23 and western blot analysis using anti-Ub antibody. At least three independent experiments were conducted.

A key feature of TRIM proteins is that its N-terminal RING finger domain has E3 ligase activity [32]. In addition, TRIM proteins possess various C-terminal domains, which typically mediate protein-protein interactions but may also possess enzymatic activity [26]. Considering that TRIM23 belongs to the E3 ubiquitin ligase family and possess a C-terminal ARF domain, which exhibits GTPase activity, we next investigated whether its enzymatic activity mediates antiviral function. First, we explored which domain of TRIM23 is involved in antiviral during HAdV-C5 infection. Following overexpression of wild-type TRIM23 and its various truncated mutants in A549 cells, followed by HAdV-C5-GFP infection for 36 hours, fluorescence analysis and TCID50 assays revealed that overexpression of wild-type TRIM23 significantly inhibited HAdV-C5-GFP replication. However, when only the B-Box and Coil-coil domains were present, no antiviral effect was observed, and there was no significant difference in viral E1A protein level compared with the control, with viral titers showing no significant difference compared to the control (Fig 4A–4C). These results indicated that the RING domain of TRIM23 plays a major role in antiviral activity, and the ARF domain also has a little antiviral effect, and the two together assist the antiviral activity to achieve the best.

In line with this, E1A was cotransfected with the V5-tagged TRIM23 and its various mutants into HEK293T cells to determine the domain of TRIM23 involved in the degradation of E1A. The results indicate that, in the presence of only the BC domain, the E1A protein showed virtually no difference compared to the control (Fig 4D). Further, E1A expression plasmids were cotransfected with V5-tagged TRIM23 mutants (DelRING, BC, DelARF, or ARF) expression plasmids at varying concentrations into HEK293T cells, separately. The results showed that the level of the viral E1A protein remained unchanged when only the BC domain was present (Fig 4E–4H). Collectively, these results indicate that the ARF domain of TRIM23 mediates physical interaction with HAdV-C5 E1A, and that both the RING domain and ARF domain of TRIM23 contribute to its antiviral effect.

To determine whether the enzymatic activity of the RING domain is essential for the autophagy function of TRIM23, we generated a TRIM23 C34A mutant, which lacks E3 ligase activity. Like the DelRING mutant, the TRIM23 C34A mutant restored the degradation of E1A (Fig 4I). We also detected polyubiquitination of endogenous TRIM23, a phenomenon that is negligible under normal conditions but increases significantly following HAdV-C5 infection (Fig 4J). This clearly indicates that the RING domain of TRIM23 is a key region that is mainly responsible for degrading the viral E1A protein to exert its antiviral function. The auto-ubiquitination of TRIM23 may play a significant role in the regulation of antiviral autophagy [26].

### TRIM23 recruits p62 as an autophagy cargo receptor for E1A degradation

**Fig 5:**
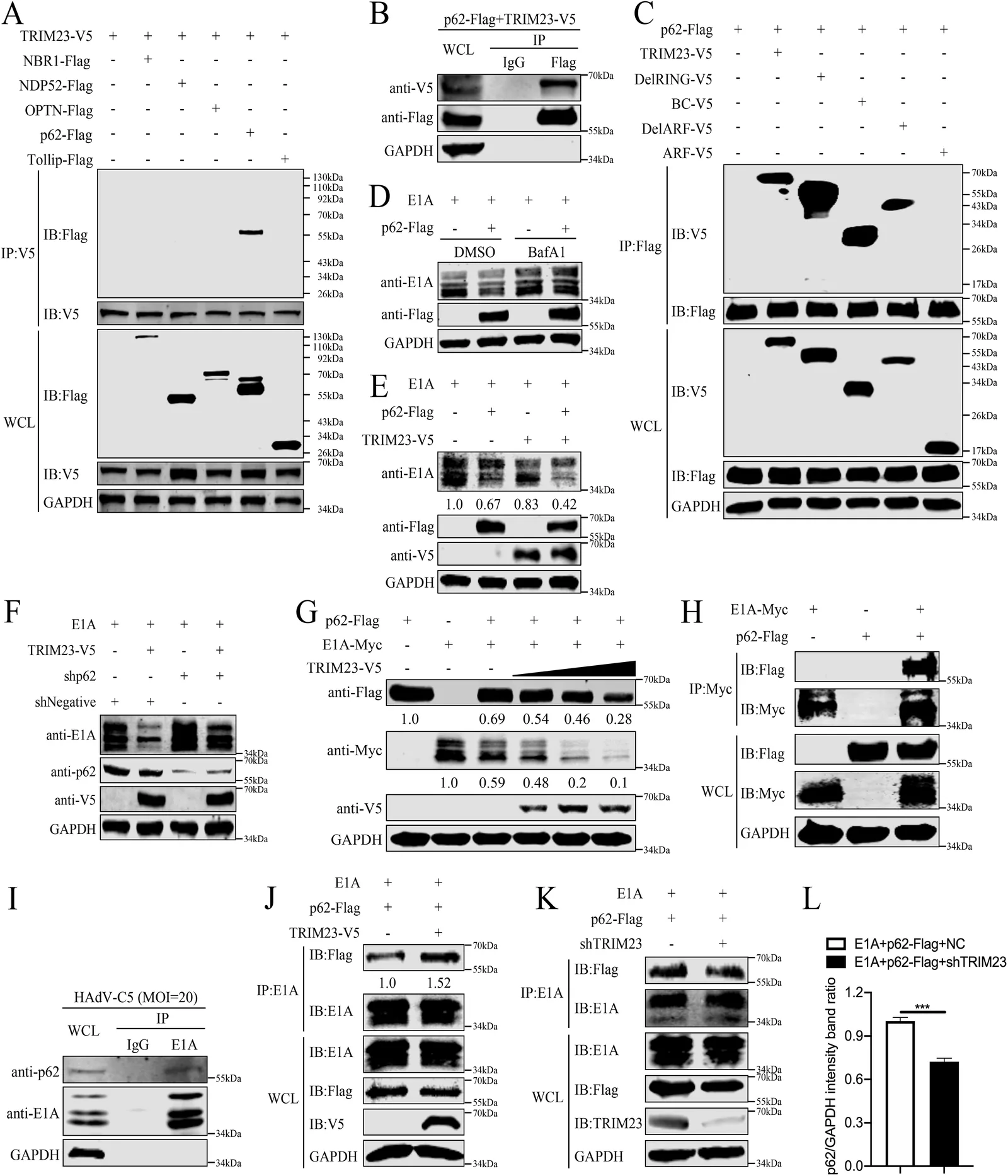
TRIM23 recruits p62 for autophagic degradation of E1A. **(A)** Flag-tagged cargo receptors (NBR1, NDP52, OPTN, Tollip, and p62) were co-transfected with V5-tagged TRIM23 into HEK293T cells for 48 hours. Co-immunoprecipitation (co-IP) was performed using an anti-V5 antibody, followed by western blot analysis. **(B)** HEK293T cells were co-transfected with Flag-tagged p62 and V5-tagged TRIM23 for 48 hours. Co-immunoprecipitation was performed using an anti-Flag antibody, followed by western blot analysis. **(C)** Co-immunoprecipitation and western blot analysis using an anti-Flag antibody after transfecting HEK293T cells with V5-tagged TRIM23 and its truncated mutants along with Flag-tagged p62. **(D)** HEK293T cells were co-transfected with Flag-p62 and E1A expression plasmid for 24 hours, then treated with BafA1 (20 mM) for 12 hours before western blot analysis. **(E)** HEK293T cells were co-transfected with E1A expression plasmid and Flag-tagged p62 expression plasmid, together with or without V5-tagged TRIM23 expression plasmid for 36 hours, followed by western blot analysis. **(F)** Immunoblot analysis of lysates of HEK293T co-transfected with E1A and shp62, together with or without V5-tagged TRIM23 for 48 **h. (G)** HEK293T cells were co-transfected with Flag-tagged p62 expression plasmid, Myc-tagged E1A expression plasmid, and V5-tagged TRIM23 expression plasmid at various concentrations for 36 hours, followed by western blot analysis. **(H)** HEK293T cells were co-transfected with Myc-tagged E1A and Flag-tagged p62 expression plasmids for 36 hours, followed by co-immunoprecipitation using an anti-Myc antibody. **(I)** A549 cells were infected with HAdV-C5 (MOI = 20) for 30 hours, followed by co-immunoprecipitation using an anti-E1A antibody and subsequent western blot analysis. **(J)** Lysates from HEK293T co-transfected with plasmids encoding E1A and Flag-p62 together with or without TRIM23-V5, and treated with BafA1 (20 mM), were subjected to co-IP assays using an anti-E1A antibody, followed by immunoblot analysis with the indicated antibodies. **(K and L)** Lysates from HEK293T co-transfected with specific shRNA targeting TRIM23 and plasmids encoding E1A and Flag-p62 were subjected to co-IP assays using an anti-E1A antibody, followed by immunoblot analysis with the indicated antibodies. Quantification of relative p62 levels is shown in **(L)**. ***, *P* < 0.001. At least three independent experiments were conducted.

Increasing evidence indicates that selective autophagy relies on cargo receptors recognizing specific substrates for lysosomal degradation [33,34]. Selective autophagy requires specific autophagy receptors to link invading proteins to autophagosomes [12], and we hypothesize that specific cargo receptors may deliver E1A to autophagosomes for degradation. Co-immunoprecipitation experiments revealed that TRIM23 interacts with p62 but does not bind to NDP52, Tollip, OPTN, or NBR1 (Fig 5A and 5B). Further, we conducted a series of immunoprecipitation to identify the interaction domain between TRIM23 and p62. The results indicate that the BC domain (B-Box and Coil-coil) of TRIM23 is the key domain involved in the interaction with p62 (Fig 5C).

To identify the molecular mechanism of p62 in autophagic degradation of E1A, the autophagy inhibitor BafA1 restored the degradation of E1A, indicating that E1A was indeed degraded through the autophagy pathway (Fig 5D). TRIM23 serves as a platform for the assembly of the autophagic machinery. Notably, we further found that TRIM23 overexpression promotes p62-mediated selective autophagic degradation of E1A (Fig 5E). TRIM23 recruits the selective autophagy receptor p62 directs E1A to phagophores for degradation. Similarly, the degradation effect of TRIM23 on E1A was significantly weakened in p62-knockdown cells, which indicated that TRIM23 mediated E1A autophagic degradation through p62 (Fig 5F). In addition, as TRIM23 levels rise, its role in degrading E1A becomes increasingly significant, leading to more p62 entering autolysosomes for degradation (Figs 5G and S4A). However, TRIM23 did not affect the ubiquitination levels of p62 (S4B–S4D Fig). Together, these results indicate that p62 plays a crucial role in the TRIM23-mediated autophagic degradation of E1A.

Since TRIM23-mediated autophagic degradation of E1A is independent of the E1A ubiquitination, we hypothesize that TRIM23 may act as an adaptor to promote the interaction between E1A and p62. To test this, we co-transfected Myc-tagged E1A and Flag-tagged p62 into A549 cells. Co-immunoprecipitation experiments confirmed that E1A also interacts with p62 (Fig 5H). Further, endogenous co-immunoprecipitation in HAdV-C5-infected A549 cells demonstrated that native E1A binds to p62 during viral infection (Fig 5I). Further analysis revealed that TRIM23 specifically enhanced the interaction between E1A and p62 (Fig 5J). In loss-of-function experiments in which TRIM23 was knocked down using shRNA, the formation of the E1A-p62 complex was impaired (Fig 5K and 5L). Our data altogether suggest that p62 functions as a cargo receptor in the TRIM23-mediated autophagic degradation of HAdV-C5 E1A.

### Construction and characterization of an oncolytic adenovirus expressing shTRIM23

**Fig 6:**
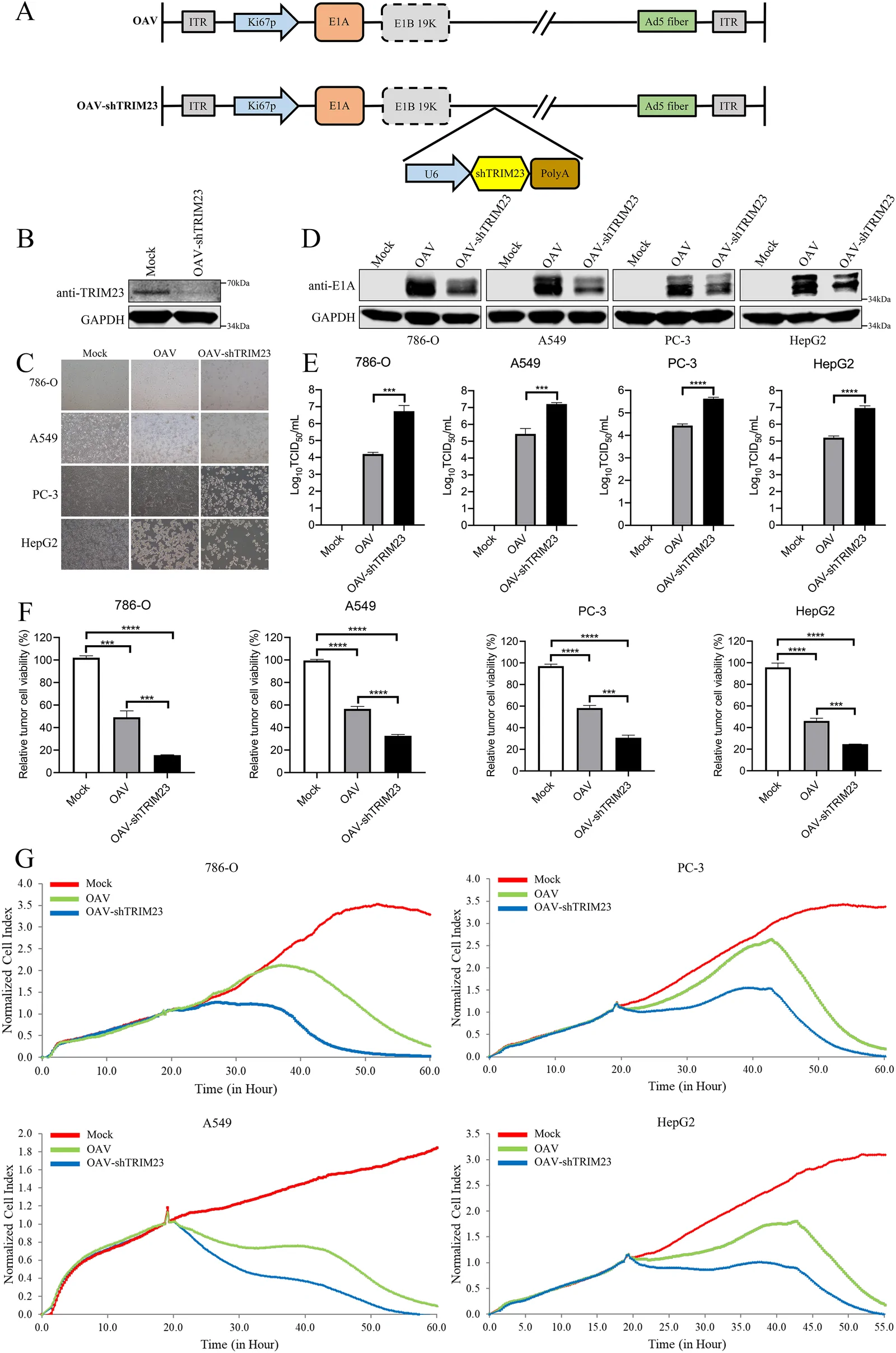
Construction of oncolytic adenovirus expressing a short hairpin RNA (shTRIM23) genome targeting TRIM23. **(A)** Schematic diagram of oncolytic adenovirus expressing the shTRIM23 genome. ITR, inverted terminal repeat; Ki67p, Ki67 promoter; E1A, adenovirus early region 1A; E1B19K, adenovirus early region 1B 19KD; Ad5 fiber, serotype 5 adenovirus (Ad5) fiber. **(B)** Detection of TRIM23 expression in A549 cells infected with OAV-shTRIM23, with uninfected A549 cells as controls. **(C, D, and E)** Infection of 786-O, A549, PC-3, and HepG2 cells with OAV or OAV-shTRIM23 at a multiplicity of infection (MOI) of 10. Tumor lysis effects were observed microscopically 36 hours post-infection. Expression of viral E1A in OAV- or OAV-shTRIM23-infected cells was detected by western blotting using an anti-E1A antibody. Subsequently, viral titers in infected cell supernatants were determined by the TCID50 assay. Scale bar, 100 μm. ***, *P* < 0.001; ****, *P* < 0.0001. **(F)** CCK-8 assay for cell viability in OAV- or OAV-shTRIM23-infected 786-O, A549, PC-3, and HepG2 cells. ***, *P* < 0.001; ****, *P* < 0.0001. **(G)** Analysis of the antiproliferative effects of OAV and OAV-shTRIM23 on 786-O, A549, PC-3, and HepG2 cells using RTCA technology. At least three independent experiments were performed.

To explore the translational potential of these findings, we constructed an oncolytic adenovirus (OAV) carrying an shRNA targeting TRIM23 (OAV-shTRIM23) (Fig 6A). To validate the successful construction of the OAV-shTRIM23 recombinant oncolytic adenovirus, we assessed TRIM23 expression levels in A549 cells infected with OAV-shTRIM23. Results demonstrated significantly reduced TRIM23 expression compared to control cells, confirming the successful construction of the OAV-shTRIM23 recombinant oncolytic adenovirus (Fig 6B). Additionally, we examined cellular changes in different cell lines infected with OAV/OAV-shTRIM23 at the same multiplicity of infection (MOI). Results showed that cells infected with OAV-shTRIM23 produced more viral particles, leading to more severe cytopathic effects (Fig 6C). Consistent with this, the levels of retained viral E1A protein in OAV-shTRIM23-infected cells were significantly lower than in conventional OAV-infected cells, as more viral particles were released into the supernatant (Fig 6D). TCID50 assays indicated that the viral titers in the supernatants of OAV-shTRIM23-infected cells were significantly higher than those in conventional OAV-infected cells (Fig 6E). Similarly, CCK-8 assays demonstrated that OAV-shTRIM23 exhibited stronger cytotoxicity than conventional OAV (Fig 6F). Real-time cell analysis (RTCA) confirmed that OAV-shTRIM23 exhibited superior antiproliferative effects across multiple tumor cell lines (Fig 6G). These data together indicated that OAV-shTRIM23 possesses stronger oncolytic activity than conventional OAVs, paving the way for further engineering and application of oncolytic adenoviruses.

### Antitumor efficacy of OAV-shTRIM23 *in vivo*

**Fig 7:**
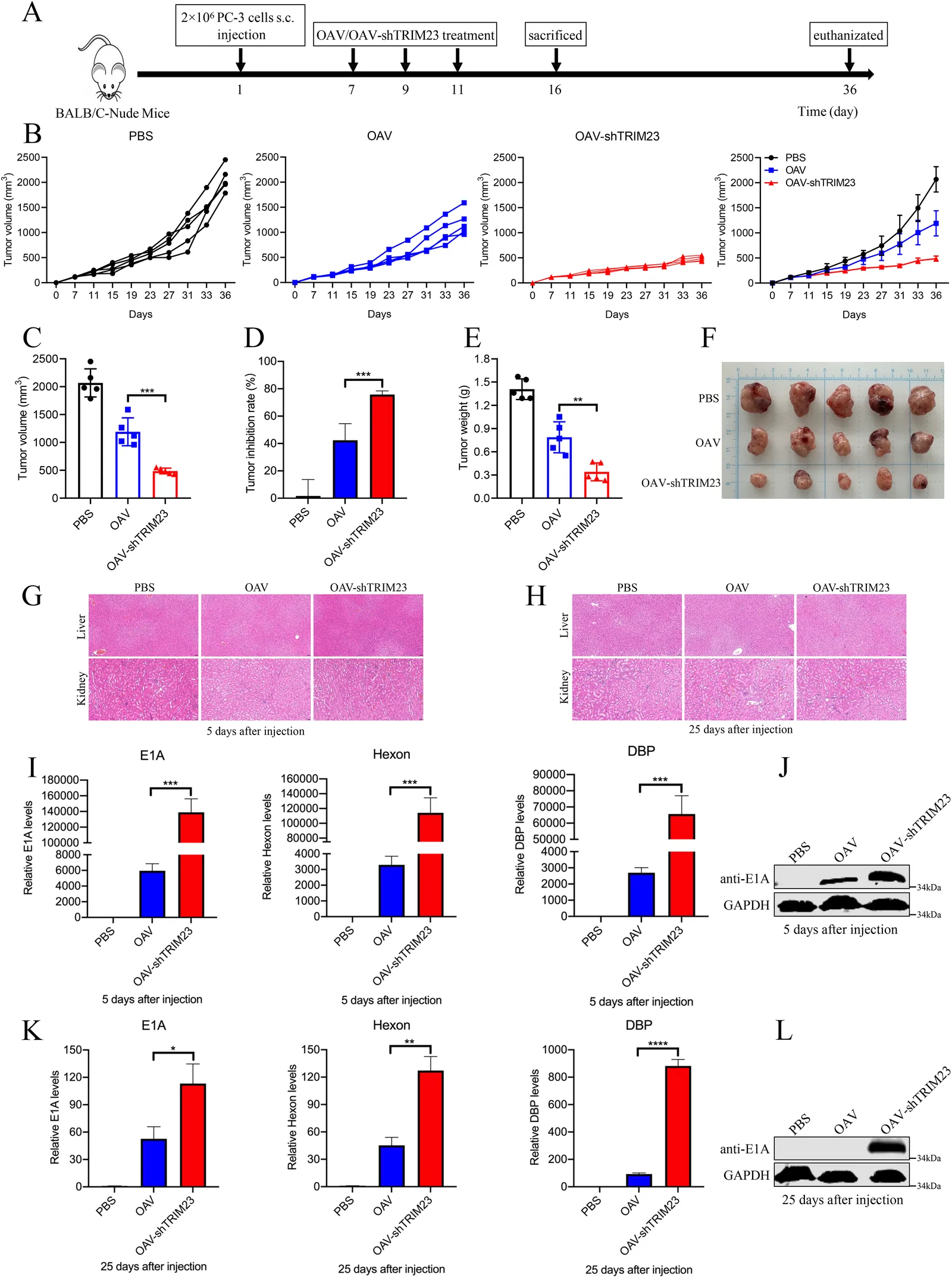
Effects of ectopic expression of shTRIM23 on *in vivo* oncolytic activity. **(A)** Schematic timeline of *in vivo* experiments. PC-3 cells (2 × 10⁶ cells per nude mouse, n = 10 per group) were subcutaneously implanted into nude mice. When tumor volume reached 50-100 mm³, tumors received three injections every other day with PBS, OAV, or OAV-shTRIM23 (1 × 10⁹ PFU). **(B)** Tumor growth curves for each mouse. **(C)** Mean tumor volume curves ± standard deviation. ***, *P* < 0.001. **(D)** Tumor inhibition rates for each group. ***, *P* < 0.001. **(E)** Tumor weights for each group. **, *P* < 0.01. **(F)** Tumor volumes for each group. **(G and H)** Effects of OAV/OAV-shTRIM23 on liver and kidney toxicity in mice assessed by HE stains on day 5 and day 25 post-injection. Scale bar, 50 μm. **(I, J, K, and L)** Detection of E1A, Hexon, and DBP viral gene expression in tumor tissues on days 5 and 25 after three treatments with OAV/OAV-shTRIM23. Subsequently, E1A viral protein expression in tumors was detected by western blot. *, *P* < 0.05; **, *P* < 0.01; ***, *P* < 0.001; ****, *P* < 0.0001.

We next evaluated the antitumor activity of OAV-shTRIM23 in a PC-3 xenograft mouse model. Mice bearing established tumors were intratumorally injected with PBS, OAV, or OAV-shTRIM23 (Fig 7A). OAV-shTRIM23 treatment significantly suppressed tumor growth compared to PBS or OAV alone, achieving 75.78% inhibition in the PC-3 model (Fig 7B–7F), without apparent toxicity to liver or kidney tissues (Fig 7G and 7H). Additionally, on day 5 or day 25 post-administration, three mice per group were euthanized, and genomic DNA was extracted from tumor tissues. Real-time PCR data indicated that tumors treated with OAV-shTRIM23 exhibited higher viral genome copies compared to OAVs (Fig 7I and 7K). Increased E1A protein was also detected in OAV-shTRIM23-treated tissues (Fig 7J and 7L). The data indicate that OAV-shTRIM23 produces a significant antitumor effect against prostate cancer *in vivo*.

## Discussion

Members of the TRIM E3 ligase family are well-established regulators of innate immune surveillance [35]. Growing evidence further indicates that several TRIM proteins modulate autophagy in response to viral infection or other cellular stresses [36]. EXOC5 facilitates the autophagic degradation of STING via K63-linked polyubiquitination at Lys224 and Lys338 by the E3 ligase TRIM56, which serves as a recognition signal for the cargo receptor SQSTM1/p62 [37]. TRIM25 activates the RIG-I sensor through K63-polyubiquitination, specifically regulating the autophagic response to influenza viruses [38,39]. Furthermore, TRIM13 regulates EMCV-induced interferon production via the MDA5 sensor while being critical for EMCV-triggered autophagy [40]. These associations suggest complex interplay between TRIM proteins’ functional roles in innate signaling against specific viral pathogens and autophagy processes.

**Fig 8:**
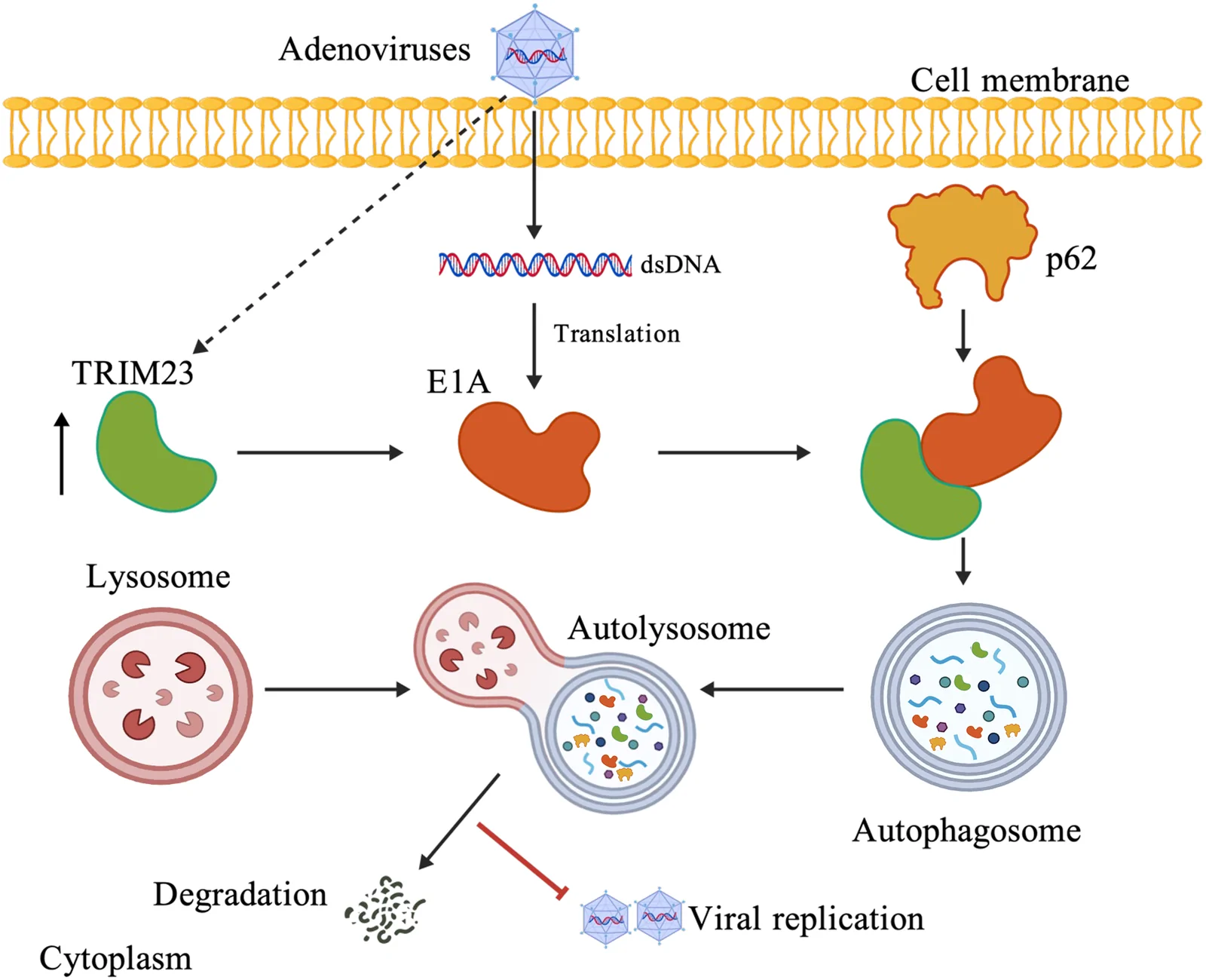
Working model of the TRIM23-E1A-p62 axis. TRIM23 binds to HAdV-C5 E1A and recruits the selective autophagy receptor p62, leading to autophagic degradation of E1A and inhibition of viral replication.

Autophagy plays a complex role in viral infections, sometimes promoting and other times restricting viral replication. While previous studies have suggested that HAdV-C5 may exploit autophagy to facilitate its replication, the precise mechanisms remain incompletely understood [41,42]. Here, we identify TRIM23 as a novel host factor that restricts HAdV-C5 replication by targeting the viral E1A for autophagic degradation. We demonstrate that TRIM23 acts as an adaptor, recruiting the selective autophagy receptor p62 to direct E1A to phagophores for lysosomal degradation. This TRIM23-p62-E1A axis represents, to our knowledge, the first evidence implicating TRIM23 in antiviral defense against adenovirus (Fig 8). TRIM23 is a new substrate of TBK1, and TBK1 is an important autophagy regulator recently discovered [43–47]. During HSV-1 infection, the key to TRIM23 activation is TBK1-mediated phosphorylation of the RING domain (S39), which acts as an ‘on/off’ switch for its E3 ligase activity [25]. As TRIM23 mediates the response of autophagy to multiple viruses [26], it is interesting to explore whether TBK1 regulates TRIM23 activation during HAdV-C5 infection, and if so, which PRR(s) drive TRIM23-TBK1-mediated autophagy in these cases. During infection with a specific virus, multiple different PRRs are activated in a time- and or cell-type-specific manner. However, the innate receptors and upstream pathways that specifically activate TBK1-mediated autophagy during viral infection are still unclear.

We found that HAdV-C5 infection upregulates TRIM23 expression, which in turn suppresses viral replication. Conversely, TRIM23 knockdown enhanced viral propagation, confirming its role as a bona fide restriction factor. Mechanistically, TRIM23 interacts with E1A via its C-terminal ARF domain and promotes E1A degradation through autophagy rather than via the ubiquitin-proteasome system. Interestingly, TRIM23-mediated degradation of E1A occurred without altering its ubiquitination status. Furthermore, given that TRIM23 exerts both positive and negative regulatory roles in the antiviral type I interferon response [48,49], further investigation is required to elucidate the molecular mechanisms by which TRIM23 dual-coordinates virus-induced autophagy and the interferon response. Given recent evidence that TRIM proteins function as specific autophagy receptors by recognizing viral components [24,50], future studies should elucidate how TRIM proteins establish molecular links between innate immune recognition and autophagy, as well as which viral (or host) features TRIM proteins recognize to trigger the autophagy process.

Our work adds to emerging models in which selective autophagy receptors contribute to antiviral immunity by targeting viral components for degradation. Examples include galectin 9, which directs HBV core protein to autophagosomes via p62 [51], and BST2/tetherin, which promotes autophagic clearance of porcine epidemic diarrhea virus nucleocapsid protein via NDP52 [52]. In this context, we identify p62 as the key receptor in TRIM23-mediated clearance of E1A. Notably, TRIM23 facilitated the physical interaction between E1A and p62, thereby coupling viral cargo to the autophagic machinery.

Adenoviruses (Ads) can be genetically engineered to selectively replicate within cancer cells and induce tumor lysis [53,54]. The advantage of viral therapy lies in the fact that the released progeny viruses continue to spread and infect until all cancer cells are eliminated [28]. Adenoviruses with the E1b gene deleted can selectively replicate within cancer cells and trigger tumor lysis [55]. Oncolytic adenoviruses represent a promising therapeutic strategy, but their clinical efficacy has often been limited by suboptimal viral replication and spread within tumors [56–58]. We reasoned that modulating host restriction factors could enhance viral oncolysis. By constructing an oncolytic adenovirus expressing shRNA against TRIM23 (OAV-shTRIM23), we achieved higher viral replication, increased cytotoxicity *in vitro*, and superior antitumor efficacy in a xenograft model. These findings suggest that targeting TRIM23-or similar host checkpoint proteins-could potentiate oncolytic virotherapy.

Several important questions remain. First, while TRIM23 promotes autophagic degradation of E1A, its potential role in modulating other stages of the viral life cycle-especially in viruses known to co-opt autophagy-warrants further investigation. Second, given reported dual roles of TRIM23 in type I interferon responses, future studies should elucidate how TRIM23 coordinates autophagy with innate immune signaling during infection. Finally, whether other TRIM proteins function as autophagy adaptors for viral antigens, and what structural features determine cargo specificity, are exciting avenues for further research.

In summary, this study defines a novel antiviral mechanism in which TRIM23 restricts HAdV-C5 by mediating autophagic clearance of E1A via p62. Our findings not only advance the understanding of TRIM protein functions in host defense but also highlight the therapeutic potential of manipulating selective autophagy to enhance oncolytic virotherapy.

## Supporting information

S1 Rawgel Raw data of western blot. (PDF)

S1 Fig The effect of TRIM23 on cell viability. **(A)** CCK-8 assay to evaluate A549 cell viability upon TRIM23 overexpression. **(B)** CCK-8 assays measured cell viability in TRIM23-knockdown A549 cells. **(C)** Cytopathic effects in infected cells were observed microscopically after TRIM23 knockdown. Scale bar, 100 µm. At least three independent experiments were performed.
(TIF)

S2 Fig TRIM23 downregulates the transcription of HAdV-C5 viral genes. **(A, B, C, and D)** A549 cells stably overexpressing TRIM23 and control cells were infected with HAdV-C5 at an MOI of 5. Twenty-four- or forty-eight-hours post-infection, viral RNA (E1A, Hexon, and DBP) and TRIM23 mRNA levels were measured by RT-qPCR. **(E, F, G, and H)** A549 cells were transfected with siRNA targeting TRIM23 or control siRNA, then infected with HAdV-C5 at an MOI of 5 for 24 or 48 hours. Viral RNA (E1A, Hexon, and DBP) and TRIM23 mRNA levels were measured by RT-qPCR. *, *P* < 0.05; **, *P* < 0.01; ***, *P* < 0.001; ****, *P* < 0.0001. At least three independent experiments were performed.
(TIF)

S3 Fig Detection of TRIM23 expression levels. **(A and B)** Endogenous TRIM23 mRNA levels and E1A mRNA levels were detected by RT-qPCR analysis in A549 cells at 0, 6-, 9-, 12-, 24-, and 36-hours post-infection with HAdV-C5 at a multiplicity of infection (MOI) of 20. *, *P* < 0.05; ***, *P* < 0.001; ****, *P* < 0.0001. **(C)** Relative transcriptional levels of TRIM23 were determined in HEK293T cells treated with a control, or stimulated with 10, 100, and 1,000 U/mL IFNα for 18 hours, using RT-qPCR analysis. The results are expressed as the fold change relative to cells treated with the control. **(D)** Protein abundance of endogenous TRIM23 in HEK293T cells treated with a control, or stimulated with 10, 100, and 1,000 U/mL IFNα for 18 hours, using western blot analysis. **(E)** V5-tagged TRIM23 were co-transfected with increased E1A into HEK293T cells for 48 hours, followed by western blot analysis. At least three independent experiments were performed.
(TIF)

S4 Fig TRIM23 degrades p62. **(A)** HEK293T cells were co-transfected with Flag-tagged p62 plasmid and V5-tagged TRIM23 plasmid at various concentrations, followed by western blot analysis using specific antibodies. At least three independent experiments were conducted. **(B, C, and D)** HEK293T cells were cotransfected with Flag-tagged p62 and pHA-Ub WT (B), pHA-Ub K48 (C), or pHA-Ub K63 (D), together with or without V5-tagged TRIM23, separately. Ubiquitination assays were performed and blotted with indicated antibodies. At least three independent experiments were performed.
(TIF)

S1 Table Analysis of differential results of E1A proteomics. (XLSX)
