# Supplementary material for: TRIM23 prevents adenovirus replication by p62-mediated selective autophagic degradation of viral E1A protein
Source: PLoS Pathog. 2026 Sep 8;22(9):e1014578. doi: 10.1371/journal.ppat.1014578 (PMC13577515; doi:10.1371/journal.ppat.1014578)

Fig.1D

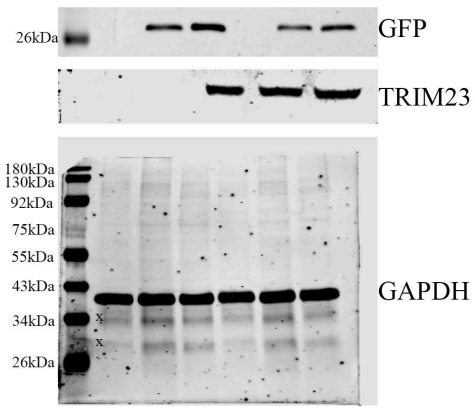

Fig.1F

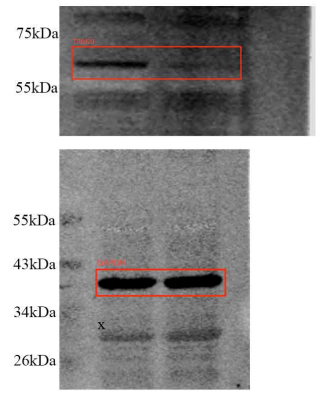

Fig.1H

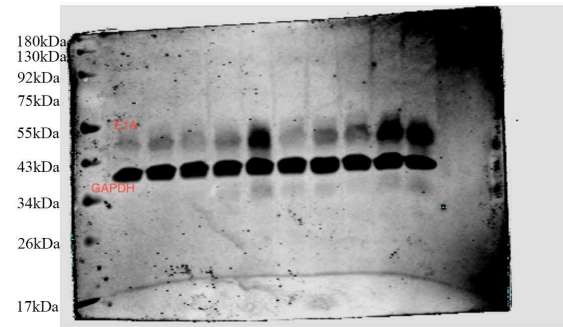

Fig.2G

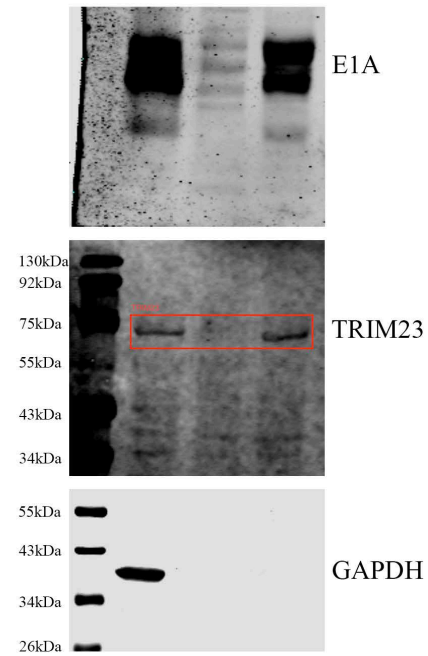

Fig.2A

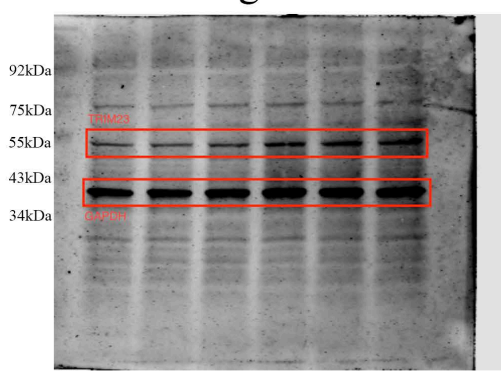

Fig.2E

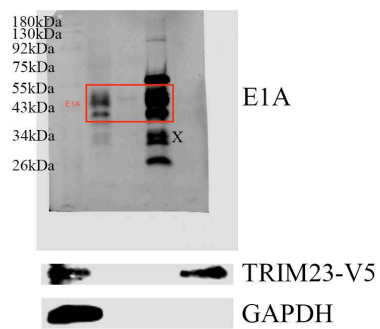

Fig.2F

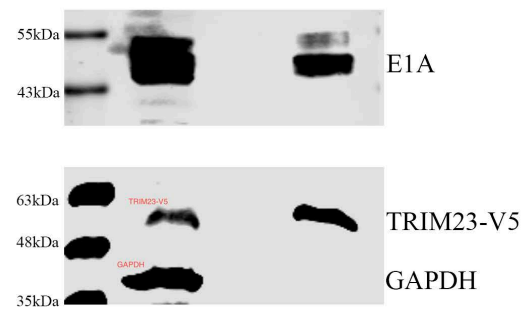

Fig.2H

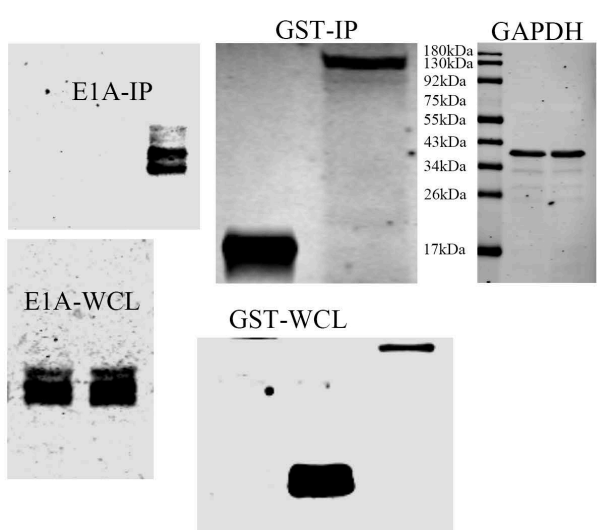

Fig.2J

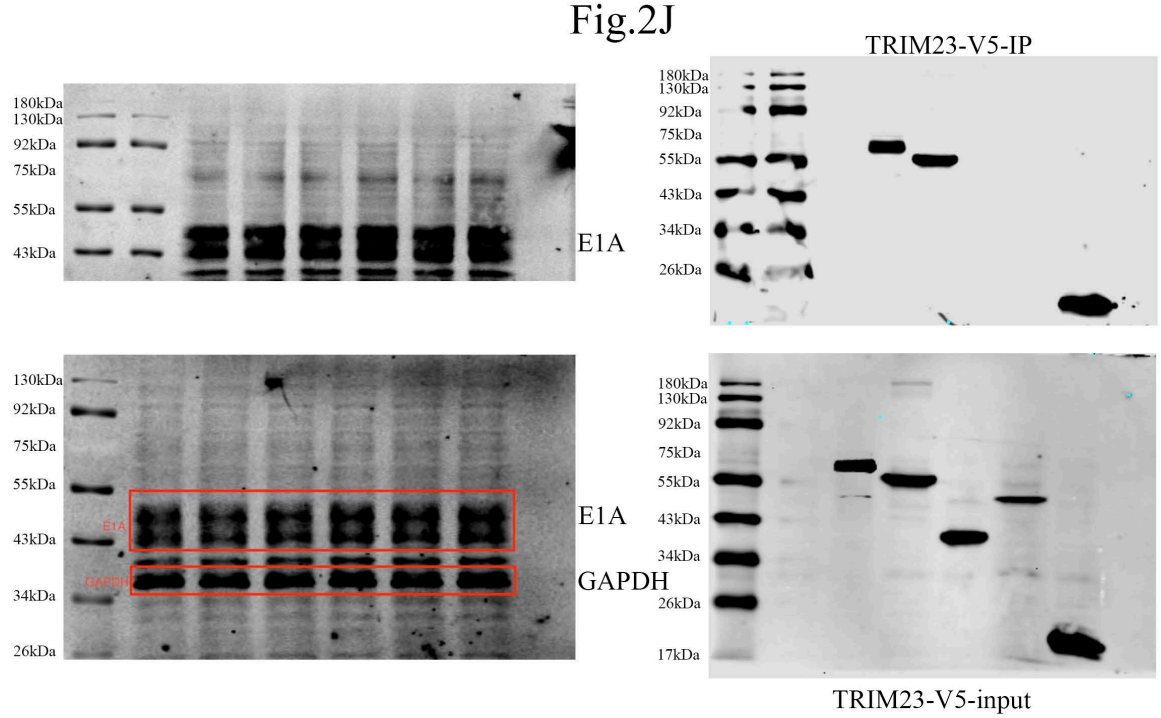

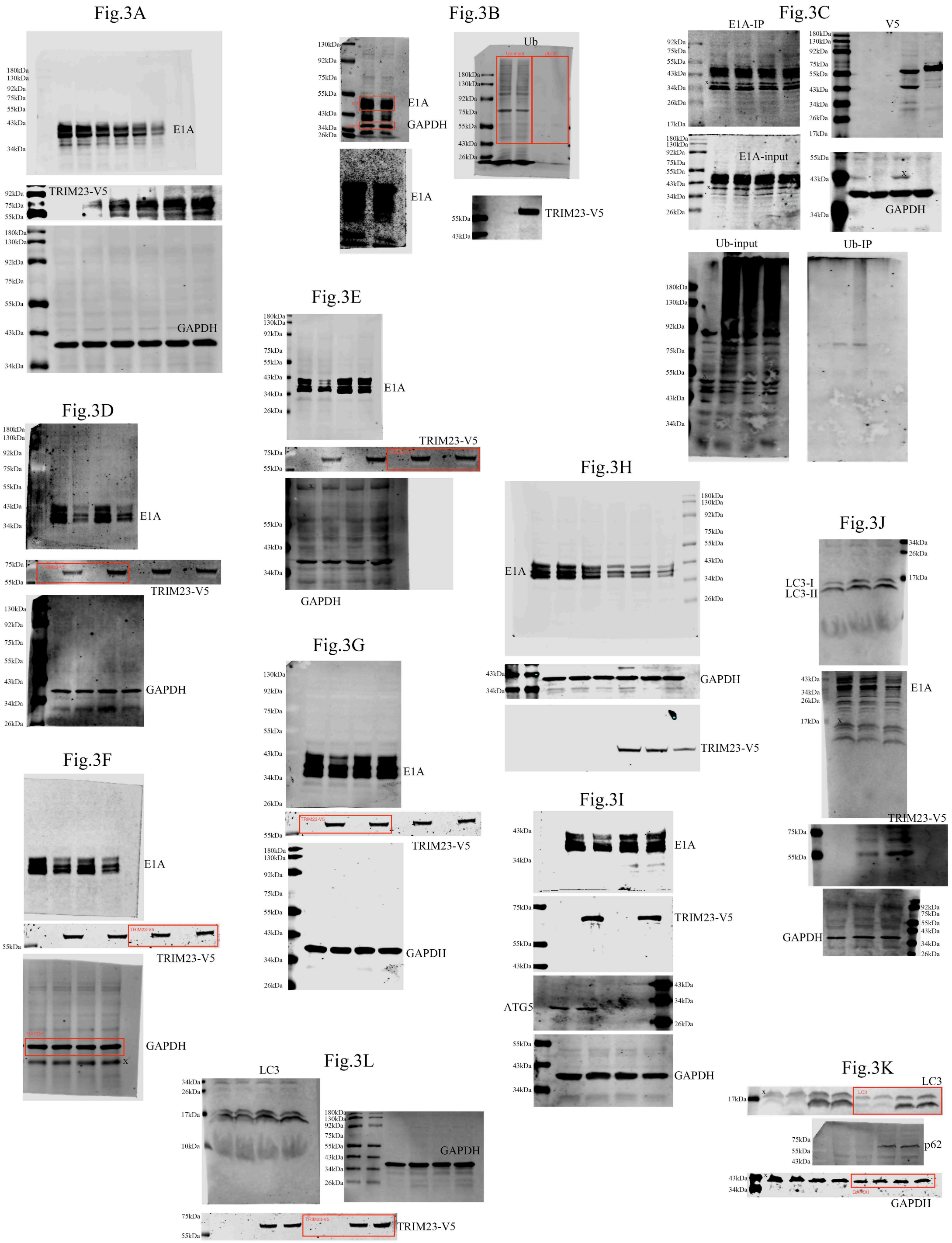
Fig.3A
E1A
TRIM23-V5
GAPDH
Fig.3B
E1A
GAPDH
Ub
TRIM23-V5
Fig.3C
E1A-IP
V5
E1A-input
GAPDH
Ub-input
Ub-IP
Fig.3D
E1A
TRIM23-V5
GAPDH
Fig.3E
E1A
TRIM23-V5
GAPDH
Fig.3F
E1A
TRIM23-V5
GAPDH
Fig.3G
E1A
TRIM23-V5
GAPDH
Fig.3H
E1A
GAPDH
TRIM23-V5
Fig.3I
E1A
TRIM23-V5
ATG5
GAPDH
Fig.3J
LC3-I
LC3-II
E1A
TRIM23-V5
GAPDH
Fig.3K
LC3
p62
GAPDH
Fig.3L
LC3
GAPDH
TRIM23-V5

Fig.4C

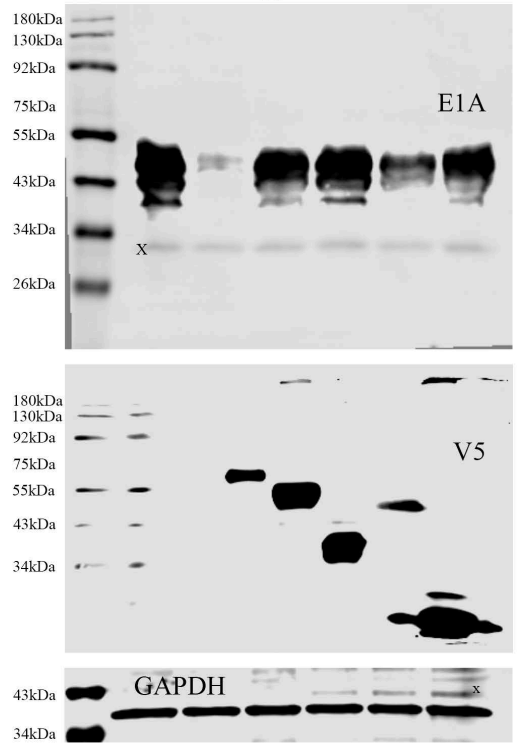

Fig.4D

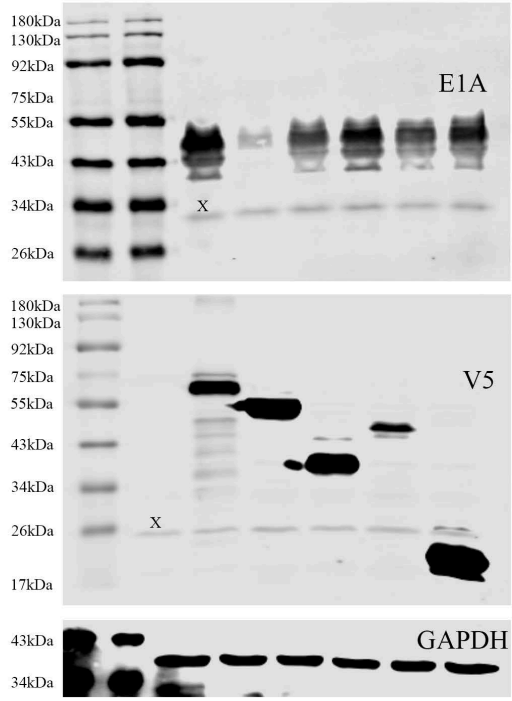

Fig.4E

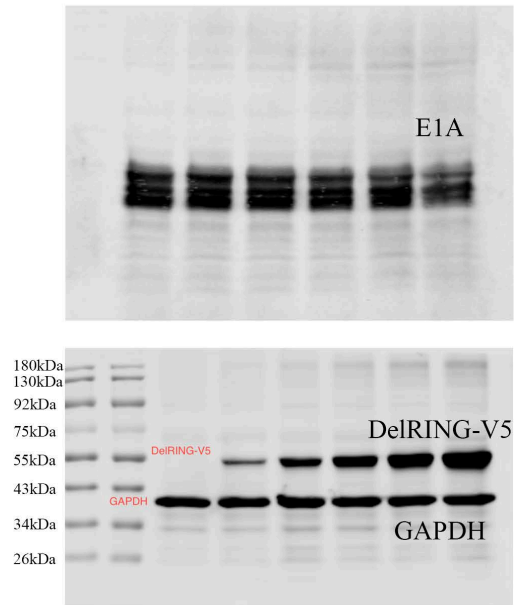

Fig.4H

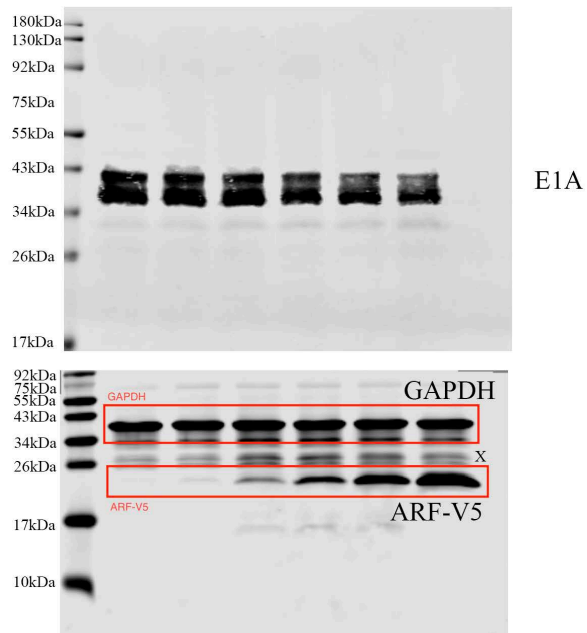

Fig.4F

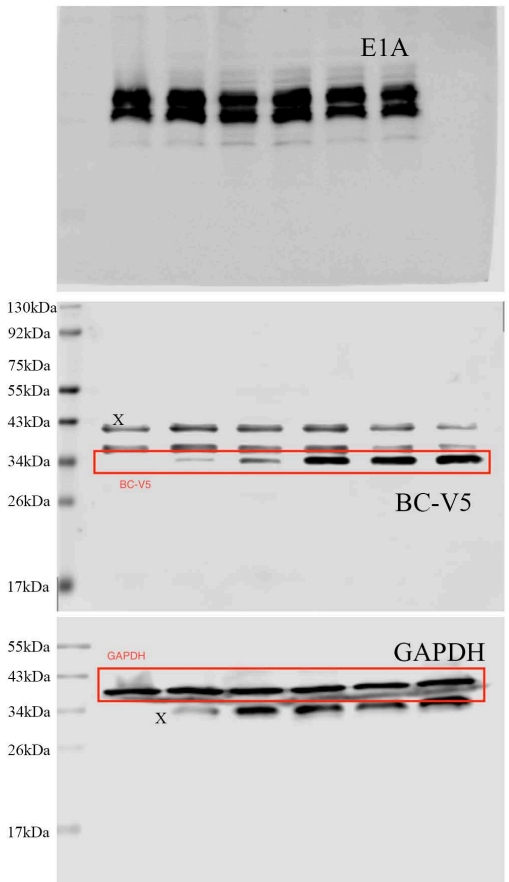

Fig.4G

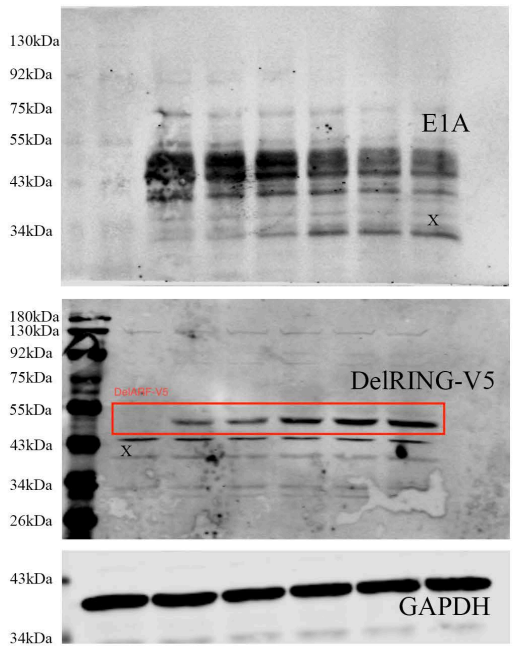

Fig.4I

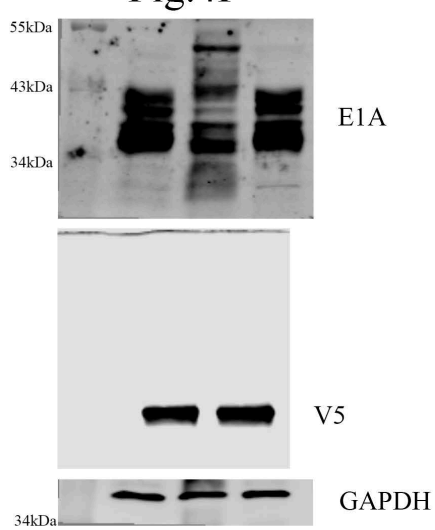

Fig.4J

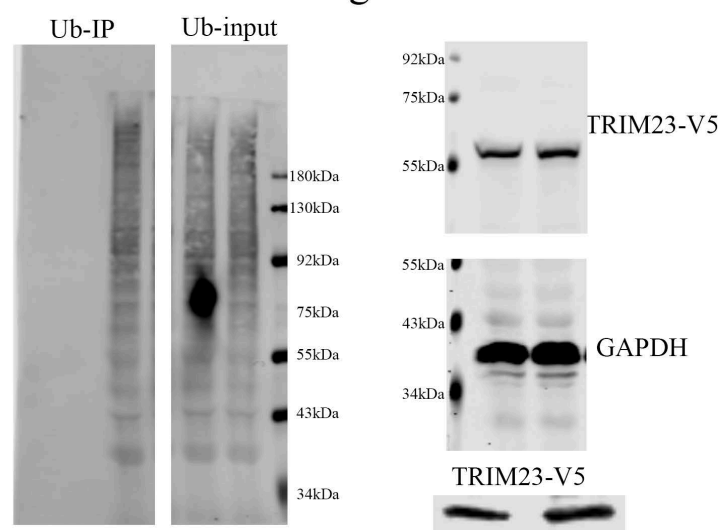

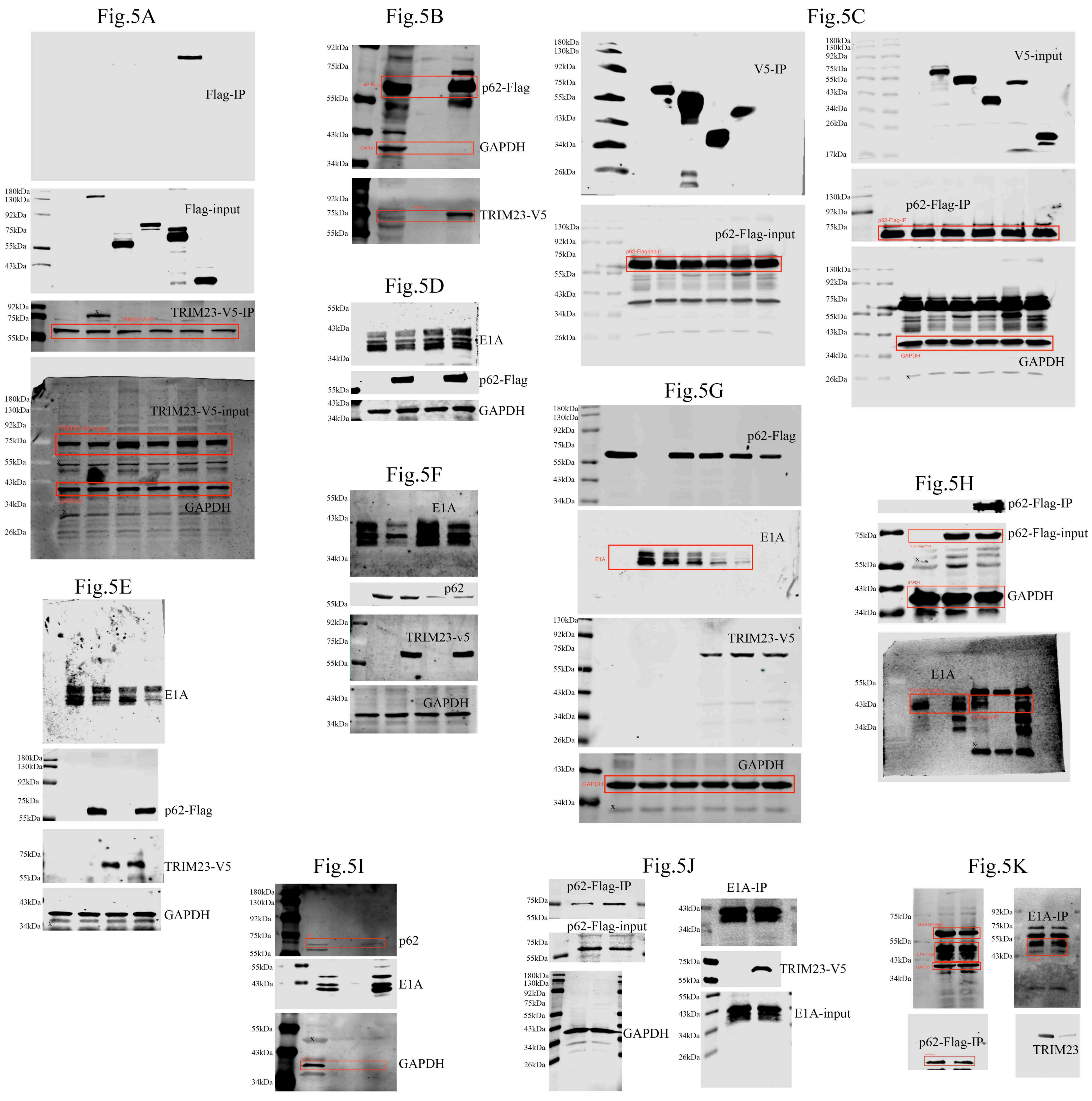
Fig.5A
Flag-IP
Flag-input
TRIM23-V5-IP
TRIM23-V5-input
GAPDH
Fig.5B
p62-Flag
GAPDH
TRIM23-V5
Fig.5C
V5-IP
V5-input
p62-Flag-input
p62-Flag-IP
GAPDH
Fig.5D
E1A
p62-Flag
GAPDH
Fig.5E
E1A
p62-Flag
TRIM23-V5
GAPDH
Fig.5F
E1A
p62
TRIM23-v5
GAPDH
Fig.5G
p62-Flag
E1A
TRIM23-V5
GAPDH
Fig.5H
p62-Flag-IP
p62-Flag-input
GAPDH
E1A
Fig.5I
p62
E1A
GAPDH
Fig.5J
p62-Flag-IP
p62-Flag-input
GAPDH
E1A-IP
TRIM23-V5
E1A-input
Fig.5K
E1A-IP
p62-Flag-IP
TRIM23

Fig.6B

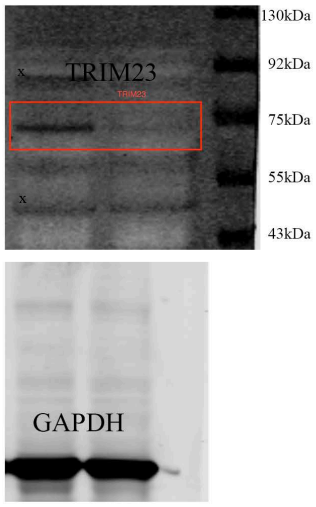

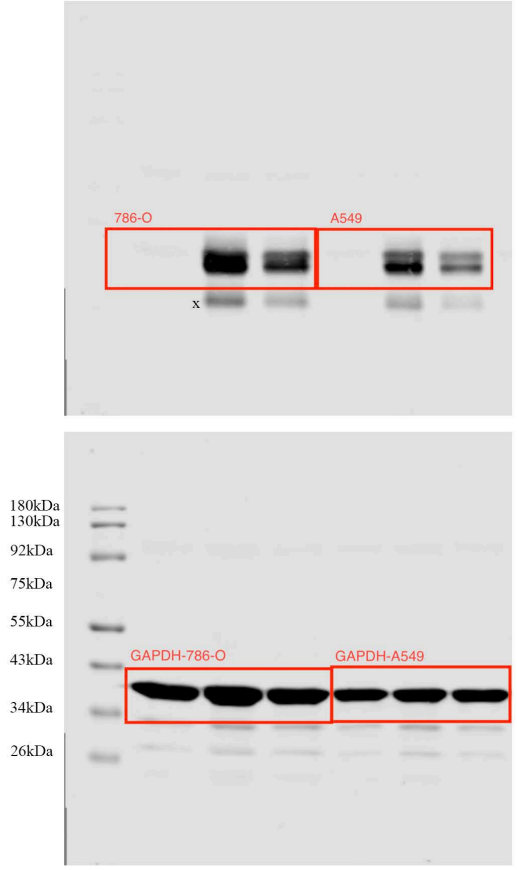

Fig.6D

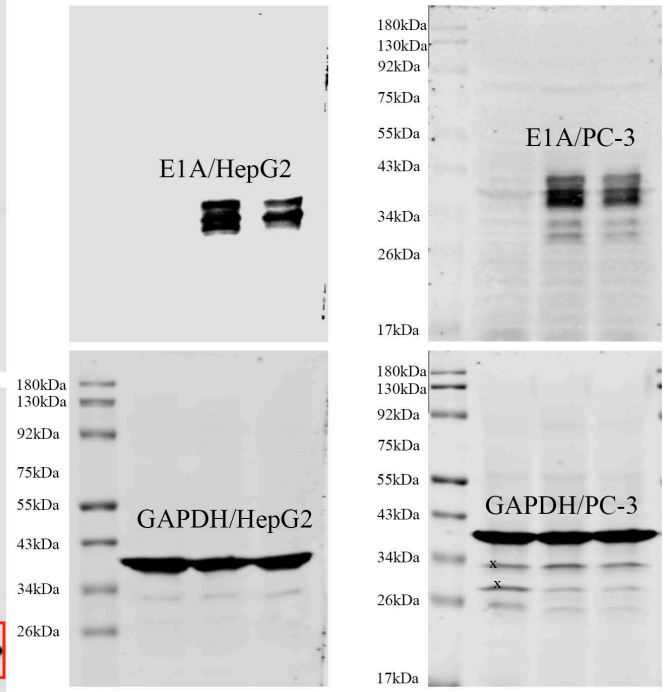

Fig.S3D

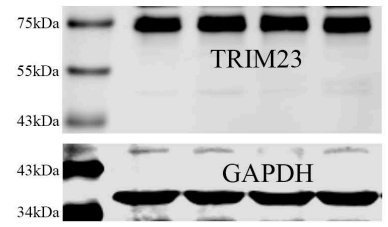

Fig.7J

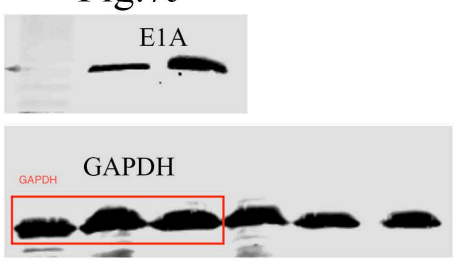

Fig.S3E

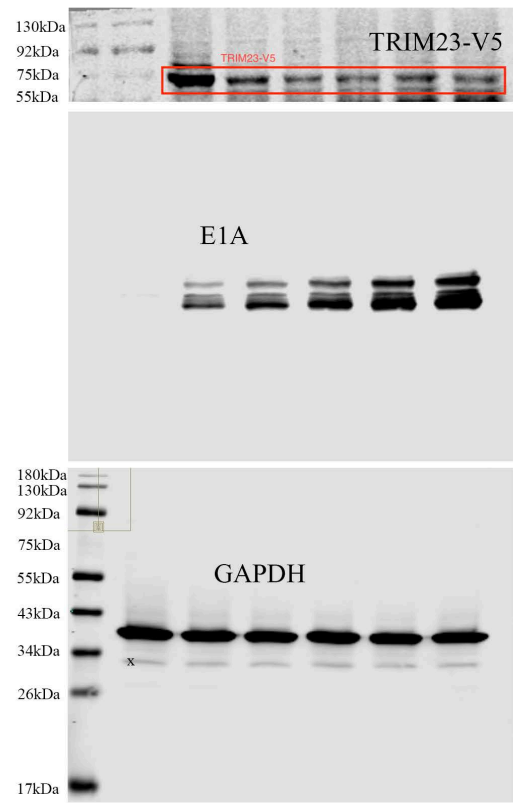

Fig.7L

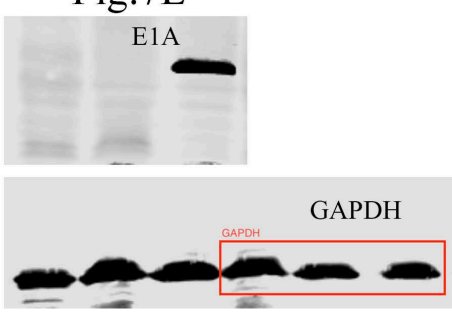

Fig.S4A

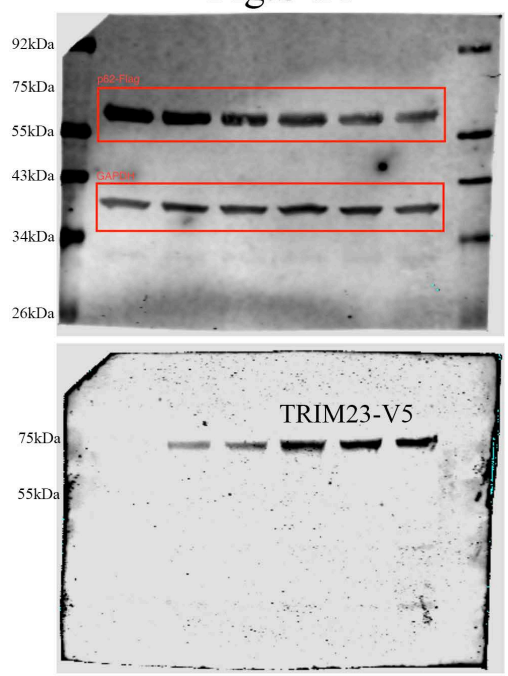

Fig.S4C

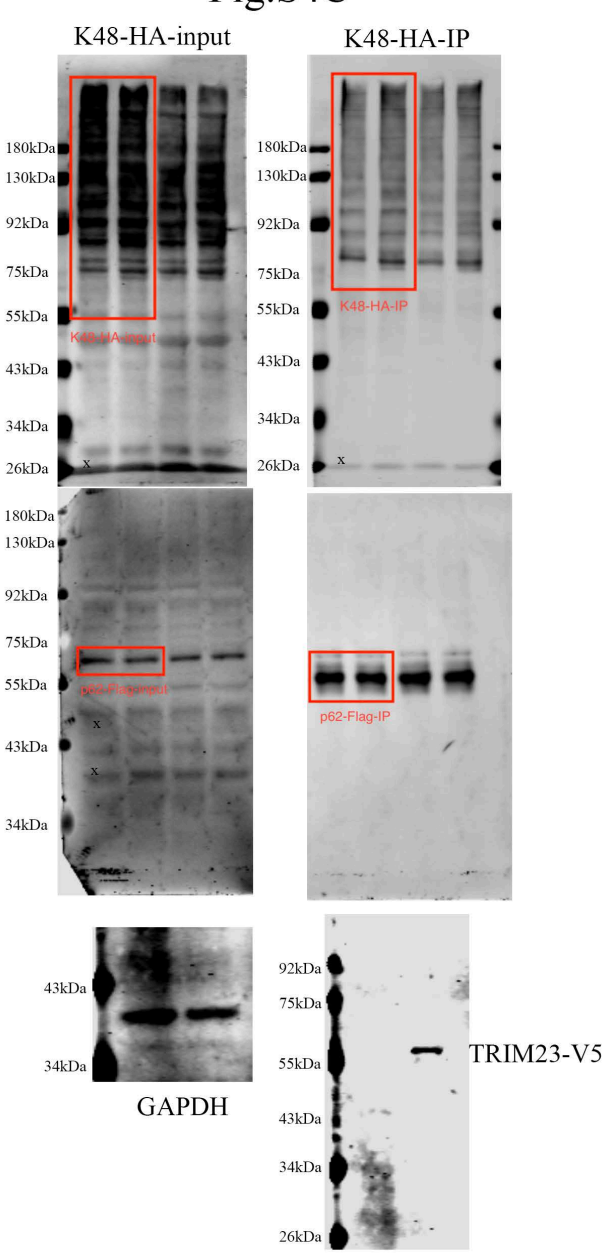

Fig.S4D

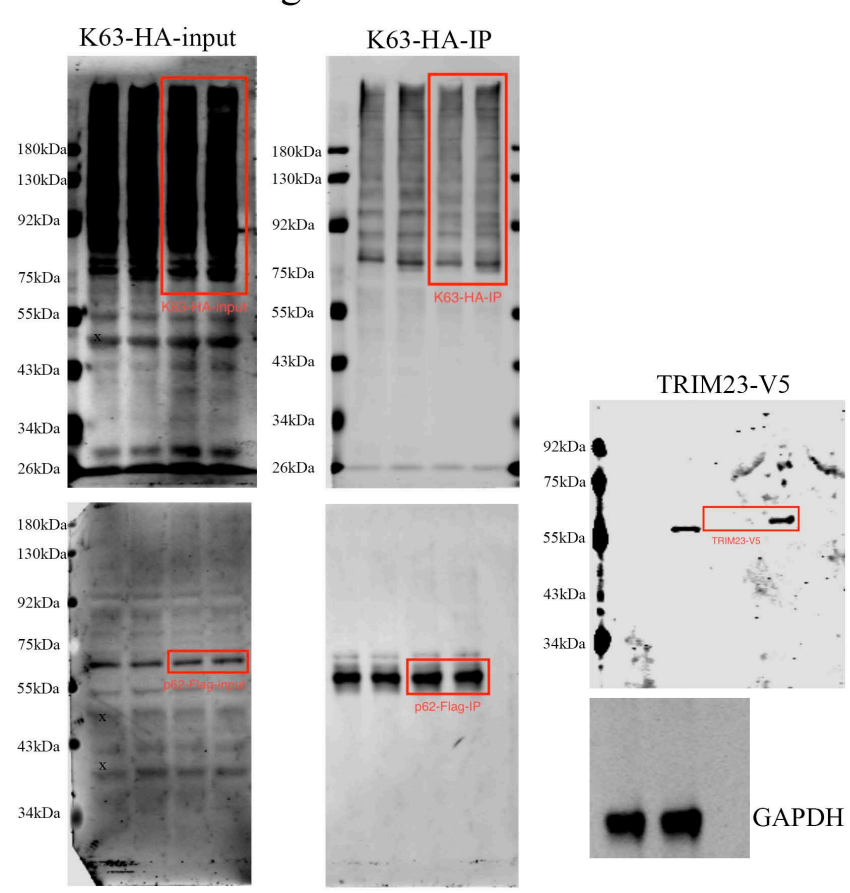

Fig.S4B

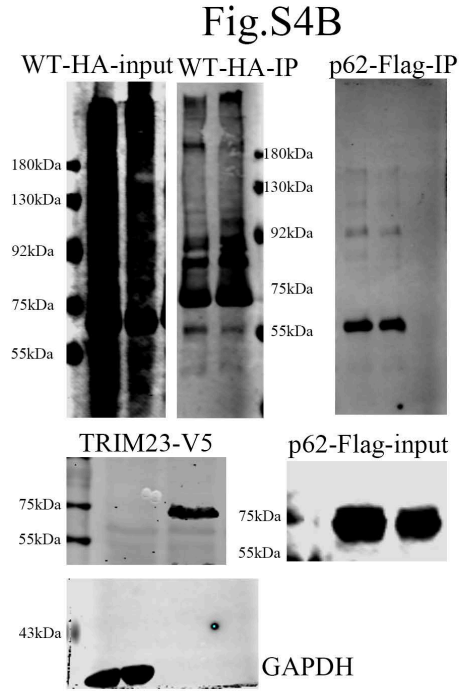

Supplement: Supplementary file 1 [file ppat.1014578.s001.pdf]
