# Supplementary material for: TRIM23 prevents adenovirus replication by p62-mediated selective autophagic degradation of viral E1A protein
Source: PLoS Pathog. 2026 Sep 8;22(9):e1014578. doi: 10.1371/journal.ppat.1014578 (PMC13577515; doi:10.1371/journal.ppat.1014578)
Supplement: Supplementary file 2 [file ppat.1014578.s007.docx]

The following are our responses to the comments (*in Italics*) of the reviewers and the changes made in this manuscript (**PPATHOGENS-D-26-00171**).

**Reviewers’ Comments:**

Reviewer’s Responses to Questions

**Part I - Summary**
Please use this section to discuss strengths/weaknesses of study, novelty/significance, general execution and scholarship.

*[****Reviewer #1:*** *Tripartite motif-containing protein 23 (TRIM23) is a unique protein that possesses RING E3 ligase and ARF GTPase activities. TRIM23 has been linked to various cellular processes and has been previously recognized as an important regulator of autophagy during viral infections (mainly herpesvirus but also adenovirus, e.g. PMID: 28871090). In this manuscript submitted to PLOS Pathogens, the authors report that human adenovirus type 5 (HAdV-C5) upregulates TRIM23 in A549 cells. By utilizing multiple approaches, the authors convincingly demonstrate that TRIM23 potently restricts HAdV-C5, which is consistent with a previous report (Sparrer et al., 2017). The authors show that TRIM23 interacts with early protein 1A (E1A) of HAdV-C5 and degrades this protein in an autophagy-dependent manner. The authors propose that TRIM23’s E3 ligase activity is indispensable for E1A degradation as no ubiquitination of E1A was detected. Finally, the authors generated an oncolytic adenovirus (OAV) carrying an shRNA targeting TRIM23 (OAV-shTRIM23) and showed that OAV-shTRIM23 has higher replication fitness and possesses stronger antiproliferative/cytopathic activity in cells, which was also demonstrated in a xenograft mouse model of prostate cancer.

Strengths of the study are the data showing an important role of TRIM23 in HAdV-C5 restriction via autophagy as well as the data showing a higher replication fitness for OAV-shTRIM23 that led to enhanced tumor suppressing ability. However, the data on the proposed molecular mechanism for TRIM23-mediated AdV restriction via E1A mechanism are unconvincing, and some of the experimental designs have flaws. Taken together, despite the novelty of the OAV-shTRIM32 studies and their translational value, there are several experimental issues in the mechanistic studies (see specific comments below) that decreased the overall quality of the manuscript.]*

We thank the reviewer for summarizing our work. We also appreciate the reviewer’s very professional and insightful suggestion, which enables us to significantly improve the present work!

General major comments:

*[1. The conclusions that “TRIM23 promotes E1A degradation through a mechanism independent of its canonical E3 ligase activity” and “TRIM23 acts as an adaptor for p62-E1A interaction” are not convincingly supported by the data. This part of the manuscript requires extensive additional experimentation.]*
**R:** Thanks for your comments and questions. As your suggestion, we supplement a series of experiments to verify our conclusions, and the results are shown in detail in the second part.

*[2. The authors show that TRIM23, via its autophagy activity, degrades E1A. However, the proposed mechanism clashes with the authors’ own functional data (i.e., TRIM23 truncation proteins that are autophagy defective can still restrict HAdV-C5) as well as with the previously published mechanism for TRIM23-mediated autophagy where its RING E3 ligase activates via auto-K27-linked ubiquitination the ARF GTPase activity, which is important for relocalizing TRIM23 onto autophagosomes where it binds to and triggers TBK1-mediated p62 phosphorylation, ultimately inducing antiviral autophagy (in herpesvirus infection). It is likely that the same mechanism of TRIM23-mediated autophagy induction may also apply to HAdV infection.]*
**R:** Thanks for your comments and questions. As your suggestion, we supplement a series of experiments to verify our conclusions, and the results are shown in detail in the second part.

*[3. There are substantial flaws and inconsistencies in the experiment design and data interpretation (see specific comments below).]*

**R:** Thanks for your comments and questions. We will carefully supplement a series of experimental data to improve our work.

*[****Reviewer #2:*** *Sun et al have identified Trim23 as a new adenovirus E1A interacting protein. Trim23 overexpression inhibits HAdV-C5 early gene transcription, early protein expression, genome replication and infectious progeny production. All of these effects seem to arise from Trim23 leading to E1A degradation via autophagy.

Overall, this is an interesting paper with some high-quality data. There is a lot of very solid data in this manuscript and the conclusion mostly seem well justified. However, many critical details are missing and there are a number of minor problems with the language.]*

We thank the reviewer for summarizing our work and stating that “Overall, this is an interesting paper with some high-quality data. There is a lot of very solid data in this manuscript and the conclusion mostly seem well justified”. We also appreciate the reviewer’s very professional and insightful suggestion, which enables us to significantly improve the present work! In addition, we have professionally modified and polished the grammatical sentences of the full text. After addressing the issues raised by the reviewer, we do think the current manuscript could be considered for publication in the PLoS Pathogens.

*[****Reviewer #3:*** *The title of this review is “TRIM23 prevents adenovirus replication by p62-mediated selective autophagic degradation of viral E1A protein”.

The manuscript investigates the antiviral role of TRIM23 against human adenovirus type 5 (HAdV-C5), proposing that TRIM23 interacts with the viral protein E1A, promotes its degradation via an autophagosome-dependent mechanism, and suppresses transcription of HAdV-C5 viral genes. While the study presents potentially interesting findings, there is a fundamental conceptual issue that significantly weakens the overall premise of the work.
The entire story is built upon the initial observation that TRIM23 is upregulated in E1A-overexpressing cells (first result part; through proteomic analysis….). This finding serves as the primary rationale and conceptual starting point of the manuscript. In other words, the authors justify the investigation of TRIM23 as an antiviral factor based on its induction by E1A. However, this central rationale raises a critical and unresolved paradox.
According to the authors’ model, TRIM23 promotes degradation of E1A and suppresses viral gene transcription, thereby inhibiting viral replication. If this is the case, it is biologically counterintuitive that E1A—a key viral transactivator essential for efficient viral replication—would induce the expression of a host factor that directly limits its own stability and viral gene expression. The manuscript does not provide a convincing mechanistic explanation for this apparent contradiction.
Specifically, it remains unclear whether TRIM23 upregulation is:
1. a direct transcriptional effect mediated by E1A,
2. an indirect consequence of cellular stress or innate immune activation triggered by E1A, or
3. part of a host-driven antiviral feedback response that is independent of viral intention.
Without clarifying this point, the biological logic of the proposed model lacks coherence. The claim that E1A induces TRIM23, which in turn degrades E1A and suppresses viral transcription, creates a circular and conceptually inconsistent framework. Because this observation constitutes the foundation of the study’s rationale, the failure to resolve this issue undermines the validity of the overall interpretation.
In its current form, the manuscript presents interesting molecular interactions but does not adequately reconcile this fundamental conceptual contradiction. Therefore, I believe the study requires substantial mechanistic clarification before it can be considered for publication. Given the central nature of this issue, I do not recommend publication at this stage.]*

We thank the reviewer for summarizing our work. We also appreciate the reviewer’s very professional and insightful suggestion, which enables us to significantly improve the present work! As your suggestion, we supplement a series of experiments to improve our work, and the results are shown in detail in the second part.

**Part II – Major Issues: Key Experiments Required for Acceptance**
Please use this section to detail the key new experiments or modifications of existing experiments that should be absolutely required to validate study conclusions.

Generally, there should be no more than 3 such required experiments or major modifications for a "Major Revision" recommendation. If more than 3 experiments are necessary to validate the study conclusions, then you are encouraged to recommend "Reject".

**Reviewer #1:**

*[Figures 1 and 2:
The authors used complementary approaches and various readouts (e.g., viral titter, IF, and western blot) to demonstrate that TRIM23 restricts HAdV-C5. These data are strong and convincing. Some of the data shown in Figure 1 (e.g., Fig 1B, 1G, 1H) could be moved to extended panels.
The data in Figure 2 (i.e., RT-qPCR measurement of multiple viral genes) support the authors’ conclusion drawn from the data in Figure 1. Instead of presenting these data as a separate figure, the data could be integrated into Figure 1 and/or shown as extended panels.]*
**R:** Thanks for your comments and questions. As your suggestion, we readjusted the arrangement of the experimental data. We’ve tweaked Figure 2 to the supplementary material section. Please check.

*[Figure 3:
Fig 3H and Fig 3K-3M: The authors concluded from these data that “the ARF domain of TRIM23 mediates the physical interaction with HAdV-C5 E1A, and that both the RING domain and ARF domain of TRIM23 contribute to its antiviral effect”. These functional data do not align with the author’s mechanistic data for the proposed “TRIM23-E1A-p62” axis. Particularly, the mechanistic data that are shown later (Fig 4 and 5) clash with the functional data which show that the isolated ARF, delRING, and delARF (all of which by themselves are reportedly not able to activate autophagy) efficiently restrict HAdV-C5. How do the authors explain their effects? Do these truncation proteins also induce degradation of E1A (which is very unlikely), or could they have additional effects on the cell that may indirectly influence virus replication?]*
**R:** Thanks for your very instructive comments! We are grateful for the reviewer’s very insightful and professional comments, which significantly help us further improve our manuscript. We apologize for the inconsistencies in our previous data presentation; we have now made the necessary corrections. Additionally, we have conducted further experiments confirming that the RING domain of TRIM23 is the key domain responsible for its antiviral effects, and that its function requires the assistance of the ARF domain to be fully realized, as shown in Figure 4. And the results were consistent with our previous findings. Here, we would like to emphasize that our study primarily investigated the role of TRIM23 in the targeted degradation of the E1A viral protein. The RING domain of TRIM23 indeed exerts the primary antiviral activity, and the presence of ARF is required to achieve the same antiviral effect as that of wild-type TRIM23. Viral infection of cells is a complex process. Furthermore, other studies have shown that adenoviruses themselves can activate autophagy and utilize it to promote their own replication. In addition, the results of Figures 3K-M want to show that the RING domain of TRIM23 plays a major role in degrading E1A, and the RING domain needs the presence of the ARF domain when it plays a role in degrading E1A. This does not mean that all truncated variants of TRIM23 exhibit antiviral activity; only those containing the RING domain do so, and the ARF domain must also be present for the antiviral effect of the RING domain to be maximized. Here, TRIM23 serves as a platform for the assembly of the autophagic machinery (PMID:25127057; PMID:40511919), forming a complex with the viral E1A protein and p62. The results are as follows:

**Fig 4A.** Wild-type TRIM23 and its various truncation mutants were separately transfected into A549 cells, followed by infection with HAdV-C5-GFP for 36 hours. Fluorescence was observed under a fluorescence microscope. Scale bar, 200 μm.

**Fig 4B.** Wild-type TRIM23 and its various truncation mutants were separately transfected into A549 cells, followed by infection with HAdV-C5-GFP for 36 hours. Fluorescence was observed under a fluorescence microscope in (A) and TCID50 assays were performed in (B). **, *P* < 0.01; ****, *P* < 0.0001.

**Fig 4C.** Wild-type TRIM23 and its various truncation mutants were separately transfected into A549 cells, followed by infection with HAdV-C5-GFP for 36 hours. Western blot was used to detect the E1A viral protein in cells with the indicated antibodies.

**Fig 4D.** V5-tagged TRIM23 and its mutants (DelRING, BC, DelARF, and ARF) were co-transfected with E1A into HEK293T cells for 48 hours, followed by western blot analysis.

*Figure 4:
[Fig 4A, 4C-F, and 4H: These data strongly suggest that TRIM23-mediated autophagy facilitates degradation of E1A. It has been previously demonstrated that TRIM23 mediates antiviral autophagy via auto-ubiquitination of its ARF domain; this activates TRIM23’s GTPase activity, which is essential for TRIM23’s relocalization to autophagosomes and for TBK1-mediated phosphorylation of the autophagy receptor p62 (PMID: 28871090). The authors should test if a similar mechanism is activated during HAdV infection, and this ultimately leads to p62-mediated E1A degradation.*

*Fig 4B. Solely based on these observations the authors concluded that TRIM23-mediated E1A degradation does not require its E3 ligase activity. RING E3 ligase activity is known to be essential for TRIM23-mediated autophagy because it activates the ARF GTPase via auto-K27-linked ubiquitination. TRIM23 may or may not ubiquitinate E1A. The authors should first compare the ability of WT TRIM23 and a RING catalytic or delRING mutant in their ability to restrict HAdV and to degrade E1A via autophagy. Moreover, to directly test E1A ubiquitination by TRIM23, the authors should include a positive control.]*

**R:** Thanks for your suggestion. First, I’m sorry for some of the results described is not very clear. In our work, we want to show that TRIM23 does not cause ubiquitination of adenovirual E1A protein, ruling out the possibility that it degrades E1A through the proteasome pathway. In our study, the RING domain of TRIM23 is the key domain responsible for its antiviral effects, and that its function requires the assistance of the ARF domain to be fully realized. This article (PMID: 28871090) primarily discusses the mechanism by which TRIM23 regulates autophagy to inhibit viral replication during HSV-1 infection, while our study focuses on how TRIM23 autophagically degrades the viral E1A protein. When viruses infect cells, some viruses activate autophagy to aid their own replication, while the host also utilizes autophagy to degrade viral proteins and inhibit viral replication; host factors play a crucial role in both processes. In our study, TRIM23 lacking the RING domain still exhibited some antiviral activity **(Figs 4A and B)**. However, the antiviral activity of the DelARF variant-which contains the RING domain but lacks the ARF domain-was comparable to that of the wild-type TRIM23. This suggests that the antiviral activity of TRIM23 is primarily mediated by the RING domain, while the ARF domain also contributes to antiviral activity, albeit to a lesser extent than the RING domain. In line with this, E1A was cotransfected with the V5-tagged TRIM23 and its various mutants into HEK293T cells to determine the domain of TRIM23 involved in the degradation of E1A. The results indicate that, in the presence of only the BC domain, the E1A protein showed virtually no difference compared to the control **(Fig 4D)**. The results are as follows:

**Fig 4A.** Wild-type TRIM23 and its various truncation mutants were separately transfected into A549 cells, followed by infection with HAdV-C5-GFP for 36 hours. Fluorescence was observed under a fluorescence microscope. Scale bar, 200 μm.

**Fig 4B.** Wild-type TRIM23 and its various truncation mutants were separately transfected into A549 cells, followed by infection with HAdV-C5-GFP for 36 hours. Fluorescence was observed under a fluorescence microscope in (A) and TCID50 assays were performed in (B). **, *P* < 0.01; ****, *P* < 0.0001.

**Fig 4D.** V5-tagged TRIM23 and its mutants (DelRING, BC, DelARF, and ARF) were co-transfected with E1A into HEK293T cells for 48 hours, followed by western blot analysis.

**Fig 3C.** HEK293T cells were cotransfected with E1A, TRIM35-V5, TRIM23-V5, and pHA-Ub WT. TRIM35-V5 serves as a positive control. Then, ubiquitination assays were performed and blotted with indicated antibodies.

*[Fig 4I. These data do not unbiasedly test whether E1A enhances TRIM23-mediated autophagy; the authors need to include an additional control where there is no E1A.]*

**R:** Thanks for your suggestion. In Figure 4I, we aim to demonstrate that as TRIM23 expression increases, the autophagy it induces becomes more pronounced, leading to more extensive degradation of the E1A protein. Furthermore, LC3I is increasingly converted to LC3II.

*[Fig 4J. p62 is generally degraded upon autophagy induction, which the authors also showed in Fig 5H. However, in this figure (4J) the authors show that HAdV-C5 infection upregulates p62. Why is this?]*

**R:** Thanks for your suggestion. After infecting cells, adenoviruses activate autophagy and utilize host materials to facilitate their own replication. Overall, intracellular autophagy levels increase, as evidenced not only by elevated LC3 expression but also by a greater conversion of LC3I to LC3II and increased p62 expression, indicating a general rise in autophagy activity. Although p62 protein expression is generally negatively correlated with autophagy, this is not an absolute rule; therefore, autophagy levels cannot be determined solely based on p62 expression.

*[Figure 5:
Fig 5D: A control condition with only E1 and no p62 should be included in this assay.
Fig 5E: Fig 5D and 5E data are somehow redundant.
Fig 5F and 5G: The authors propose that TRIM23 functions as an adaptor. To rigorously test this, the authors should assess p62-E1A interaction in WT and TRIM23 knockout cells.
Fig 5H: It is unclear what conclusion the authors want to draw from these data as there is no E1A included in this assay. What is shown in this figure (i.e., TRIM23 induces degradation of p62) has been previously reported.]*
**R:** Thanks for your suggestion. According to your suggestion, we removed Fig 5E and Fig 5H, and we reperformed some experiments, re-set the Fig 5D control group and the experimental group. In addition, we conducted additional experiments to validate the role of TRIM23 in the interaction between p62 and E1A. TRIM23 serves as a platform for the assembly of the autophagic machinery. TRIM23 recruits the selective autophagy receptor p62, which facilitates the E1A-p62 interaction and subsequently directs E1A to phagophores for degradation. As follows:

**Fig 5G.** HEK293T cells were co-transfected with Flag-tagged p62 expression plasmid, Myc-tagged E1A expression plasmid, and V5-tagged TRIM23 expression plasmid at various concentrations for 36 hours, followed by western blot analysis.

**Fig 5F.** Immunoblot analysis of lysates of HEK293T co-transfected with E1A and shp62, together with or without V5-tagged TRIM23 for 48 h.

**Fig 5J.** Lysates from HEK293T co-transfected with plasmids encoding E1A and Flag-p62 together with or without TRIM23-V5, and treated with BafA1 (20 mM), were subjected to co-IP assays using an anti-E1A antibody, followed by immunoblot analysis with the indicated antibodies.

**Fig 5K.** Lysates from HEK293T co-transfected with specific shRNA targeting TRIM23 and plasmids encoding E1A and Flag-p62 were subjected to co-IP assays using an anti-E1A antibody, followed by immunoblot analysis with the indicated antibodies.

*[Figure 6:
Fig 6D: Opposing to these data, the authors show higher levels of E1A in TRIM23 depleted cells in Figure 1J. Please address this discrepancy.]*
**R:** Thanks for your suggestion. Figure 6D shows the differences in intracellular E1A viral protein levels in various tumor cells following infection with conventional oncolytic adenovirus and recombinant oncolytic adenovirus carrying shTRIM23, respectively. Although the MOI used is consistent with that in Fig. 1J, the experiment in Fig. 1J utilized wild-type adenovirus type 5, whereas Fig. 6D employed a modified adenovirus that is more readily able to infect tumor cells. This modified virus exhibits greater affinity for cell binding and enhanced replication capacity, resulting in the faster release of progeny viral particles into the supernatant. Consequently, less E1A protein remains retained within the cells.

*[Figure 7:
Fig 7J and 7L: These data are not consistent with the results shown in Fig 6D.]*

**R:** Thanks for your suggestion. The results of Figures 7J and 7L show the levels of the viral E1A protein in tumor tissue. The recombinant oncolytic adenovirus carrying shTRIM23 replicated more efficiently and persisted longer in mice, resulting in higher viral loads and higher levels of the viral E1A protein. Figure 6D, on the other hand, shows the levels of the viral E1A protein retained within cells.

**Reviewer #2:** Major issues:
*[1：No methods or useful description is provided for the mass-spec data shown in Figure 1A. The TRIM23 overexpressing cell line is also not described. The mass-spec data either needs to be publicly archived or presented as supplementary data.]*
**R:** Thanks for your suggestion. We have made the revision according to your suggestion, please check.

**Mass spectrometry**

Digest proteins (250 µg per sample) in whole-cell lysates according to the FASP protocol. In brief, remove detergents, DTT, and other low-molecular-weight components using 100 µL of UA buffer via centrifugation-assisted repeated ultrafiltration. The protein suspension was added to 100 µL of 25 mM NH₄HCO₃, 5 µg of trypsin was added, and digestion was performed overnight at 37°C. After digestion, peptides from each sample were desalted on a C18 column, concentrated by vacuum centrifugation, and resuspended in 40 µL of 0.1% (vol/vol) formic acid. Liquid chromatography-tandem mass spectrometry (LC-MS/MS) analysis was performed using a Q-Exactive mass spectrometer (Thermo Fisher Scientific) coupled with an Easy nLC system (Proxeon Biosystems, now part of Thermo Fisher Scientific). The analysis duration was 120 minutes, with technical support provided by Shanghai Applied Protein Technology Co., Ltd. (Shanghai, China).

Mass spectrometry data were analyzed using MaxQuant software (version 1.6.14) and aligned against the UniProt proteome database. Quantification was performed using a label-free quantification algorithm. Quantifiable proteins were defined as those identified at least twice across three biological replicates. Proteins with an adjusted p-value <0.05 and a fold change in abundance >2 were identified as differentially expressed proteins between the empty vector and the adenovirus E1A overexpression (Table S1).

**Construction of A549 cells stably overexpressing TRIM23**

Use Lipofectamine 2000 to transfect the retroviral construct pQCXIN-TRIM23 and the empty vector pQCXIN into the AmphoPack-293 packaging cell line (631505, Clontech). Forty-eight hours after transfection, collect the viral supernatant from the transfected cells and use it to transduce A549 cells cultured in a six-well plate. Repeat this process 48 hours after the initial transduction. Pass the transduced cells and screen them in medium supplemented with 1,000 μg/mL G418. Clone the surviving cells into a 96-well plate for expansion and detect TRIM23 overexpression via quantitative reverse transcription PCR (RT-qPCR).

*[2：The authors use HEK293T cells in a number of experiments (see figure 3 for example), but the blots never show any endogenous E1A in the control transfected/infected cells. This needs to be explained.]*
**R:** Thanks for your suggestion. We have verified in our previous article that the endogenous expression of E1A protein is very weak, and there is a control at each time of the experiment. Please refer to PMID: 37578239.

*[3：The generation of the oncolytic virus is a bit vague. What is deleted from the Ad5 genome? Is it expected to replicate in normal cells? Additionally, that point is not addressed at all. Does this oncolytic virus infect non-tumor cells? Does it replicate well in these cells. How safe would this virus be? The authors describe that they focus on better replication in tumor cells, but they also need to address the selectivity of the virus as well as safety.]*
**R:** Thanks for your suggestion. We have made modifications and added more information in the article. The E1 region of the backbone of a tumor-selective oncolytic adenovirus plasmid pZD55 vector in which *E1B55K* *gene* had been deleted was used to construct recombined oncolytic adenovirus. The ki67 promoter was used to construct the recombined oncolytic adenovirus, OAV-shTRIM23. The ki67 promoter has tumor cell selectivity lead to be replicated in tumor cells. So, it doesn’t replicate in normal cells and can’t infect non-tumor cells. In addition, we tested the cytotoxicity of recombinant oncolytic adenovirus (OAV-shTRIM23) and traditional oncolytic adenovirus (OAV) on kidney and liver and found that there was no significant difference between them and PBS. This shows that OAV-shTRIM23 is safe as well as OAV.

*[4：Given that p62 is primarily localized to the cytoplasm, and because TRIM23 is know to target E1A for degradation, and E1A is known to bind p62, the authors might speculate that the association with E1A is indirect and bridged by TRIM23. What domains of TRIM23 are needed to interact with p62? If they are the same as needed for E1A, this would lend some support to this model. Alternatively, are they forming a trimeric complex? This could be demonstrated by serial co-IPs.]*

**R:** Thanks for your suggestion. We supplemented a series of co-immunoprecipitation experiments. The results showed that the BC domain of TRIM23 is a key region for interaction with p62, and previous studies have shown that E1A interacts with the ARF domain of TRIM23, that is, p62 and E1A interact with different domains of TRIM23, and the three form a complex. TRIM23 acts as a bridge to facilitate the delivery of viral E1A protein to p62, and then enters the autophagosome for degradation. As follows:

**Fig 5C.** Co-immunoprecipitation and western blot analysis using an anti-Flag antibody after transfecting HEK293T cells with V5-TRIM23 and its truncated mutants along with Flag-tagged p62.

**Fig 5J.** Lysates from HEK293T co-transfected with plasmids encoding E1A and Flag-p62 together with or without TRIM23-V5, and treated with BafA1 (20 mM), were subjected to co-IP assays using an anti-E1A antibody, followed by immunoblot analysis with the indicated antibodies.

**Fig 5K.** Lysates from HEK293T co-transfected with specific shRNA targeting TRIM23 and plasmids encoding E1A and Flag-p62 were subjected to co-IP assays using an anti-E1A antibody, followed by immunoblot analysis with the indicated antibodies.

**Reviewer #3:** *[The title of this review is “TRIM23 prevents adenovirus replication by p62-mediated selective autophagic degradation of viral E1A protein”.

The manuscript investigates the antiviral role of TRIM23 against human adenovirus type 5 (HAdV-C5), proposing that TRIM23 interacts with the viral protein E1A, promotes its degradation via an autophagosome-dependent mechanism, and suppresses transcription of HAdV-C5 viral genes. While the study presents potentially interesting findings, there is a fundamental conceptual issue that significantly weakens the overall premise of the work.
The entire story is built upon the initial observation that TRIM23 is upregulated in E1A-overexpressing cells (first result part; through proteomic analysis….). This finding serves as the primary rationale and conceptual starting point of the manuscript. In other words, the authors justify the investigation of TRIM23 as an antiviral factor based on its induction by E1A. However, this central rationale raises a critical and unresolved paradox.
According to the authors’ model, TRIM23 promotes degradation of E1A and suppresses viral gene transcription, thereby inhibiting viral replication. If this is the case, it is biologically counterintuitive that E1A—a key viral transactivator essential for efficient viral replication—would induce the expression of a host factor that directly limits its own stability and viral gene expression. The manuscript does not provide a convincing mechanistic explanation for this apparent contradiction.
Specifically, it remains unclear whether TRIM23 upregulation is:
1. a direct transcriptional effect mediated by E1A,
2. an indirect consequence of cellular stress or innate immune activation triggered by E1A, or
3. part of a host-driven antiviral feedback response that is independent of viral intention.
Without clarifying this point, the biological logic of the proposed model lacks coherence. The claim that E1A induces TRIM23, which in turn degrades E1A and suppresses viral transcription, creates a circular and conceptually inconsistent framework. Because this observation constitutes the foundation of the study’s rationale, the failure to resolve this issue undermines the validity of the overall interpretation.
In its current form, the manuscript presents interesting molecular interactions but does not adequately reconcile this fundamental conceptual contradiction. Therefore, I believe the study requires substantial mechanistic clarification before it can be considered for publication. Given the central nature of this issue, I do not recommend publication at this stage.]*

**R:** Thanks for your suggestion. We are very sorry that it may not be clearly described. Virus infection is a very complex process. When the virus invades the cell, it will cause the host to produce a resistance response, which may increase the expression of TRIM23. With the progress of infection, the expression of viral E1A protein will increase, and then TRIM23 will be negatively regulated, so that the expression level of TRIM23 will decrease as viral E1A increased. According to our previous protein spectrum analysis results, the expression level of TRIM23 in the exogenously transfected E1A overexpression cells was lower than that in the control cells. Now, in our work, we detected the expression level of TRIM23 during HAdV-C5 infection. There is indeed a process of increasing first and then decreasing. At the same time, we also detected the mRNA level of TRIM23 during adenovirus infection, and the results are also the same. In addition, we also used dose-dependent experiments to detect the effect of E1A on the expression level of TRIM23-V5. The results showed that the protein stability of TRIM23 decreased with the increase of E1A expression, which was consistent with the previous mass spectrometry results. However, we would like to point out that the process of viral infection of cells and the process of exogenous transfection of proteins may be partially different, because the response of the virus to stimulate the cells is cascade, multi-level and complex. The results are shown in the following：

**Fig 2A.** Endogenous TRIM23 protein was detected by western blot analysis in A549 cells at 0, 6-, 9-, 12-, 24-, and 36-hours post-infection with HAdV-C5 at a multiplicity of infection (MOI) of 20.

**Fig 2B.** Quantification of relative TRIM23 levels is shown in (B). *, *P* < 0.05; **, *P* < 0.01; ***, *P* <0.001; ****, *P* <0.0001.

**Fig S3A.** Endogenous TRIM23 mRNA levels were detected by RT-qPCR analysis in A549 cells at 0, 6-, 9-, 12-, 24-, and 36-hours post-infection with HAdV-C5 at a multiplicity of infection (MOI) of 20. *, *P* < 0.05; ***, *P* < 0.001; ****, *P* < 0.0001.

**Fig S3B.** Endogenous E1A mRNA levels were detected by RT-qPCR analysis in A549 cells at 0, 6-, 9-, 12-, 24-, and 36-hours post-infection with HAdV-C5 at a multiplicity of infection (MOI) of 20. ***, *P* < 0.001; ****, *P* < 0.0001.

**Part III – Minor Issues: Editorial and Data Presentation Modifications**
Please use this section for editorial suggestions as well as relatively minor modifications of existing data that would enhance clarity.

**Reviewer #1:** *[1. Abstract: Remove the word “directly” from “TRIM23 interacts directly with the viral E1A”, as such conclusion cannot be made based on the cell-based interaction data shown. This requires in vitro binding study using recombinant purified proteins.]*

**R:** Thanks for your suggestion. We have supplemented the experiments with in vitro interaction assays. As follows:

**Fig 2H.** GST or GST-TRIM23 was transfected into HEK293T cells. Thirty-six hours after transfection, cell lysates were collected and incubated with Glutathione Sepharose 4 Fast Flow for 6 hours. Cell lysates from cells expressing E1A were then added, and western blot analysis was performed.

*[2. Introduction: The authors mention certain TRIM proteins with roles in autophagy for which there is only limited literature. By contrast, they omit the literature describing the most well-studied TRIM proteins in viral autophagy, which is TRIM5a and TRIM23. Please also include these. Moreover, the authors should introduce and summarize the previous findings on TRIM23 and its reported role/activation mechanism in autophagy and antiviral defense and cite the relevant literatures (PMID: 25587751; PMID: 33669846; PMID: 28871090; PMID: 40360474)]*

**R:** Thanks for your suggestion. We have made modifications and supplemented the relevant literature. Please check.

*[3. Fig 1A. Why is the viral titter lower at 48 h compared to 24 h in Fig 1A, whereas most of the data (Fig. 1C, D, E, J etc.) show that HAdV-C5 replicates efficiently at 48 h compared to 24 h.]*

**R:** Thanks for your suggestion. The cell state at that time may not be very good after virus infection, so that the virus titer did not grow for 48 hours. We repeated the experiment, and the results are as follows:

**Fig 1A.** A549 cells stably overexpressing TRIM23 and control cells were infected with HAdV-C5 at an MOI of 5. Cell supernatants were collected 24- or 48-hours post-infection. Viral titers in infected cell supernatants were determined by the TCID50 assay. ***, *P* < 0.001.

*[4. Please add the molecular markers for all the immunoblots.]*

**R:** Thanks for your suggestion. We have added the molecular markers for all the immunoblots. Please check.

*[5. Fig 1H. Please improve the quality of these images. If possible, please also do quantification.]*

**R:** Thanks for your suggestion. Because the condition of the cells deteriorates after viral infection-with some cells even becoming shriveled and rounded-the statistical software is unable to accurately identify the number and condition of the affected cells.

*[6. Fig 3H. The authors should include a negative control in this experiment; this is needed to rule out that the proteins are not non-specifically binding to the beads.]*

**R:** Thanks for your suggestion. We have repeated the experiment and included a negative control. Please check. As follows:

**Fig 2J.** Co-immunoprecipitation and western blot analysis using an anti-E1A antibody after transfecting HEK293T cells with V5-TRIM23 and its truncated mutants along with E1A.

*[7. Fig 3A. The authors should quantify these data (ideally from three independent blots) accounting for the GAPDH that is also increasing over time.]*

**R:** Thanks for your suggestion. We have now included the corresponding grayscale value calculations. As follows:

**Fig 2B.** Endogenous TRIM23 was detected by western blot analysis in A549 cells at 0, 6-, 9-, 12-, 24-, and 36-hours post-infection with HAdV-C5 at a multiplicity of infection (MOI) of 20. Quantification of relative TRIM23 levels is shown in (B). *, *P* < 0.05; **, *P* < 0.01; ***, *P* <0.001; ****, *P* <0.0001.

*[8. Fig 3E. Why are the bands for V5-TRIM23 running differently in WCL vs. IP?]*

**R:** Thanks for your suggestion. This may be due to improper preparation of the protein gel used for loading. We repeated the experiment, and the results are as follows:

**Fig 2F.** HEK293T cells co-transfected with E1A and V5-tagged TRIM23 plasmids for 48 hours were subjected to co-immunoprecipitation using an anti-V5 antibody, followed by western blot analysis.

*[9. Fig 3H. Why does the TRIM23-V5 blot show two bands in both WCL as well as IP?]*

**R:** Thanks for your suggestion. This may be due to the V5-tagged antibody, which was reused multiple times during the western blot analysis. We have repeated the experiment, using freshly prepared antibody to detect the corresponding protein each time. As follows:

**Fig 2J.** Co-immunoprecipitation and western blot analysis using an anti-E1A antibody after transfecting HEK293T cells with V5-TRIM23 and its truncated mutants along with E1A.

*[10. Fig 3L. The interpretation that “there was no significant difference in E1A viral protein compared with the control” seems to be incorrect. The authors should quantify the E1A blot and perform statistical analysis.]*

**R:** Thanks for your suggestion. We repeated the experiment and calculated the grayscale values. As follows:

**Fig 4C.** Wild-type TRIM23 and its various truncation mutants were separately transfected into A549 cells, followed by infection with HAdV-C5-GFP for 36 hours. Western blot was used to detect the E1A viral protein in cells.

*[11. Fig 5C: These data are not correctly explained/interpreted in the related text in the Results section.]*

**R:** Thanks for your suggestion. We have re-interpreted the result in the manuscript, please check.

*[12. Fig 5D: In the first lane, there appears to be a non-specific band in the Myc (E1A) blot. The authors should indicate this in the blot and describe it in the legend. The authors did not include a condition with no E1A in the rest of the figure panels; please include a “no E1A” control.]*

**R:** Thanks for your suggestion. We repeated this experiment and included a “no E1A” control. As follows:

**Fig 5G.** HEK293T cells were co-transfected with Flag-tagged p62 expression plasmid, Myc-tagged E1A expression plasmid, and V5-tagged TRIM23 expression plasmid at various concentrations for 36 hours, followed by western blot analysis.

*[13. Fig 5E: TRIM23 is not inducing p62 degradation here when tested in the presence of E1A, which is in contrast to the data shown in Fig 5H. Why is this?]*

**R:** Thanks for your suggestion. This is due to differences in the transfection dose of p62-Flag. We adjusted the transfection dose and repeated the experiment. As follows:

**Fig 5E.** HEK293T cells were co-transfected with E1A expression plasmid and Flag-tagged p62 expression plasmid, together with or without V5-tagged TRIM23 expression plasmid for 36 hours, followed by western blot analysis.

*[14. Fig 6C. The quality of these images should be improved.]*

**R:** Thanks for your suggestion. We have used the raw images taken during the experiment to enhance clarity. Please check.

*[15. Discussion: In the statement “TRIM23 exerts both positive and negative regulatory roles in the antiviral type I interferon response [36, 37]”, both references (36 and 37) are not describing TRIM23’s role in IFN responses, but they describe its role in antiviral autophagy. Please correct the sentence.]*

**R:** Thanks for your suggestion. We have revised the references and reviewed the entire text again. Please check.

**Reviewer #2:** Minor issues
*[1：There are minor issues with the language. Line 181, sentence is incomplete. Line 161 the word detected should be studied, examined, investigated, etc. Some abbreviations are not defined (DEG, RTCA), etc. The writing is pretty good but needs careful editing.]*
**R:** Thanks for your suggestion. We re-examined the language and sentences of the full text and made modifications. Please check.

*[2：For the figures, use the same font for the panel labels as the rest of the figure (A, B, C, etc)]*
**R:** Thanks for your suggestion. We have made modifications to ensure consistency, please check.

*[3：There are lots of references, but they sometimes seem like odd choices (refs 11, 27 are okay but not complete, etc). I suggest going through the citations very carefully.]*
**R:** Thanks for your very instructive comments! We have modified the references you mentioned, and then checked and corrected the references cited in the full text.

*[4：Be consistent with abbreviations. qRT-PCR was used for much of the data, but is called qPCR and other things variably in the manuscript.]*
**R:** Thanks for your suggestion. We have corrected the abbreviation in the full text. Please check.

*[5：Some figure legends state the number of times a given experiment was performed, others do not state this. The n for every experiment should be provided in all of the figure legends.]*
**R:** Thanks for your suggestion. We have corrected it in full text. Please check.

*[6：I don't think ref 5 is a great choice for this point. Its just a single research paper. No refs for the plasmids used in the HAdV rescue are cited on page 12.]*
**R:** Thanks for your suggestion. We have corrected it. Please check.

*[7：As TRIM23 is induced by interferon (IFN), is it simply possible that the HAdV infection is just inducing an IFN response that increases TRIM infection? Not that PML is a TRIM and there is a long history of E1A altering PML bodies (PMID: 13130097, etc).]*
**R:** Thanks for your very instructive comments! We have confirmed that IFN does not induce TRIM23 expression in **Figs S3C and D**. Virus infection is a very complex process. When the virus invades the cell, it will cause the host to produce a resistance response. In our study, in the early stage of HAdV-C5 infection, TRIM23 will increase due to virus stimulation, and the expression level of TRIM23 will gradually decrease with the increase of viral E1A protein expression. Viruses, which require the cellular machinery for their replication, have evolved different ways to counteract the action of IFN by inhibiting IFN signalling, by blocking the activities of specific antiviral mediators or by altering PML expression and/or localization on nuclear bodies (PMID: 11704856). Regulatory proteins encoded by several DNA viruses associate with and sometimes cause catastrophic changes to PML NBs by a variety of mechanisms (PMID: 11704855). The promyelocytic leukaemia (PML) protein localizes both in the nucleoplasm and in matrix-associated multi-protein complexes known as nuclear bodies (NBs). The function of PML NBs is still unclear, some results indicate that they may represent preferential targets for viral infections and that PML could play a role in the mechanism of the antiviral action of IFNs. Viruses interfere with PML-NB in two ways: 1) some viral proteins can associate with PML-NB proteins and/or lead to the destruction and lysis of this subnuclear compartment, thus aiding viral gene expression and disabling the host's innate immunity; 2) the parental genomes of some nuclear-replicating DNA viruses associate preferentially with PML-NBs, which presumably serves to assist in viral gene expression or replication (PMID: 14626429). Here, I thought that TRIM23 is not a PML.

*[8：Figs. 3K and 3L. The authors switched the assays here form retrovirus transduction of TRIM23 to transient expression. It is critical that data with the wild-type TRIM23 protein be included alongside the mutant TRIM23s to verify the assay.]*
**R:** Thanks for your suggestion. In Figs. 3K and 3L, different truncated mutants of TRIM23 need to be overexpressed respectively, and transient transfection can meet the expression requirements of TRIM23 and its mutants, and we detected that the protein expression levels of these plasmids were high. As follows:

**Fig 4C.** Wild-type TRIM23 and its various truncation mutants were separately transfected into A549 cells, followed by infection with HAdV-C5-GFP for 36 hours. Western blot was used to detect the E1A viral protein in cells.

*[9：Figs. 4C and 4H - lighter E1A exposures of the Western blots are needed.]*
**R:** Thanks for your suggestion. We have corrected it. Please check.

*[10：Figure 5. " Taken together, these results indicate that TRIM23 accelerates the autophagic degradation of E1A by promoting its interaction with p62." No part of that statement has been conclusively shown, but additionally, they have not looked at the interaction of the TRIM23, E1A, or p62.]*
**R:** Thanks for your suggestion. We have conducted a series of experiments to validate the interactions between TRIM23, E1A, and p62. As follows:

**Fig 5J.** Lysates from HEK293T co-transfected with plasmids encoding E1A and Flag-p62 together with or without TRIM23-V5, and treated with BafA1 (20 mM), were subjected to co-IP assays using an anti-E1A antibody, followed by immunoblot analysis with the indicated antibodies.

**Fig 5K.** Lysates from HEK293T co-transfected with specific shRNA targeting TRIM23 and plasmids encoding E1A and Flag-p62 were subjected to co-IP assays using an anti-E1A antibody, followed by immunoblot analysis with the indicated antibodies.

*[11：Fig. 6G- the authors should refer to this data as anti-proliferative effects, not anti-tumor effects. These are in vitro experiments on cell growth.]*

**R:** Thanks for your suggestion. We have corrected it. Please check.

**Reviewer #3:** *[The manuscript investigates the antiviral role of TRIM23 against human adenovirus type 5 (HAdV-C5), proposing that TRIM23 interacts with the viral protein E1A, promotes its degradation via an autophagosome-dependent mechanism, and suppresses transcription of HAdV-C5 viral genes. While the study presents potentially interesting findings, there is a fundamental conceptual issue that significantly weakens the overall premise of the work.
The entire story is built upon the initial observation that TRIM23 is upregulated in E1A-overexpressing cells (first result part; through proteomic analysis….). This finding serves as the primary rationale and conceptual starting point of the manuscript. In other words, the authors justify the investigation of TRIM23 as an antiviral factor based on its induction by E1A. However, this central rationale raises a critical and unresolved paradox.
According to the authors’ model, TRIM23 promotes degradation of E1A and suppresses viral gene transcription, thereby inhibiting viral replication. If this is the case, it is biologically counterintuitive that E1A—a key viral transactivator essential for efficient viral replication—would induce the expression of a host factor that directly limits its own stability and viral gene expression. The manuscript does not provide a convincing mechanistic explanation for this apparent contradiction.
Specifically, it remains unclear whether TRIM23 upregulation is:
1. a direct transcriptional effect mediated by E1A,
2. an indirect consequence of cellular stress or innate immune activation triggered by E1A, or
3. part of a host-driven antiviral feedback response that is independent of viral intention.
Without clarifying this point, the biological logic of the proposed model lacks coherence. The claim that E1A induces TRIM23, which in turn degrades E1A and suppresses viral transcription, creates a circular and conceptually inconsistent framework. Because this observation constitutes the foundation of the study’s rationale, the failure to resolve this issue undermines the validity of the overall interpretation.
In its current form, the manuscript presents interesting molecular interactions but does not adequately reconcile this fundamental conceptual contradiction. Therefore, I believe the study requires substantial mechanistic clarification before it can be considered for publication. Given the central nature of this issue, I do not recommend publication at this stage.]*

**R:** Thanks for your suggestion. We are very sorry that it may not be clearly described. Virus infection is a very complex process. When the virus invades the cell, it will cause the host to produce a resistance response, which may increase the expression of TRIM23. With the progress of infection, the expression of viral E1A protein will increase, and then TRIM23 will be negatively regulated, so that the expression level of TRIM23 will decrease as viral E1A increased. According to our previous protein spectrum analysis results, the expression level of TRIM23 in the exogenously transfected E1A overexpression cells was lower than that in the control cells. Now, in our work, we detected the expression level of TRIM23 during HAdV-C5 infection. There is indeed a process of increasing first and then decreasing. At the same time, we also detected the mRNA level of TRIM23 during adenovirus infection, and the results are also the same. In addition, we also used dose-dependent experiments to detect the effect of E1A on the expression level of TRIM23-V5. The results showed that the protein stability of TRIM23 decreased with the increase of E1A expression, which was consistent with the previous mass spectrometry results. However, we would like to point out that the process of viral infection of cells and the process of exogenous transfection of proteins may be partially different, because the response of the virus to stimulate the cells is cascade, multi-level and complex. The results are shown in the following:

**Fig 2A.** Endogenous TRIM23 was detected by western blot analysis in A549 cells at 0, 6-, 9-, 12-, 24-, and 36-hours post-infection with HAdV-C5 at a multiplicity of infection (MOI) of 20.

**Fig 2B.** Quantification of relative TRIM23 levels is shown in (B). *, *P* < 0.05; **, *P* < 0.01; ***, *P* <0.001; ****, *P* <0.0001.

**Fig S3A.** Endogenous TRIM23 mRNA levels were detected by RT-qPCR analysis in A549 cells at 0, 6-, 9-, 12-, 24-, and 36-hours post-infection with HAdV-C5 at a multiplicity of infection (MOI) of 20. *, *P* < 0.05; ***, *P* < 0.001; ****, *P* < 0.0001.

**Fig S3B.** Endogenous E1A mRNA levels were detected by RT-qPCR analysis in A549 cells at 0, 6-, 9-, 12-, 24-, and 36-hours post-infection with HAdV-C5 at a multiplicity of infection (MOI) of 20. ***, *P* < 0.001; ****, *P* < 0.0001.
