# Supplementary material for: TRIM23 prevents adenovirus replication by p62-mediated selective autophagic degradation of viral E1A protein
Source: PLoS Pathog. 2026 Sep 8;22(9):e1014578. doi: 10.1371/journal.ppat.1014578 (PMC13577515; doi:10.1371/journal.ppat.1014578)
Supplement: Supplementary file 3 [file ppat.1014578.s008.docx]

The following are our responses to the comments (*in Italics*) of the reviewers and the changes made in this manuscript (**PPATHOGENS-D-26-00171R1**).

**Reviewers’ Comments:**

Reviewer’s Responses to Questions

**Part I - Summary**
Please use this section to discuss strengths/weaknesses of study, novelty/significance, general execution and scholarship.

*[****Reviewer #1:*** *TRIM23 has been previously shown to induce antiviral autophagy that depends on its RING E3 ligase activity. The authors mis-interpreted in the first submission (partly based on insufficient experimental evidence) that TRIM23, in an E3-ligase activity independent manner, facilitates autophagic degradation of E1A. In the revised manuscript, the authors have performed additional experiments (demonstration of autoubiquitination of TRIM23, inclusion of E3 ligase deficient mutants in their assay, etc.) to systematically assess the mechanism for TRIM23-mediated autophagic degradation of E1A. Based on their new data, the authors have concluded that “TRIM23 promotes E1A degradation through a mechanism dependent of its canonical E3 ligase activity”. The authors also addressed several other concerns.
However, unfortunately, there are still substantial issues and inconsistencies in the data and data interpretation, manuscript writing, and the conclusions drawn.]*

We thank the reviewer for summarizing our work. We also appreciate the reviewer’s very professional and insightful suggestion, which enables us to significantly improve the present work! As your suggestion, we supplement some experiments to improve our work, and the results are shown in detail in the second part. After addressing the issues raised by the reviewer, we do think the current manuscript could be considered for publication in the PLoS Pathogens.

*[****Reviewer #2:*** *None]*

*[****Reviewer #3:*** *The title of this review is “TRIM23 prevents adenovirus replication by p62-mediated selective autophagic degradation of viral E1A protein”.
The authors have satisfactorily addressed my major concern regarding the apparent conceptual inconsistency between E1A and TRIM23. The revised text no longer implies that E1A directly induces TRIM23 expression, and the newly provided infection kinetics support the conclusion that TRIM23 is induced during HAdV-C5 infection. In addition, the mechanistic data supporting TRIM23-mediated autophagic degradation of E1A are convincing.]*

We thank the reviewer for summarizing our work and stating that “the mechanistic data supporting TRIM23-mediated autophagic degradation of E1A are convincing”. We also appreciate the reviewer’s very professional and insightful suggestion, which enables us to significantly improve the present work!

**Part II – Major Issues: Key Experiments Required for Acceptance**
Please use this section to detail the key new experiments or modifications of existing experiments that should be absolutely required to validate study conclusions.

Generally, there should be no more than 3 such required experiments or major modifications for a "Major Revision" recommendation. If more than 3 experiments are necessary to validate the study conclusions, then you are encouraged to recommend "Reject".

**Reviewer #1: Major comments:**

*[Fig 4A-C: The authors show in their data that WT-TRIM23 potently restricts AdV. While delRING-TRIM23 mostly loses its antiviral activity, delARF-TRIM23 still shows potent antiviral activity (comparable to WT-TRIM23). However, the authors interpret (on page 12, first paragraph) that the “antiviral effect is primarily mediated by RING domain and requires the presence of the ARF domain”, the latter is not supported by their data (although they show marginal antiviral activity of isolated ARF domain). Importantly, the observation that “del-ARF still retains antiviral activity” and “E1A interacts with TRIM23 via its ARF domain” do not support their working model and conclusions.]*
**R:** Thanks for your comments and questions. What we want to express here is that the RING domain plays a major antiviral role, and the ARF domain also has a little antiviral effect. The DelARF domain contains the RING domain (only the ARF domain is missing), so it still has a significant antiviral effect, and its antiviral effect is more significant than the truncated mutants (DelRING and ARF) containing the ARF domain without the RING domain, as shown in Figure 4A-D. In addition, it has been reported in many studies that TRIM family proteins containing the classical RBCC domain often exert antiviral effects, and most of them interact with the target protein in the C-terminal region, and the N-terminal RING domain plays an antiviral role (References to *PMID:29899090、PMID:32562145、PMID:38275298、PMID:40435148, etc*). We also verified that the ARF region not only exerts little antiviral activity, but also is a key region for interaction with E1A. In summary, the RING domain of TRIM23 plays a major antiviral activity, and it requires the assistance of the ARF domain to maximize its antiviral activity both in vitro and in vivo. Although the presence of the RING domain (such as: DelARF) has a very significant antiviral effect, it is still not as effective as the full-length TRIM23. Therefore, we believe that the antiviral activity of TRIM23 depends on the combination of the RING domain and the ARF domain.

*[Fig 4E-H: Moreover, the authors here show that both delRING and delARF mutants (but not BC-V5, and ARF-V5) can efficiently degrade E1A; these data create additional inconsistencies. The authors did not correctly interpret this data (i.e., they wrote that only BC-V5 failed to degrade, implying that ARF-V5 still degrades, the latter is not evident).
Importantly, the authors again conclude that “both the RING domain and ARF domain of TRIM23 contribute to its antiviral effect”.]*
**R:** Thanks for your comments and questions. According to your suggestion, we repeated Figs.4E and 4H. The results showed that the expression of E1A decreased with the increasing DelRING or ARF, but its degradation effect was not as significant as that of the mutant containing the RING domain (DelARF). In our conclusions, when TRIM23 degrades E1A viral protein to play an antiviral role, the RING domain plays a major antiviral activity, and ARF plays a light antiviral effect, and the simultaneous presence of the two can maximize the antiviral effect. As follows:

**Fig. 4E** Immunoblotting of protein extracts from HEK293T cells co-transfected with E1A-expressing plasmid and varying concentrations of V5-tagged TRIM23 truncated mutant (DelRING), followed by western blot analysis.

**Fig. 4H** Immunoblotting of protein extracts from HEK293T cells co-transfected with E1A-expressing plasmid and varying concentrations of V5-tagged TRIM23 truncated mutant (ARF), followed by western blot analysis.

*[Fig 5G: Degradation of E1A by p62 is not evident (2nd vs. 3rd lane) and the effect of TRIM23 is marginal. These observations are not consistent with the authors' other data shown (e.g., Fig. 5E, 5F).]*
**R:** Thanks for your comments and questions. As your suggestion, we repeated the experiment again and added the corresponding gray value calculation. As follows:

**Fig. 5G** HEK293T cells were co-transfected with Flag-tagged p62 expression plasmid, Myc-tagged E1A expression plasmid, and V5-tagged TRIM23 expression plasmid at various concentrations for 36 hours, followed by western blot analysis.

*[Fig 5L and related text: These data and the author’s interpretation about TRIM23-TBK1-p62 are vague and do not further develop the story. This reviewer suggests removing these data and related text. Alternatively, more accurate interpretations and writing is required (and likely additional data are needed to support this aspect of the study).]*
**R:** Thanks for your comments and questions. As your suggestion, we deleted the results and description of Fig. 5L. Please check.

*[Fig 5K: The loss of binding upon TRIM23 silencing is not very evident. Please quantify these data from three independent blots.]*

**R:** Thanks for your very instructive comments! As your suggestion, we added the quantitative statistical results of three independent experiments. The results are shown as follows:

**Fig. 5K and L** Lysates from HEK293T co-transfected with specific shRNA targeting TRIM23 and plasmids encoding E1A and Flag-p62 were subjected to co-IP assays using an anti-E1A antibody, followed by immunoblot analysis with the indicated antibodies. Quantification of relative p62 levels is shown in (L). ***, *P* <0.001.

**Reviewer #2: None**

**Reviewer #3: (No Response)**

**Part III – Minor Issues: Editorial and Data Presentation Modifications**
Please use this section for editorial suggestions as well as relatively minor modifications of existing data that would enhance clarity.

**Reviewer #1: Minor points:**

*[-Abstract: The conclusion that “p62 facilitates the E1A-p62 interaction independent of ubiquitin signaling and subsequently directs E1A to phagophores for degradation” is made mainly based on the observation that “E1A is not ubiquitinated and TRIM23 did not promote p62 ubiquitination”. The term “ubiquitin signaling” is too vague while “E1A recruitment to phagophores” is not shown in the manuscript. The authors should revise the text by more accurately stating te results (such as “in a E1A ubiquitination-independent manner”).]*
**R:** Thanks for your very instructive comments! According to your suggestion, we have revised it to “TRIM23 recruits the selective autophagy receptor p62 promotes E1A degradation in a E1A ubiquitination-independent manner”. Please check.

*[-Author’s summary: The authors now show that TRIM23, via its E3 ligase activity, restricts AdV (mis-interpreted previously as an E3-ligase independent role). The authors still wrote that in the author summary that “this study reveals an unconventional antiviral role for TRIM23”. Not sure what they mean by the term “unconventional”. The authors should more accurately state the findings.]*
**R:** Thanks for your very instructive comments! According to your suggestion, we have revised it to “This study reveals that TRIM23 plays an antiviral role independent of its ubiquitination function”. Please check.

*[-Introduction (last paragraph): Remove the term “independent of its canonical ubiquitin-proteasome mediated signaling” from the statement as this function is not canonical for TRIM23’s antiviral function.]*
**R:** Thanks for your suggestion. We have modified it. please check.

*[-Fig 3K: The authors did not provide convincing answers for “why there is higher p62 in samples with higher LC3-II levels. These two readouts don’t fit together in this experiment.]*
**R:** Thanks for your comments and questions. Only detecting the level of p62 cannot accurately determine whether autophagy is increased. Under emergency conditions such as oxidative stress, nutrient deficiency, and viral infection, cells may enhance the ability to remove harmful substances by up-regulating the expression of p62. At this time, the increase of p62 may be accompanied by autophagy activation, which belongs to the adaptive protection response of cells. In this case, the increase of p62 is a compensatory increase in cell response to stress, rather than a direct manifestation of enhanced autophagy. Here, we detected that the increase of p62 level may be a compensatory increase in viral infection with the increased MOI of HAdV-C5.

*[-Fig 2H: The authors still did not perform direct (i.e., in vitro test tube) binding assay here. Therefore, please revise the statement related to this figure.]*
**R:** Thanks for your suggestion. According to your suggestion, we have modified it.

*[-Fig 2J: The authors mentioned in the response that they included a negative control but still they did not include a proper negative control (i.e., only TRIM without E1A) in this repeated binding assay. This needs to be included.]*
**R:** Thanks for your suggestion. Here we want to explain that this co-immunoprecipitation experiment was performed with E1A antibody. We have verified that each truncated mutant is normally expressed in the whole cell lysates. In addition, the negative control of the co-immunoprecipitation group showed that the E1A protein has no non-specific binding protein. And, there are a lot of references is also carried out in this way (References to *PMID:40435148、PMID:40511919, etc*). Thank you for your understanding.

*[Fig 5D: Please add the explanation provided in the response letter also in the main text for clarity.]*
**R:** Thanks for your suggestion. According to your suggestion, we also added an explanation in the main text. Please check.

*[Page 9. The last sentence is incomplete.]*
**R:** Thanks for your suggestion. According to your suggestion, we have updated the sentence to that “confocal microscopy observed an increase in TRIM23 in the cytoplasm during HAdV-C5 infection”. Please check.

*[Page 10 (text related to S3C and S3D): The statement that “IFN-α stimulation can strongly induce type I IFN” is confusing and there is no such data shown by the authors.]*
**R:** Thanks for your suggestion. We have made changes, please check.

*[Page 10 (result title): The term “TRIM23 hijacks autophagy” is not correct as autophagy activation is one of its proven functions.]*
**R:** Thanks for your suggestion. We have revised the sentence to that “TRIM23 activates autophagy to degrade HAdV-C5 E1A”. Please check.

*[Page 11 (first paragraph, last sentence): The term “ubiquitin-independent recognition” is unclear. What do the authors mean?]*
**R:** Thanks for your suggestion. Here we want to express that the presence of TRIM23 does not change the level of E1A ubiquitination modification, excluding the possibility that it regulates E1A protein level through the ubiquitin-proteasome pathway. Now, we have re-corrected the description to make it more accurate. Please check.

*[Page 12, line 1-2: The statement “N-terminal RING finger domain, which mediates the binding of ubiquitin or ubiquitin-like proteins” need to be revised. Also, the cited reference (31) is not relevant.]*

**R:** Thanks for your suggestion. According to your suggestion, we have revised this sentence, updated the references, and re-examined the references of the full text. Please check.

**Reviewer #2: A few minor revisions are required:**
*[1. Western blot paragraph has tense inconsistency: mixed past/present tense (“Prepare whole-cell extracts… After washing with PBS, the membrane was incubated…”), unify experimental method to past tense uniformly.]*

**R:** Thanks for your very instructive comments! According to your suggestion, we unified changed to the past. Please check.

*[2. Stable cell construction: “Use Lipofectamine 2000…” → revise to past tense “Lipofectamine 2000 was used to…” consistent with manuscript method writing norm.]*

**R:** Thanks for your suggestion. We have made changes, please check.

*[3. List all used internal reference and target gene primers into supplementary table in manuscript, current only listed E1A/Hexon/DBP primers, lack GAPDH primer sequence.]*

**R:** Thanks for your suggestion. According to your suggestion, we added *GAPDH* quantitative primer sequence in the material method. Please check.

*[4. Some outdated references and insufficient latest literatures about TRIM-autophagy-adenovirus axis in Introduction and Discussion; supplement recent 3-5 years related high-quality publications.]*

**R:** Thanks for your suggestion. We have supplemented and updated some new references, and checked the full text.

*[5. First paragraph Discussion: “TRIM56 modulates STING-dependent HSV-1” etc descriptions too brief; briefly summarize concrete antiviral autophagy mode of listed TRIM proteins to better highlight novelty of current TRIM23-E1A-p62 pathway.]*

**R:** Thanks for your suggestion. We have supplemented and amended this part of the description. Please check.

*[6. Abbreviation: first appearance of all abbreviations (OAV, MOI, TCID₅₀, CQ, BafA1 etc) have been defined basically, individual rare abbreviation need replenish full name at first occurrence.]*

**R:** Thanks for your suggestion. We have re-checked and modified the abbreviations of the full text. Please check.

**Reviewer #3:** *[Nevertheless, the upstream mechanism responsible for TRIM23 induction remains unclear. Given the authors’ suggestion that E1A may negatively regulate TRIM23 expression, I encourage the authors to expand the Discussion to address this possibility and to discuss potential mechanisms underlying TRIM23 induction during HAdV-C5 infection.]*

**R:** Thanks for your suggestion. We have re-added the discussion section. Please check.
