# Supplementary material for: TRIM23 prevents adenovirus replication by p62-mediated selective autophagic degradation of viral E1A protein
Source: PLoS Pathog. 2026 Sep 8;22(9):e1014578. doi: 10.1371/journal.ppat.1014578 (PMC13577515; doi:10.1371/journal.ppat.1014578)
Supplement: Peer review history

# Peer review: Decision Letter 0

**DOI:** 10.1371/journal.ppat.1014578.r001

**Authors:** Pinghui Feng, Alison Anne McBride

26 Feb 2026

TRIM23 prevents adenovirus replication by p62-mediated selective autophagic degradation of viral E1A protein

PLOS Pathogens

Dear Dr. Sun,

Thank you for submitting your manuscript to PLOS Pathogens. After careful consideration, we feel that it has merit but does not fully meet PLOS Pathogens's publication criteria as it currently stands. Therefore, we invite you to submit a revised version of the manuscript that addresses the points raised during the review process.

Please submit your revised manuscript by Apr 27 2026 11:59PM. If you will need more time than this to complete your revisions, please reply to this message or contact the journal office at plospathogens@plos.org. When you're ready to submit your revision, log on to https://www.editorialmanager.com/ppathogens/ and select the 'Submissions Needing Revision' folder to locate your manuscript file.

Please include the following items when submitting your revised manuscript:

* A letter that responds to each point raised by the editor and reviewer(s). You should upload this letter as a separate file labeled 'Response to Reviewers'. This file does not need to include responses to any formatting updates and technical items listed in the 'Journal Requirements' section below.

* A marked-up copy of your manuscript that highlights changes made to the original version. You should upload this as a separate file labeled 'Revised Manuscript with Track Changes'.

* An unmarked version of your revised paper without tracked changes. You should upload this as a separate file labeled 'Manuscript'.

If you would like to make changes to your financial disclosure, competing interests statement, or data availability statement, please make these updates within the submission form at the time of resubmission. Guidelines for resubmitting your figure files are available below the reviewer comments at the end of this letter.

We look forward to receiving your revised manuscript.

Kind regards,

Pinghui Feng

Academic Editor

PLOS Pathogens

Alison McBride

Section Editor

PLOS Pathogens

Editor-in-Chief

PLOS Pathogens

Editor-in-Chief

PLOS Pathogens

orcid.org/0000-0002-7699-2064

**Journal Requirements:**

1) Some material included in your submission may be copyrighted. According to PLOSu2019s copyright policy, authors who use figures or other material (e.g., graphics, clipart, maps) from another author or copyright holder must demonstrate or obtain permission to publish this material under the Creative Commons Attribution 4.0 International (CC BY 4.0) License used by PLOS journals. Please closely review the details of PLOSu2019s copyright requirements here: PLOS Licenses and Copyright. If you need to request permissions from a copyright holder, you may use PLOS's Copyright Content Permission form.

Please respond directly to this email and provide any known details concerning your material's license terms and permissions required for reuse, even if you have not yet obtained copyright permissions or are unsure of your material's copyright compatibility. Once you have responded and addressed all other outstanding technical requirements, you may resubmit your manuscript within Editorial Manager.

Potential Copyright Issues:

- Please confirm (a) that you are the photographer of Figure 7F., or (b) provide written permission from the photographer to publish the photo(s) under our CC BY 4.0 license.

2) Please amend your detailed Financial Disclosure statement. This is published with the article. It must therefore be completed in full sentences and contain the exact wording you wish to be published.

State the initials, alongside each funding source, of each author to receive each grant. For example: "This work was supported by the National Institutes of Health (####### to AM; ###### to CJ) and the National Science Foundation (###### to AM).".

If you did not receive any funding for this study, please simply state: u201cThe authors received no specific funding for this work.u201d

**Reviewers' Comments:**

Reviewer's Responses to Questions

**Part I - Summary**

Please use this section to discuss strengths/weaknesses of study, novelty/significance, general execution and scholarship.

Reviewer #1: Tripartite motif-containing protein 23 (TRIM23) is a unique protein that possesses RING E3 ligase and ARF GTPase activities. TRIM23 has been linked to various cellular processes and has been previously recognized as an important regulator of autophagy during viral infections (mainly herpesvirus but also adenovirus; e.g. PMID: 28871090). In this manuscript submitted to PLOS Pathogens, the authors report that human adenovirus type 5 (HAdV-C5) upregulates TRIM23 in A549 cells. By utilizing multiple approaches, the authors convincingly demonstrate that TRIM23 potently restricts HAdV-C5, which is consistent with a previous report (Sparrer et al., 2017). The authors show that TRIM23 interacts with early protein 1A (E1A) of HAdV-C5 and degrades this protein in an autophagy-dependent manner. The authors propose that TRIM23’s E3 ligase activity is indispensable for E1A degradation as no ubiquitination of E1A was detected. Finally, the authors generated an oncolytic adenovirus (OAV) carrying an shRNA targeting TRIM23 (OAV-shTRIM23) and showed that OAV-shTRIM23 has higher replication fitness and possesses stronger antiproliferative/cytopathic activity in cells, which was also demonstrated in a xenograft mouse model of prostate cancer.

Strengths of the study are the data showing an important role of TRIM23 in HAdV-C5 restriction via autophagy as well as the data showing a higher replication fitness for OAV-shTRIM23 that led to enhanced tumor suppressing ability. However, the data on the proposed molecular mechanism for TRIM23-mediated AdV restriction via E1A mechanism are unconvincing, and some of the experimental designs have flaws. Taken together, despite the novelty of the OAV-shTRIM32 studies and their translational value, there are several experimental issues in the mechanistic studies (see specific comments below) that decreased the overall quality of the manuscript.

General major comments:

1.The conclusions that “TRIM23 promotes E1A degradation through a mechanism independent of its canonical E3 ligase activity” and “TRIM23 acts as an adaptor for p62-E1A interaction” are not convincingly supported by the data. This part of the manuscript requires extensive additional experimentation.

2.The authors show that TRIM23, via its autophagy activity, degrades E1A. However, the proposed mechanism clashes with the authors’ own functional data (i.e., TRIM23 truncation proteins that are autophagy defective can still restrict HAdV-C5) as well as with the previously published mechanism for TRIM23-mediated autophagy where its RING E3 ligase activates via auto-K27-linked ubiquitination the ARF GTPase activity, which is important for relocalizing TRIM23 onto autophagosomes where it binds to and triggers TBK1-mediated p62 phosphorylation, ultimately inducing antiviral autophagy (in herpesvirus infection). It is likely that the same mechanism of TRIM23-mediated autophagy induction may also apply to HAdV infection.

3.There are substantial flaws and inconsistencies in the experiment design and data interpretation (see specific comments below).

Reviewer #2: Sun et al have identified Trim23 as a new adenovirus E1A interacting protein. Trim23 overexpression inhibits HAdV-C5 early gene transcription, early protein expression, genome replication and infectious progeny production. All of these effects seem to arise from Trim23 leading to E1A degradation via autophagy.

Overall, this is an interesting paper with some high-quality data. There is a lot of very solid data in this manuscript and the conclusion mostly seem well justified. However, many critical details are missing and there are a number of minor problems with the language.

Reviewer #3: The title of this review is “TRIM23 prevents adenovirus replication by p62-mediated selective autophagic degradation of viral E1A protein”.

The manuscript investigates the antiviral role of TRIM23 against human adenovirus type 5 (HAdV-C5), proposing that TRIM23 interacts with the viral protein E1A, promotes its degradation via an autophagosome-dependent mechanism, and suppresses transcription of HAdV-C5 viral genes. While the study presents potentially interesting findings, there is a fundamental conceptual issue that significantly weakens the overall premise of the work.

The entire story is built upon the initial observation that TRIM23 is upregulated in E1A-overexpressing cells (first result part; through proteomic analysis….). This finding serves as the primary rationale and conceptual starting point of the manuscript. In other words, the authors justify the investigation of TRIM23 as an antiviral factor based on its induction by E1A. However, this central rationale raises a critical and unresolved paradox.

According to the authors’ model, TRIM23 promotes degradation of E1A and suppresses viral gene transcription, thereby inhibiting viral replication. If this is the case, it is biologically counterintuitive that E1A—a key viral transactivator essential for efficient viral replication—would induce the expression of a host factor that directly limits its own stability and viral gene expression. The manuscript does not provide a convincing mechanistic explanation for this apparent contradiction.

Specifically, it remains unclear whether TRIM23 upregulation is:

1. a direct transcriptional effect mediated by E1A,

2. an indirect consequence of cellular stress or innate immune activation triggered by E1A, or

3. part of a host-driven antiviral feedback response that is independent of viral intention.

Without clarifying this point, the biological logic of the proposed model lacks coherence. The claim that E1A induces TRIM23, which in turn degrades E1A and suppresses viral transcription, creates a circular and conceptually inconsistent framework. Because this observation constitutes the foundation of the study’s rationale, the failure to resolve this issue undermines the validity of the overall interpretation.

In its current form, the manuscript presents interesting molecular interactions but does not adequately reconcile this fundamental conceptual contradiction. Therefore, I believe the study requires substantial mechanistic clarification before it can be considered for publication. Given the central nature of this issue, I do not recommend publication at this stage.

**********

**Part II – Major Issues: Key Experiments Required for Acceptance**

Please use this section to detail the key new experiments or modifications of existing experiments that should be absolutely required to validate study conclusions.

Generally, there should be no more than 3 such required experiments or major modifications for a "Major Revision" recommendation. If more than 3 experiments are necessary to validate the study conclusions, then you are encouraged to recommend "Reject".

Reviewer #1: Figures 1 and 2:

The authors used complementary approaches and various readouts (e.g., viral titter, IF, and western blot) to demonstrate that TRIM23 restricts HAdV-C5. These data are strong and convincing. Some of the data shown in Figure 1 (e.g., Fig 1B, 1G, 1H) could be moved to extended panels.

The data in Figure 2 (i.e., RT-qPCR measurement of multiple viral genes) support the authors’ conclusion drawn from the data in Figure 1. Instead of presenting these data as a separate figure, the data could be integrated into Figure 1 and/or shown as extended panels.

Figure 3:

Fig 3H and Fig 3K-3M: The authors concluded from these data that “the ARF domain of TRIM23 mediates the physical interaction with HAdV-C5 E1A, and that both the RING domain and ARF domain of TRIM23 contribute to its antiviral effect”. These functional data do not align with the author’s mechanistic data for the proposed “TRIM23-E1A-p62” axis. Particularly, the mechanistic data that are shown later (Fig 4 and 5) clash with the functional data which show that the isolated ARF, delRING, and delARF (all of which by themselves are reportedly not able to activate autophagy) efficiently restrict HAdV-C5. How do the authors explain their effects? Do these truncation proteins also induce degradation of E1A (which is very unlikely), or could they have additional effects on the cell that may indirectly influence virus replication?

Figure 4:

Fig 4A, 4C-F, and 4H: These data strongly suggest that TRIM23-mediated autophagy facilitates degradation of E1A. It has been previously demonstrated that TRIM23 mediates antiviral autophagy via auto-ubiquitination of its ARF domain; this activates TRIM23’s GTPase activity, which is essential for TRIM23’s relocalization to autophagosomes and for TBK1-mediated phosphorylation of the autophagy receptor p62 (PMID: 28871090). The authors should test if a similar mechanism is activated during HAdV infection, and this ultimately leads to p62-mediated E1A degradation.

Fig 4B. Solely based on these observations the authors concluded that TRIM23-mediated E1A degradation does not require its E3 ligase activity. RING E3 ligase activity is known to be essential for TRIM23-mediated autophagy because it activates the ARF GTPase via auto-K27-linked ubiquitination. TRIM23 may or may not ubiquitinate E1A. The authors should first compare the ability of WT TRIM23 and a RING catalytic or delRING mutant in their ability to restrict HAdV and to degrade E1A via autophagy. Moreover, to directly test E1A ubiquitination by TRIM23, the authors should include a positive control.

Fig 4I. These data do not unbiasedly test whether E1A enhances TRIM23-mediated autophagy; the authors need to include an additional control where there is no E1A.

Fig 4J. p62 is generally degraded upon autophagy induction, which the authors also showed in Fig 5H. However, in this figure (4J) the authors show that HAdV-C5 infection upregulates p62. Why is this?

Figure 5:

Fig 5D: A control condition with only E1 and no p62 should be included in this assay.

Fig 5E: Fig 5D and 5E data are somehow redundant.

Fig 5F and 5G: The authors propose that TRIM23 functions as an adaptor. To rigorously test this, the authors should assess p62-E1A interaction in WT and TRIM23 knockout cells.

Fig 5H: It is unclear what conclusion the authors want to draw from these data as there is no E1A included in this assay. What is shown in this figure (i.e., TRIM23 induces degradation of p62) has been previously reported.

Figure 6:

Fig 6D: Opposing to these data, the authors show higher levels of E1A in TRIM23 depleted cells in Figure 1J. Please address this discrepancy.

Figure 7:

Fig 7J and 7L: These data are not consistent with the results shown in Fig 6D.

Reviewer #2: Major issues:

1:No methods or useful description is provided for the mass-spec data shown in Figure 1A. The TRIM23 overexpressing cell line is also not described. The mass-spec data either needs to be publicly archived or presented as supplementary data.

2:The authors use HEK293T cells in a number of experiments (see figure 3 for example), but the blots never show any endogenous E1A in the control transfected/infected cells. This needs to be explained.

3:The generation of the oncolytic virus is a bit vague. What is deleted from the Ad5 genome? Is it expected to replicate in normal cells? Additionally, that point is not addressed at all. Does this oncolytic virus infect non-tumor cells? Does it replicate well in these cells. How safe would this virus be? The authors describe that they focus on better replication in tumor cells, but they also need to address the selectivity of the virus as well as safety.

4:Given that p62 is primarily localized to the cytoplasm, and because TRIM23 is know to target E1A for degradation, and E1A is known to bind p62, the authors might speculate that the association with E1A is indirect and bridged by TRIM23. What domains of TRIM23 are needed to interact with p62? If they are the same as needed for E1A, this would lend some support to this model. Alternatively, are they forming a trimeric complex? This could be demonstrated by serial co-IPs.

Reviewer #3: The title of this review is “TRIM23 prevents adenovirus replication by p62-mediated selective autophagic degradation of viral E1A protein”.

The manuscript investigates the antiviral role of TRIM23 against human adenovirus type 5 (HAdV-C5), proposing that TRIM23 interacts with the viral protein E1A, promotes its degradation via an autophagosome-dependent mechanism, and suppresses transcription of HAdV-C5 viral genes. While the study presents potentially interesting findings, there is a fundamental conceptual issue that significantly weakens the overall premise of the work.

The entire story is built upon the initial observation that TRIM23 is upregulated in E1A-overexpressing cells (first result part; through proteomic analysis….). This finding serves as the primary rationale and conceptual starting point of the manuscript. In other words, the authors justify the investigation of TRIM23 as an antiviral factor based on its induction by E1A. However, this central rationale raises a critical and unresolved paradox.

According to the authors’ model, TRIM23 promotes degradation of E1A and suppresses viral gene transcription, thereby inhibiting viral replication. If this is the case, it is biologically counterintuitive that E1A—a key viral transactivator essential for efficient viral replication—would induce the expression of a host factor that directly limits its own stability and viral gene expression. The manuscript does not provide a convincing mechanistic explanation for this apparent contradiction.

Specifically, it remains unclear whether TRIM23 upregulation is:

1. a direct transcriptional effect mediated by E1A,

2. an indirect consequence of cellular stress or innate immune activation triggered by E1A, or

3. part of a host-driven antiviral feedback response that is independent of viral intention.

Without clarifying this point, the biological logic of the proposed model lacks coherence. The claim that E1A induces TRIM23, which in turn degrades E1A and suppresses viral transcription, creates a circular and conceptually inconsistent framework. Because this observation constitutes the foundation of the study’s rationale, the failure to resolve this issue undermines the validity of the overall interpretation.

In its current form, the manuscript presents interesting molecular interactions but does not adequately reconcile this fundamental conceptual contradiction. Therefore, I believe the study requires substantial mechanistic clarification before it can be considered for publication. Given the central nature of this issue, I do not recommend publication at this stage.

**********

**Part III – Minor Issues: Editorial and Data Presentation Modifications**

Please use this section for editorial suggestions as well as relatively minor modifications of existing data that would enhance clarity.

Reviewer #1: 1. Abstract: Remove the word “directly” from “TRIM23 interacts directly with the viral E1A”, as such conclusion cannot be made based on the cell-based interaction data shown. This requires in vitro binding study using recombinant purified proteins.

2. Introduction: The authors mention certain TRIM proteins with roles in autophagy for which there is only limited literature. By contrast, they omit the literature describing the most well-studied TRIM proteins in viral autophagy, which is TRIM5a and TRIM23. Please also include these. Moreover, the authors should introduce and summarize the previous findings on TRIM23 and its reported role/activation mechanism in autophagy and antiviral defense and cite the relevant literatures (PMID: 25587751; PMID: 33669846; PMID: 28871090; PMID: 40360474

3. Fig 1A. Why is the viral titter lower at 48 h compared to 24 h in Fig 1A, whereas most of the data (Fig. 1C, D, E, J etc.) show that HAdV-C5 replicates efficiently at 48 h compared to 24 h.

4. Please add the molecular markers for all the immunoblots.

5. Fig 1H. Please improve the quality of these images. If possible, please also do quantification.

6. Fig 3H. The authors should include a negative control in this experiment; this is needed to rule out that the proteins are not non-specifically binding to the beads.

7. Fig 3A. The authors should quantify these data (ideally from three independent blots) accounting for the GAPDH that is also increasing over time.

8. Fig 3E. Why are the bands for V5-TRIM23 running differently in WCL vs. IP?

9. Fig 3H. Why does the TRIM23-V5 blot show two bands in both WCL as well as IP?

10. Fig 3L. The interpretation that “there was no significant difference in E1A viral protein compared with the control” seems to be incorrect. The authors should quantify the E1A blot and perform statistical analysis.

11. Fig 5C: These data are not correctly explained/interpreted in the related text in the Results section.

12. Fig 5D: In the first lane, there appears to be a non-specific band in the Myc (E1A) blot. The authors should indicate this in the blot and describe it in the legend. The authors did not include a condition with no E1A in the rest of the figure panels; please include a “no E1A” control.

13. Fig 5E: TRIM23 is not inducing p62 degradation here when tested in the presence of E1A, which is in contrast to the data shown in Fig 5H. Why is this?

14. Fig 6C. The quality of these images should be improved.

15. Discussion: In the statement “TRIM23 exerts both positive and negative regulatory roles in the antiviral type I interferon response [36, 37]”, both references (36 and 37) are not describing TRIM23’s role in IFN responses, but they describe its role in antiviral autophagy. Please correct the sentence.

Reviewer #2: Minor issues

1:There are minor issues with the language. Line 181, sentence is incomplete. Line 161 the word detected should be studied, examined, investigated, etc. Some abbreviations are not defined (DEG, RTCA), etc. The writing is pretty good but needs careful editing.

2:For the figures, use the same font for the panel labels as the rest of the figure (A, B, C, etc)

3:There are lots of references, but they sometimes seem like odd choices (refs 11, 27 are okay but not complete, etc). I suggest going through the citations very carefully.

4:Be consistent with abbreviations. qRT-PCR was used for much of the data, but is called qPCR and other things variably in the manuscript.

5:Some figure legends state the number of times a given experiment was performed, others do not state this. The n for every experiment should be provided in all of the figure legends.

6:I don't think ref 5 is a great choice for this point. Its just a single research paper. No refs for the plasmids used in the HAdV rescue are cited on page 12.

7:As TRIM23 is induced by interferon (IFN), is it simply possible that the HAdV infection is just inducing an IFN response that increases TRIM infection? Not that PML is a TRIM and there is a long history of E1A altering PML bodies (PMID: 13130097, etc).

8:Figs. 3K and 3L. The authors switched the assays here form retrovirus transduction of TRIM23 to transient expression. It is critical that data with the wild-type TRIM23 protein be included alongside the mutant TRIM23s to verify the assay.

9:Figs. 4C and 4H - lighter E1A exposures of the Western blots are needed.

10:Figure 5. " Taken together, these results indicate that TRIM23 accelerates the autophagic degradation of E1A by promoting its interaction with p62." No part of that statement has been conclusively shown, but additionally, they have not looked at the interaction of the TRIM23, E1A, or p62.

11:Fig. 6G- the authors should refer to this data as anti-proliferative effects, not anti-tumor effects. These are in vitro experiments on cell growth.

Reviewer #3: The manuscript investigates the antiviral role of TRIM23 against human adenovirus type 5 (HAdV-C5), proposing that TRIM23 interacts with the viral protein E1A, promotes its degradation via an autophagosome-dependent mechanism, and suppresses transcription of HAdV-C5 viral genes. While the study presents potentially interesting findings, there is a fundamental conceptual issue that significantly weakens the overall premise of the work.

The entire story is built upon the initial observation that TRIM23 is upregulated in E1A-overexpressing cells (first result part; through proteomic analysis….). This finding serves as the primary rationale and conceptual starting point of the manuscript. In other words, the authors justify the investigation of TRIM23 as an antiviral factor based on its induction by E1A. However, this central rationale raises a critical and unresolved paradox.

According to the authors’ model, TRIM23 promotes degradation of E1A and suppresses viral gene transcription, thereby inhibiting viral replication. If this is the case, it is biologically counterintuitive that E1A—a key viral transactivator essential for efficient viral replication—would induce the expression of a host factor that directly limits its own stability and viral gene expression. The manuscript does not provide a convincing mechanistic explanation for this apparent contradiction.

Specifically, it remains unclear whether TRIM23 upregulation is:

1. a direct transcriptional effect mediated by E1A,

2. an indirect consequence of cellular stress or innate immune activation triggered by E1A, or

3. part of a host-driven antiviral feedback response that is independent of viral intention.

Without clarifying this point, the biological logic of the proposed model lacks coherence. The claim that E1A induces TRIM23, which in turn degrades E1A and suppresses viral transcription, creates a circular and conceptually inconsistent framework. Because this observation constitutes the foundation of the study’s rationale, the failure to resolve this issue undermines the validity of the overall interpretation.

In its current form, the manuscript presents interesting molecular interactions but does not adequately reconcile this fundamental conceptual contradiction. Therefore, I believe the study requires substantial mechanistic clarification before it can be considered for publication. Given the central nature of this issue, I do not recommend publication at this stage.

**********

PLOS authors have the option to publish the peer review history of their article (what does this mean?). If published, this will include your full peer review and any attached files.

If you choose “no”, your identity will remain anonymous but your review may still be made public.

**Do you want your identity to be public for this peer review?**  For information about this choice, including consent withdrawal, please see our Privacy Policy.

Reviewer #1: No

Reviewer #2: No

Reviewer #3: No

[NOTE: If reviewer comments were submitted as an attachment file, they will be attached to this email and accessible via the submission site. Please log into your account, locate the manuscript record, and check for the action link "View Attachments". If this link does not appear, there are no attachment files.]

**Figure resubmission:**

**Reproducibility:**

?>

# Author comment: Author response to Decision Letter 1

**DOI:** 10.1371/journal.ppat.1014578.r002

14 May 2026

Attachment Submitted filename: Response to reviewers comments.docx

# Peer review: Decision Letter 1

**DOI:** 10.1371/journal.ppat.1014578.r003

**Authors:** Pinghui Feng, Alison Anne McBride

23 Jul 2026

PPATHOGENS-D-26-00171R1

TRIM23 prevents adenovirus replication by p62-mediated selective autophagic degradation of viral E1A protein

PLOS Pathogens

Dear Dr. Sun,

Thank you for submitting your manuscript to PLOS Pathogens. After careful consideration, we feel that it has merit but does not fully meet PLOS Pathogens's publication criteria as it currently stands. Therefore, we invite you to submit a revised version of the manuscript that addresses the points raised during the review process.

Please submit your revised manuscript by Sep 21 2026 11:59PM. If you will need more time than this to complete your revisions, please reply to this message or contact the journal office at plospathogens@plos.org. When you're ready to submit your revision, log on to https://www.editorialmanager.com/ppathogens/ and select the 'Submissions Needing Revision' folder to locate your manuscript file.

Please include the following items when submitting your revised manuscript:

* A letter that responds to each point raised by the editor and reviewer(s). You should upload this letter as a separate file labeled 'Response to Reviewers'. This file does not need to include responses to any formatting updates and technical items listed in the 'Journal Requirements' section below.

* A marked-up copy of your manuscript that highlights changes made to the original version. You should upload this as a separate file labeled 'Revised Manuscript with Track Changes'.

* An unmarked version of your revised paper without tracked changes. You should upload this as a separate file labeled 'Manuscript'.

If you would like to make changes to your financial disclosure, competing interests statement, or data availability statement, please make these updates within the submission form at the time of resubmission. Guidelines for resubmitting your figure files are available below the reviewer comments at the end of this letter.

As the corresponding author, your ORCID iD is verified in the submission system and will appear in the published article. PLOS supports the use of ORCID, and we encourage all coauthors to register for an ORCID iD and use it as well. Please encourage your coauthors to verify their ORCID iD within the submission system before final acceptance, as unverified ORCID iDs will not appear in the published article. Only the individual author can complete the verification step; PLOS staff cannot verify ORCID iDs on behalf of authors.

We look forward to receiving your revised manuscript.

Kind regards,

Eain A Murphy, Ph.D.

Academic Editor

PLOS Pathogens

Alison McBride

Section Editor

PLOS Pathogens

Sumita Bhaduri-McIntosh

Editor-in-Chief

PLOS Pathogens

orcid.org/0000-0003-2946-9497

Michael Malim

Editor-in-Chief

PLOS Pathogens

orcid.org/0000-0002-7699-2064

**Additional Editor Comments (if provided):**

Dear Dr. Sun,

I have been assigned this manuscript as the initial academic editor was unavailable. For that we do apologize for the extended time needed to dedicate the proper resources for evaluating this manuscript. It is apparent that the manuscript has been significantly improved upon modification. That being said, there still remains some concerns that the editorial board of PLoS Pathogens feel can be readily addressed in terms of presentation of the work, tempering some of the stronger statements of conclusion and clarification of the data and presented work. I am confident that this manuscript will be well received with addressing the concerns raised in this review.

Sincerely,

Eain A. Murphy Ph.D.

**Journal Requirements:**

If the reviewer comments include a recommendation to cite specific previously published works, please review and evaluate these publications to determine whether they are relevant and should be cited. There is no requirement to cite these works unless the editor has indicated otherwise.

1) PLOS now requires that authors provide the original uncropped and unadjusted images underlying all blot or gel results reported in a submission’s figures or Supporting Information files. This policy and the journal’s other requirements for blot/gel reporting and figure preparation are described in detail at https://journals.plos.org/plosone/s/figures#loc-blot-and-gel-reporting-requirements and https://journals.plos.org/plosone/s/figures#loc-preparing-figures-from-image-files.

When you submit your revised manuscript, please ensure that your figures adhere fully to these guidelines and provide the original underlying images for all blot or gel data reported in your submission. See the following link for instructions on providing the original image data: https://journals.plos.org/plosone/s/figures#loc-original-images-for-blots-and-gels.

In your cover letter, please note whether your blot/gel image data are in Supporting Information or posted at a public data repository, provide the repository URL if relevant, and provide specific details as to which raw blot/gel images, if any, are not available. Email us at customercare@plos.org if you have any questions

**Reviewers' Comments:**

Reviewer's Responses to Questions

**Part I - Summary**

Please use this section to discuss strengths/weaknesses of study, novelty/significance, general execution and scholarship.

Reviewer #1: TRIM23 has been previously shown to induce antiviral autophagy that depends on its RING E3 ligase activity. The authors mis-interpreted in the first submission (partly based on insufficient experimental evidence) that TRIM23, in an E3-ligase activity independent manner, facilitates autophagic degradation of E1A. In the revised manuscript, the authors have performed additional experiments (demonstration of autoubiquitination of TRIM23, inclusion of E3 ligase deficient mutants in their assay, etc.) to systematically assess the mechanism for TRIM23-mediated autophagic degradation of E1A. Based on their new data, the authors have concluded that “TRIM23 promotes E1A degradation through a mechanism dependent of its canonical E3 ligase activity”. The authors also addressed several other concerns.

However, unfortunately, there are still substantial issues and inconsistencies in the data and data interpretation, manuscript writing, and the conclusions drawn.

Reviewer #2: None

Reviewer #3: The title of this review is “TRIM23 prevents adenovirus replication by p62-mediated selective autophagic degradation of viral E1A protein”.

The authors have satisfactorily addressed my major concern regarding the apparent conceptual inconsistency between E1A and TRIM23. The revised text no longer implies that E1A directly induces TRIM23 expression, and the newly provided infection kinetics support the conclusion that TRIM23 is induced during HAdV-C5 infection. In addition, the mechanistic data supporting TRIM23-mediated autophagic degradation of E1A are convincing.

**********

**Part II – Major Issues: Key Experiments Required for Acceptance**

Please use this section to detail the key new experiments or modifications of existing experiments that should be absolutely required to validate study conclusions.

Generally, there should be no more than 3 such required experiments or major modifications for a "Major Revision" recommendation. If more than 3 experiments are necessary to validate the study conclusions, then you are encouraged to recommend "Reject".

Reviewer #1: Major comments:

Fig 4A-C: The authors show in their data that WT-TRIM23 potently restricts AdV. While delRING-TRIM23 mostly loses its antiviral activity, delARF-TRIM23 still shows potent antiviral activity (comparable to WT-TRIM23). However, the authors interpret (on page 12, first paragraph) that the “antiviral effect is primarily mediated by RING domain and requires the presence of the ARF domain”, the latter is not supported by their data (although they show marginal antiviral activity of isolated ARF domain). Importantly, the observation that “del-ARF still retains antiviral activity” and “E1A interacts with TRIM23 via its ARF domain” do not support their working model and conclusions.

Fig 4E-H: Moreover, the authors here show that both delRING and delARF mutants (but not BC-V5, and ARF-V5) can efficiently degrade E1A; these data create additional inconsistencies. The authors did not correctly interpret this data (i.e., they wrote that only BC-V5 failed to degrade, implying that ARF-V5 still degrades, the latter is not evident).

Importantly, the authors again conclude that “both the RING domain and ARF domain of TRIM23 contribute to its antiviral effect”.

Fig 5G: Degradation of E1A by p62 is not evident (2nd vs. 3rd lane) and the effect of TRIM23 is marginal. These observations are not consistent with the authors' other data shown (e.g., Fig. 5E, 5F).

Fig 5L and related text: These data and the author’s interpretation about TRIM23-TBK1-p62 are vague and do not further develop the story. This reviewer suggests removing these data and related text. Alternatively, more accurate interpretations and writing is required (and likely additional data are needed to support this aspect of the study).

Fig 5K: The loss of binding upon TRIM23 silencing is not very evident. Please quantify these data from three independent blots.

Reviewer #2: None

Reviewer #3: (No Response)

**********

**Part III – Minor Issues: Editorial and Data Presentation Modifications**

Please use this section for editorial suggestions as well as relatively minor modifications of existing data that would enhance clarity.

Reviewer #1: Minor points:

-Abstract: The conclusion that “p62 facilitates the E1A-p62 interaction independent of ubiquitin signaling and subsequently directs E1A to phagophores for degradation” is made mainly based on the observation that “E1A is not ubiquitinated and TRIM23 did not promote p62 ubiquitination”. The term “ubiquitin signaling” is too vague while “E1A recruitment to phagophores” is not shown in the manuscript. The authors should revise the text by more accurately stating te results (such as “in a E1A ubiquitination-independent manner”).

-Author’s summary: The authors now show that TRIM23, via its E3 ligase activity, restricts AdV (mis-interpreted previously as an E3-ligase independent role). The authors still wrote that in the author summary that “this study reveals an unconventional antiviral role for TRIM23”. Not sure what they mean by the term “unconventional”. The authors should more accurately state the findings.

-Introduction (last paragraph): Remove the term “independent of its canonical ubiquitin-proteasome mediated signaling” from the statement as this function is not canonical for TRIM23’s antiviral function.

-Fig 3K: The authors did not provide convincing answers for “why there is higher p62 in samples with higher LC3-II levels. These two readouts don’t fit together in this experiment.

-Fig 2H: The authors still did not perform direct (i.e., in vitro test tube) binding assay here. Therefore, please revise the statement related to this figure.

-Fig 2J: The authors mentioned in the response that they included a negative control but still they did not include a proper negative control (i.e., only TRIM without E1A) in this repeated binding assay. This needs to be included.

Fig 5D: Please add the explanation provided in the response letter also in the main text for clarity.

Page 9. The last sentence is incomplete.

Page 10 (text related to S3C and S3D): The statement that “IFN-α stimulation can strongly induce type I IFN” is confusing and there is no such data shown by the authors.

Page 10 (result title): The term “TRIM23 hijacks autophagy” is not correct as autophagy activation is one of its proven functions.

Page 11 (first paragraph, last sentence): The term “ubiquitin-independent recognition” is unclear. What do the authors mean?

Page 12, line 1-2: The statement “N-terminal RING finger domain, which mediates the binding of ubiquitin or ubiquitin-like proteins” need to be revised. Also, the cited reference (31) is not relevant.

Reviewer #2: A few minor revisions are required:

1. Western blot paragraph has tense inconsistency: mixed past/present tense (“Prepare whole-cell extracts… After washing with PBS, the membrane was incubated…”), unify experimental method to past tense uniformly.

2. Stable cell construction: “Use Lipofectamine 2000…” → revise to past tense “Lipofectamine 2000 was used to…” consistent with manuscript method writing norm.

3. List all used internal reference and target gene primers into supplementary table in manuscript, current only listed E1A/Hexon/DBP primers, lack GAPDH primer sequence.

4. Some outdated references and insufficient latest literatures about TRIM-autophagy-adenovirus axis in Introduction and Discussion; supplement recent 3–5 years related high-quality publications.

5. First paragraph Discussion: “TRIM56 modulates STING-dependent HSV-1” etc descriptions too brief; briefly summarize concrete antiviral autophagy mode of listed TRIM proteins to better highlight novelty of current TRIM23-E1A-p62 pathway.

6. Abbreviation: first appearance of all abbreviations (OAV, MOI, TCID₅₀, CQ, BafA1 etc) have been defined basically, individual rare abbreviation need replenish full name at first occurrence.

Reviewer #3: Nevertheless, the upstream mechanism responsible for TRIM23 induction remains unclear. Given the authors’ suggestion that E1A may negatively regulate TRIM23 expression, I encourage the authors to expand the Discussion to address this possibility and to discuss potential mechanisms underlying TRIM23 induction during HAdV-C5 infection.

**********

PLOS authors have the option to publish the peer review history of their article (what does this mean?). If published, this will include your full peer review and any attached files.

If you choose “no”, your identity will remain anonymous but your review may still be made public.

**Do you want your identity to be public for this peer review?**  For information about this choice, including consent withdrawal, please see our Privacy Policy.

Reviewer #1: No

Reviewer #2: No

Reviewer #3: No

[NOTE: If reviewer comments were submitted as an attachment file, they will be attached to this email and accessible via the submission site. Please log into your account, locate the manuscript record, and check for the action link "View Attachments". If this link does not appear, there are no attachment files.]

**Figure resubmission:**

**Reproducibility:**

To enhance the reproducibility of your results, we recommend that authors of applicable studies deposit laboratory protocols in protocols.io, where a protocol can be assigned its own identifier (DOI) such that it can be cited independently in the future. Additionally, PLOS ONE offers an option to publish peer-reviewed clinical study protocols. Read more information on sharing protocols at https://plos.org/protocols?utm_medium=editorial-email&utm_source=authorletters&utm_campaign=protocols

# Author comment: Author response to Decision Letter 2

**DOI:** 10.1371/journal.ppat.1014578.r004

23 Aug 2026

Attachment Submitted filename: Response_to_reviewers_comments_auresp_2.docx

# Editor report: Decision Letter 2

**DOI:** 10.1371/journal.ppat.1014578.r005

**Authors:** Pinghui Feng, Alison Anne McBride

27 Aug 2026

Dear Sun,

We are pleased to inform that your manuscript, "TRIM23 prevents adenovirus replication by p62-mediated selective autophagic degradation of viral E1A protein", has been editorially accepted for publication at PLOS Pathogens.

Before your manuscript can be formally accepted and sent to production, you will need to complete our formatting changes, which you will receive by email within a week. Please note that your manuscript will not be scheduled for publication until you have made the required changes.

IMPORTANT NOTES

(1) Copyediting and Proofreading: The corresponding author will receive a typeset proof for review, to ensure errors have not been introduced during production. Please review the PDF proof of your manuscript carefully, as this is the last chance to correct any errors. Please note that major changes, or those which affect the scientific understanding of the work, will likely cause delays to the publication date of your manuscript.

(2) Appropriate Figure Files: Please remove all name and figure # text from your figure files. Please also take this time to check that your figures are of high resolution, which will improve the readbility of your figures and help expedite your manuscript's publication. Please note that figures must have been originally created at 300dpi or higher. Do not manually increase the resolution of your files. For instructions on how to properly obtain high quality images, please review our Figure Guidelines, with examples at: http://journals.plos.org/plospathogens/s/figures.

We encourage you to use PACE (http://pace.apexcovantage.com/), a figure-checking tool which helps authors ensure all figures meet PLOS requirements so that figures can publish in the highest quality. PACE is a digital diagnostic and conversion tool for figure files. It will provide information about any failed check(s) and, if able, will automatically convert the figure into an acceptable file that passes quality checks.

To use PACE, you must first register as a user. Then, login and navigate to the UPLOAD tab, where you will find detailed instructions on how to use the tool.

(3) Striking Image: Please upload a striking still image to accompany your article if one is available (you can include a new image or an existing one from within your manuscript). Should your paper be accepted, this image will be considered for our monthly issue image and may also appear on our website to feature your article. Please upload this as a separate file, selecting "striking image" as the file type upon upload. Please also include a separate "Other" file with a caption, including credits and any potential copyright information. Please do not include the caption in the main article file. If your image is from someone other than yourself, please ensure that the artist has read and agreed to the terms and conditions of the Creative Commons Attribution License at http://journals.plos.org/plospathogens/s/content-license. Please note that PLOS cannot publish copyrighted images.

(4) Press Release or Related Media: If you or your institution will be preparing press materials for this manuscript, please inform our press team in advance at plospathogens@plos.org as soon as possible. We ask that you contact us within one week to plan ahead of our fast Production schedule. If you need to know your paper's publication date for related media purposes, you must coordinate with our press team, and your manuscript will remain under a strict press embargo until the publication date and time.

(5) Update your Profile Information: Now that your manuscript has been provisionally accepted, please log into Editorial Manager and update your profile, if needed. Go to http://www.editorialmanager.com/ppathogens, log in, and click on the "Update My Information" link at the top of the page. Please update your user information to ensure an efficient production and billing process.

(6) LaTeX users only: Our staff will ask you to upload a TEX file in addition to the PDF before the paper can be sent to typesetting, so please carefully review our Latex Guidelines http://journals.plos.org/plospathogens/s/latex in the meantime.

Thank you again for supporting Open Access publishing; we are looking forward to publishing your work in PLOS Pathogens.

Best regards,

Eain A Murphy, Ph.D.

Academic Editor

PLOS Pathogens

Alison McBride

Section Editor

PLOS Pathogens

Dominique Soldati-Favre

Editor-in-Chief

PLOS Pathogens

***********************************************************

Dear Dr. Sun,

Thank you for your resubmission to PLoS Pathogens. The editorial staff has evaluated this new offering and have come to the decision of ACCEPT without any further review. Congratulations on a nice body of work. You and your team should be proud of this study.

Cheers,

Eain Murphy PhD

# Editor report: Acceptance letter

**DOI:** 10.1371/journal.ppat.1014578.r006

**Authors:** Pinghui Feng, Alison Anne McBride

Dear Sun,

We are delighted to inform you that your manuscript, "TRIM23 prevents adenovirus replication by p62-mediated selective autophagic degradation of viral E1A protein," has been formally accepted for publication in PLOS Pathogens.

We have now passed your article onto the PLOS Production Department who will complete the rest of the pre-publication process. All authors will receive a confirmation email upon publication.

Thank you again for supporting open-access publishing; we are looking forward to publishing your work in PLOS Pathogens.

Best regards,

Dominique Soldati-Favre

Editor-in-Chief

PLOS Pathogens
